# Metabolomic ageing across mental and behavioural disorders

**DOI:** 10.1136/bmjment-2025-302181

**Published:** 2026-06-23

**Authors:** Julian Mutz, Lachlan Gilchrist, Andrea G Allegrini, Sandra Sanchez Roige, Cathryn M Lewis

**Affiliations:** 1 Social, Genetic and Developmental Psychiatry Centre, Institute of Psychiatry, Psychology & Neuroscience, King’s College London, London, England, UK; 2 Perron Institute for Neurological and Translational Science, Perth, Western Australia, Australia; 3 Centre for Preventive Neurology, Wolfson Institute of Population Health, Queen Mary’s University of London, London, UK; 4 Department of Clinical, Educational and Health Psychology, Division of Psychology and Language Sciences, University College London, London, UK; 5 Department of Psychiatry/Institute for Genomic Medicine, University of California San Diego, La Jolla, California, USA; 6 Division of Genetic Medicine, Vanderbilt University Medical Center, Nashville, Tennessee, USA; 7 Department of Medical and Molecular Genetics, Faculty of Life Sciences & Medicine, King’s College London, London, UK

**Keywords:** Mental Health, Psychopathology, Psychiatry, Genetics, Behavioral

## Abstract

**Background:**

Individuals with mental disorders face excess morbidity and premature mortality. Accelerated ageing has been proposed as a contributing mechanism but population-scale evidence across diverse diagnoses is limited.

**Objective:**

To examine whether metabolomic ageing differs across mental disorders and whether associations vary by sex, age group and genetic liability.

**Methods:**

Using plasma metabolomic profiles from UK Biobank participants, we applied a metabolomic ageing clock (MileAge) to estimate disorder-specific differences between metabolite-predicted and chronological age. Mental disorders were ascertained from health records and self-reported physician diagnoses. We analysed nine diagnostic groups and 45 individual disorders and assessed sex and age group differences and associations with polygenic scores.

**Findings:**

Among 225 212 participants (54% female; mean age 56.97), 38 524 had a diagnosis preceding baseline. Substance use, psychotic, affective and neurotic disorders were associated with a metabolite-predicted age older than chronological age, largest for psychosis (β=0.556, 95% CI 0.250 to 0.861, p<0.001). Obsessive-compulsive and eating disorders were associated with a metabolite-predicted age younger than chronological age. Several associations were stronger in males and in individuals aged <65 years. Higher genetic liability to depression, autism and attention-deficit/hyperactivity disorder predicted an older metabolomic age (β range=0.020 to 0.047), whereas polygenic scores for psychosis and tobacco use disorder predicted a younger metabolomic age (β range=−0.023 to −0.040). For obsessive-compulsive disorder and anorexia nervosa, clinical and genetic associations indicated younger metabolomic ageing.

**Conclusions:**

Metabolomic ageing in mental disorders is heterogeneous. While many disorders are associated with an older biological age, some are linked to a younger biological age. Divergence between genetic liability and clinical phenotypes suggests that non-genetic factors shape biological ageing differences.

**Clinical implications:**

Biological age should not be assumed to uniformly exceed chronological age across mental disorders. Sex and age-specific approaches could improve understanding of biological ageing processes in psychiatry.

**WHAT IS ALREADY KNOWN ON THIS TOPIC**

- Prior studies have linked mental disorders to ageing markers, such as shorter telomeres, greater frailty and altered brain structure, yet evidence for metabolomic ageing is largely restricted to mood and anxiety disorders. Large-scale, population-based studies spanning a broad range of diagnoses and evaluating sex or age group differences and genetic liability alongside clinical phenotypes are scarce.

**WHAT THIS STUDY ADDS**

- In 225 000 UK Biobank participants, substance use, psychotic, affective and neurotic disorders were associated with an older metabolite-predicted age, whereas obsessive-compulsive and eating disorders were linked to younger metabolomic ageing. Several associations differed by sex, and associations were generally stronger in individuals aged <65 years. Polygenic scores showed both concordant and divergent patterns relative to diagnoses, indicating that genetic liability does not consistently mirror phenotypic associations with metabolomic ageing.

**HOW THIS STUDY MIGHT AFFECT RESEARCH, PRACTICE OR POLICY**

- These findings indicate that metabolomic ageing varies by diagnosis, sex and age group and should not be assumed to uniformly exceed chronological age across mental disorders. Future work should test disorder, sex and age-specific mechanisms and quantify potentially modifiable contributors (eg, health behaviours, treatment and comorbidity) to metabolomic ageing differences.

## Introduction

 Population ageing is a global health challenge linked to rising chronic disease burden, disability and healthcare costs.^1^ Individuals with mental disorders face increased risks of age-related diseases and reduced life expectancy compared with the general population.^2^ Besides elevated suicide and fatal accident rates^3^ and unhealthy behaviours such as smoking, these disparities may reflect differences in the pace of biological ageing.^4^

While chronological age increases uniformly, individuals vary in the rate at which age-related molecular and cellular damage accumulates, impairing function and increasing disease susceptibility. Mental disorders are associated with age-related functional, molecular and clinical markers, including lower grip strength,^5^ shorter telomeres,^6^ elevated inflammation^7^ and increased frailty.^8^

Advances in computing and the availability of population-scale molecular data have enabled the development of ageing clocks.^9^ Ageing clocks capture the relationship between biological data (eg, plasma metabolites) and a dependent variable (typically chronological age or mortality) and are used to predict a person’s age. The difference between predicted age and chronological age provides a proxy of biological ageing.

Metabolomics, the profiling of small molecules produced by cellular processes, has become a powerful tool in biomedical research.^10^ Metabolite levels provide insights into an organism’s physiological state, reflecting internal and external exposures, including genetic, lifestyle and environmental factors. The pathophysiology of many age-related diseases, including those more prevalent among individuals with mental disorders, involves changes in the metabolome.^11^ Metabolomics may help decipher the molecular mechanisms of ageing^12^ and elucidate how these processes may be altered in mental disorders.

Research at the intersection of metabolomics, biological ageing and psychiatry has recently emerged, with most studies focused on mood and stress or anxiety-related disorders. Robinson *et al* showed that depression, anxiety, post-traumatic stress disorder (PTSD) and heavy alcohol use were associated with older metabolomic ageing profiles in police officers (n=2212; 60.5% male).^13^ However, neither the presence nor severity of depression was associated with metabolomic ageing in a Dutch cohort (n=2910; 66% female),^14^ suggesting that findings may differ by population or methodology. Other mental disorders remain underexplored.

The UK Biobank (UKB) holds one of the largest metabolomics datasets globally. Together with its extensive health record linkage, it represents a key resource to investigate associations between ageing and mental disorders. We developed MileAge, a metabolomic ageing clock trained on plasma metabolite data from over 225 000 individuals. Individuals whose metabolite-predicted age exceeded their chronological age had a higher risk of mortality, greater likelihood of frailty, shorter telomeres and an increased risk of dementia.^15 16^

Here, we examined whether individuals with mental or behavioural disorders differ in metabolomic ageing compared with individuals without such diagnoses. Specifically, we sought to (1) quantify differences in MileAge delta—the difference between metabolite-predicted and chronological age—between individuals with and without mental disorders; (2) determine whether the magnitude of these differences varies across broad diagnostic groups and individual disorders; (3) assess whether associations between mental disorders and metabolomic ageing differ by sex or age group, given established differences in prevalence, clinical course and metabolic correlates and (4) examine whether genetic vulnerability to mental disorders, indexed by polygenic scores, is associated with metabolomic ageing to assess the extent to which observed phenotypic associations might reflect genetic influences versus non-genetic factors. Polygenic scores capture inherited liability to a disorder and therefore provide a complementary perspective on potential mechanisms linking mental disorders and biological ageing. If metabolomic ageing differences primarily reflect downstream consequences of illness, treatment or health-related behaviours, associations would be expected to be stronger for clinical diagnoses than for genetic liability. Conversely, associations with polygenic risk scores would suggest that genetic susceptibility to mental disorders is linked to biological ageing processes more directly.

## Methods

### Study population

The UKB is a community-based health study of over 500 000 individuals aged 37–73 years at baseline.^17^ Individuals registered with the UK National Health Service and living near one of 22 assessment centres were invited to participate. Between 2006 and 2010, participants reported on sociodemographic factors, health behaviours and medical history, underwent physical examination and provided blood and urine samples. Hospital inpatient records are available for most participants, and primary care records are available for approximately 230 000 participants.

### Mental and behavioural disorders

Mental and behavioural disorders were identified using UKB data fields 130 836–130 843 (showcase category 1712), which integrate primary care, hospital inpatient, death registry and self-reported physician diagnosis data. International Classification of Diseases, 10th revision (ICD-10) codes were extracted from hospital and death registry data. ICD-9 codes from hospital records and Read v2 or CTV3 codes from primary care were mapped to ICD-10. Self-reported physician diagnoses from baseline were also mapped to ICD-10, with occurrence dates interpolated as mid-year. To reduce potential reverse causality, only diagnoses with a first recorded occurrence prior to baseline were included. We considered the following ICD-10 codes: F10–F19 (mental and behavioural disorders due to psychoactive substance use), F20–F29 (schizophrenia, schizotypal and delusional disorders), F30–F39 (mood (affective) disorders), F40–F48 (neurotic, stress-related and somatoform disorders), F50–F59 (behavioural syndromes associated with physiological disturbances and physical factors), F60–F69 (disorders of adult personality and behaviour), F70–F79 (intellectual disability), F80–F89 (disorders of psychological development), F90–F98 (behavioural and emotional disorders with onset usually occurring in childhood and adolescence) and F99 (unspecified mental disorder). Organic mental disorders (F00–F09) were excluded due to their distinct nature and clinical profile. Diagnoses were not mutually exclusive; individuals with multiple diagnoses were included in all relevant analyses. A binary ‘any disorder’ phenotype was also derived, defined as having at least one ICD-10 code within F10–F99. The comparison group comprised individuals without any recorded mental disorder.

### Metabolomic ageing (MileAge) clock

Metabolomic biomarkers were quantified using nuclear magnetic resonance (NMR) spectroscopy on non-fasting plasma samples collected at baseline. The Nightingale Health platform ascertains 168 circulating metabolites in absolute concentration units using a standardised high-throughput protocol.^10^ Technical variation was removed using the ‘ukbnmr’ R package (algorithm v2).^18^ This pipeline corrects for known sources of technical and batch-related variation, including spectrometer drift, plate effects and sample measurement order. In a prior study,^15^ we developed a metabolomic clock using a Cubist rule-based regression model. Individual-level age predictions were aggregated from the ten test sets of the outer loop of the nested cross-validation to avoid overfitting. Metabolomic age (MileAge) delta represents the age-bias-adjusted difference between metabolite-predicted and chronological age (in years), with positive values indicating an older biological ageing profile.^15^ MileAge captures age-related variation across multiple metabolic pathways, including lipid metabolism, amino acid profiles and inflammation-related markers, reflecting genetic, environmental and behavioural influences on systemic physiology. Although trained to predict chronological age, MileAge was validated against multiple health and ageing markers, supporting its interpretation as a proxy for biological ageing rather than purely a predictor of chronological time. The Cubist rule-based regression model was selected because it was most strongly associated with multiple health and ageing markers, including frailty and self-rated health, outperforming many other machine learning algorithms. We focused on metabolomic ageing because large-scale NMR metabolomics data were available for over 270 000 UKB participants, enabling analyses across diverse psychiatric diagnoses, whereas comparable proteomic data are currently available only for ~50 000 participants, with broader coverage expected in future releases.

### Polygenic scores

Genotype quality control steps are described in online supplemental file. Polygenic scores for mental disorders were calculated using the GenoPred pipeline.^19^ Each score aligned broadly with the ICD-10 codes reported above. Genome-wide association study (GWAS) summary statistics were obtained from FinnGen, the Psychiatric Genomics Consortium and other sources (online supplemental table S1), predominantly from individuals of European ancestry. There was no sample overlap with UKB. Linkage disequilibrium was estimated using a combined 1000 Genomes Project phase 3^20^ and Human Genome Diversity Project^21^ reference panel, restricted to 1 204 449 HapMap3 variants. MegaPRS implemented in LDAK V.5.1 was used for polygenic scoring, applying the BLD-LDAK heritability model, which incorporates single-nucleotide polymorphism weighting based on allele frequency, linkage disequilibrium and functional annotations to improve predictive performance.^22^ The best-performing polygenic score for each disorder phenotype was selected via pseudo-validation to optimise hyperparameters without requiring individual-level data. Scores were calculated within assigned ancestry groups (matching individuals to five populations in the n=3313 reference data with a probability ≥0.95) and scaled to units of SD from each reference population mean.^23^

### Covariates

A directed acyclic graph (DAG) was used to visualise relationships between mental disorders (exposure), MileAge delta (outcome) and potential confounders: chronological age, sex, highest educational/professional qualification, gross annual household income, ethnicity, cohabitation with spouse/partner, neighbourhood deprivation (Townsend deprivation index) and fasting time (online supplemental figure S1). For categorical covariates with missing data, missing values were retained as a separate category in the regression models to preserve the full analytical sample. Although genetic confounding was not included in the DAG, we performed separate analyses to evaluate associations between polygenic scores for mental disorders and MileAge delta. These analyses were additionally adjusted for genotype batch number, assessment centre and the first six genetic principal components (derived within UKB) to account for population stratification. Although MileAge delta is defined relative to chronological age and corrected for age bias, chronological age was included as a covariate in association models to account for residual age-related variation in prediction error and age patterning of mental disorders.

### Exclusion criteria

We excluded females who were pregnant or unsure about pregnancy status (due to gestational changes in metabolite levels), individuals with mismatched genetic and self-reported sex and those with missing or outlier metabolite values (defined as values 4× the IQR from the median). Individuals with a first occurrence date after the baseline assessment, with no event date or with implausible dates (prior to, matching or in the same calendar year as their birth date or beyond the follow-up period) were also excluded.

### Statistical analyses

Analyses were performed in R (V.4.3.0). Descriptive statistics included means and SD or counts and percentages. Differences in MileAge delta (in years) between individuals with mental disorders and the comparison group were estimated using ordinary least squares regression (MileAge delta~diagnosis+covariates). We fitted a minimally adjusted model that included chronological age and sex (Model 1) and a fully adjusted model that also included education, income, ethnicity, cohabitation, deprivation and fasting time (Model 2). MileAge delta was the dependent variable in all models. We first examined broad diagnostic groups (F10–F99 and subgroups F10–F19 through F99), followed by 45 individual diagnoses with a minimum sample size of 10 individuals per diagnosis. The p values were adjusted for multiple testing using the Benjamini-Hochberg method, with a two-tailed test and a false discovery rate of 5%. To explore sex and age differences, we (1) performed similar analyses in males and females and individuals below and above age 65 years (the current UK retirement age) separately and (2) fitted mental disorder-by-sex and by-age group cross-product interaction terms. Associations between polygenic scores for mental disorders and MileAge delta were estimated using ordinary least squares regression (MileAge delta~polygenic score+covariates). These analyses were performed across the full sample and within ancestry groups.

### Sensitivity analyses

To assess the robustness of phenotypic associations, we excluded individuals with mental disorders identified only from self-reported physician diagnosis data due to potential recall bias. Given that only 45% of the sample had linked primary care data, we also performed analyses within this subset to reduce misclassification bias. In response to peer review, we also fitted nested models adjusting for smoking status and body mass index (BMI) in addition to the Model 2 covariates: Model 2+smoking, Model 2+BMI and Model 2+smoking+BMI. Smoking status was modelled as a categorical variable (never, previous, current) and BMI as a continuous variable.

### Psychiatric comorbidity analyses

To evaluate the impact of diagnostic overlap, we conducted two additional analyses. First, we created mutually exclusive diagnostic variables at both the ICD-10 group and individual diagnosis levels by retaining only individuals with a single recorded mental or behavioural disorder prior to baseline and setting diagnoses to missing for individuals with more than one diagnosis. These analyses assessed whether associations observed in the primary analyses were robust when restricting to individuals without psychiatric comorbidity. Second, we quantified overall psychiatric comorbidity by summing the number of distinct ICD-10 diagnostic groups and the number of individual ICD-10 diagnoses recorded prior to baseline. These counts were categorised to ensure adequate sample sizes and modelled as categorical exposures, with individuals without any mental disorder as the reference group. Associations between comorbidity burden and metabolomic ageing were estimated using the same regression framework as in the primary analyses.

## Results

### Sample characteristics

Of 274 315 participants with metabolomic data, 247 861 had complete data. After excluding individuals with possible pregnancy, discordant genetic and self-reported sex or outlier metabolite values, the final sample comprised 225 212 participants (54% female; mean age=56.97 years, SD=8.10) (table 1; online supplemental figure S2). Of these, 38 524 (17.1%) had at least one mental disorder diagnosis; the remaining 186 688 (82.9%) served as the comparison group. The most common diagnostic categories were substance use (5%, n=11 161), affective (9%, n=20 360) and neurotic disorders (6.2%, n=13 916).

**Table 1:** Sample characteristics

	Analytical sample		
	No diagnosis (n=1 86 688)	Any diagnosis (n=38 524)	Full sample (n=2 25 212)
Age, mean (SD)	57.08 (8.13)	56.45 (7.95)	56.97 (8.10)
Sex			
Female	98 456 (52.7%)	23 075 (59.9%)	121 531 (54%)
Male	88 232 (47.3%)	15 449 (40.1%)	103 681 (46%)
Ethnicity			
White	176 439 (94.5%)	36 981 (96%)	213 420 (94.8%)
Mixed	1017 (0.5%)	234 (0.6%)	1251 (0.6%)
Black	2841 (1.5%)	339 (0.9%)	3180 (1.4%)
Asian	3371 (1.8%)	496 (1.3%)	3867 (1.7%)
Chinese	592 (0.3%)	48 (0.1%)	640 (0.3%)
Other	1601 (0.9%)	255 (0.7%)	1856 (0.8%)
Missing^*^	827 (0.4%)	171 (0.4%)	998 (0.4%)
Highest qualification			
None	30 884 (16.5%)	7851 (20.4%)	38 735 (17.2%)
O levels/general CSEs/CSEs	50 076 (26.8%)	10 624 (27.6%)	60 700 (27%)
A levels/National Vocational Qualification/Higher National Diploma/Higher National Certificate^†^	42 756 (22.9%)	8768 (22.8%)	51 524 (22.9%)
Degree	60 859 (32.6%)	10 819 (28.1%)	71 678 (31.8%)
Missing^*^	2113 (1.1%)	462 (1.2%)	2575 (1.1%)
Household income^‡^			
Very low	33 380 (17.9%)	10 499 (27.3%)	43 879 (19.5%)
Low	40 867 (21.9%)	8803 (22.9%)	49 670 (22.1%)
Medium	42 571 (22.8%)	7832 (20.3%)	50 403 (22.4%)
High	33 854 (18.1%)	4835 (12.6%)	38 689 (17.2%)
Very high	8833 (4.7%)	952 (2.5%)	9785 (4.3%)
Missing^*^	27 183 (14.6%)	5603 (14.5%)	32 786 (14.6%)
Cohabitation			
With partner	139 874 (74.9%)	24 765 (64.3%)	164 639 (73.1%)
Single	14 242 (7.6%)	4108 (10.7%)	18 350 (8.1%)
Missing^*^	32 572 (17.4%)	9651 (25.1%)	42 223 (18.7%)
Townsend deprivation			
Q1	39 991 (21.4%)	6735 (17.5%)	46 726 (20.7%)
Q2	39 381 (21.1%)	7131 (18.5%)	46 512 (20.7%)
Q3	37 660 (20.2%)	7508 (19.5%)	45 168 (20.1%)
Q4	36 186 (19.4%)	7807 (20.3%)	43 993 (19.5%)
Q5	33 261 (17.8%)	9278 (24.1%)	42 539 (18.9%)
Missing^*^	209 (0.1%)	65 (0.2%)	274 (0.1%)
Fasting time			
Less than 8 hours	179 744 (96.3%)	36 686 (95.2%)	216 430 (96.1%)
At least 8 hours	6940 (3.7%)	1838 (4.8%)	8778 (3.9%)
Missing^*^	4 (0%)	0 (0%)	4 (0%)

Numbers shown are counts and percentages unless indicated otherwise.

* Missing data may include ‘do not know’ or ‘prefer not to answer’.

† Also includes other professional qualifications.

‡ Annual household income groups (defined at baseline): very low (<£18 000), low (£18 000–£30 999), middle (£31 000–£51 999), high (£52 000–£100 000) and very high (>£100 000).

CSE, certificate of secondary education; GCSE, general CSE.

### Time between first diagnosis and baseline assessment

Distributions of the time between the first recorded diagnosis and the baseline assessment are shown in online supplemental figure S3. Median time from first diagnosis to baseline ranged from 6.77 years prior (IQR=10.28) for substance use disorders to 18.95 years prior (IQR=27.90) for child and adolescent behavioural/emotional disorders (online supplemental table S2). Data for individual diagnoses are provided in online supplemental table S3 and figure S4-S11.

### Mental/behavioural disorders and MileAge delta

**Figure 1:**
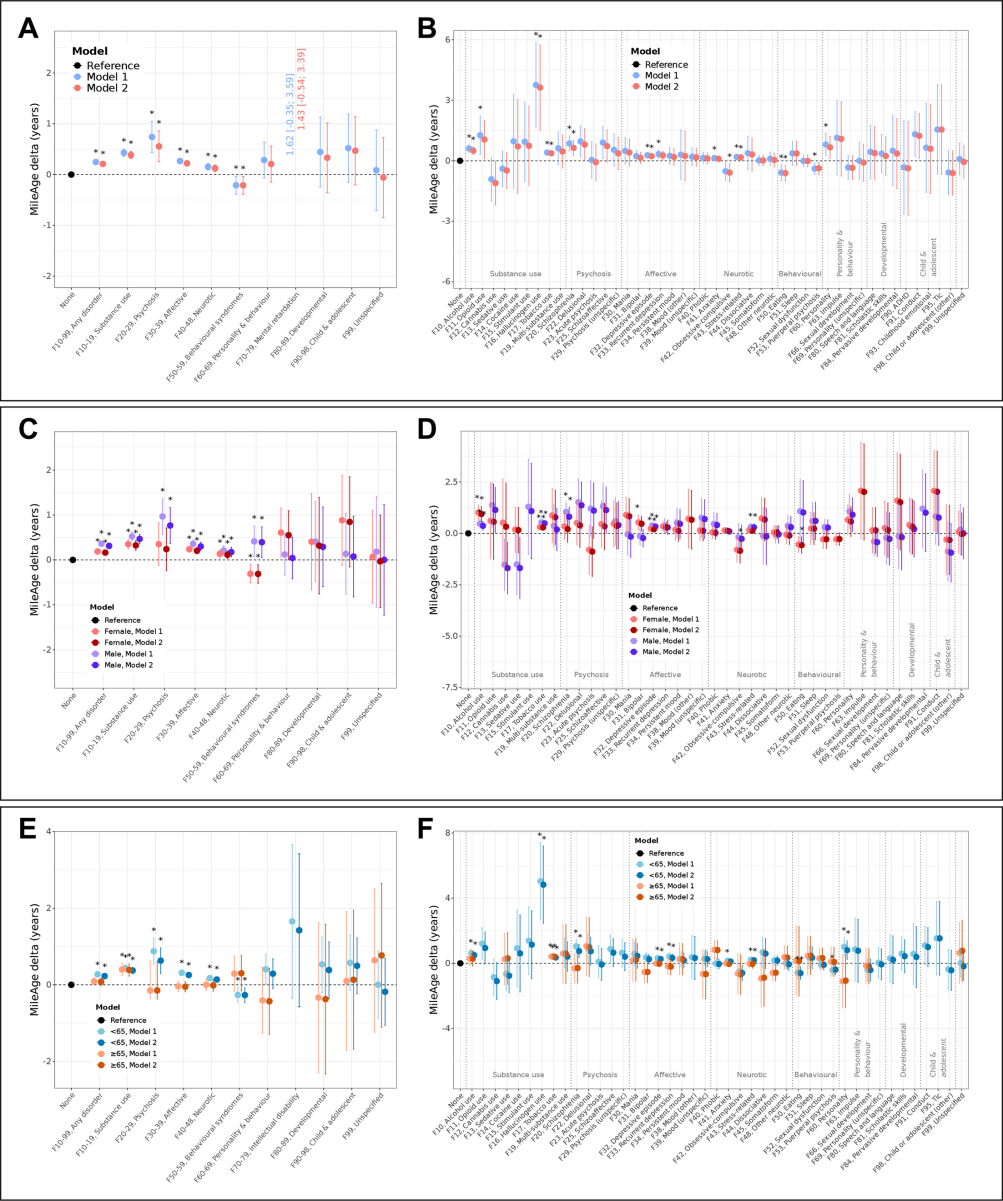
MileAge delta and mental/behavioural disorders. (A) Differences in MileAge delta between individuals with and without mental/behavioural disorders for groups of two-digit ICD-10 codes. (B) Differences in MileAge delta between mental/behavioural disorders for individual two-digit ICD-10 codes. (C) Sex-stratified differences in MileAge delta between mental/behavioural disorders for groups of two-digit ICD-10 codes. (D) Sex-stratified associations between mental/behavioural disorders and MileAge delta for individual two-digit ICD-10 codes. (E) Age-stratified differences in MileAge delta between mental/behavioural disorders for groups of two-digit ICD-10 codes. (F) Age-stratified associations between mental/behavioural disorders and MileAge delta for individual two-digit ICD-10 codes. (A–F) Betas and 95% CIs were estimated using linear regression models. Reference group: individuals without mental/behavioural disorders. (A, B, E and F) Model 1: adjusted for chronological age and sex; Model 2: adjusted for chronological age, sex, ethnicity, cohabitation with spouse/partner, highest educational/professional qualification, annual gross household income, Townsend deprivation index and fasting time. Asterisks indicate statistical significance after correcting p-values for multiple testing using the Benjamini–Hochberg procedure. (C and D) Model 1: adjusted for chronological age; Model 2: adjusted for chronological age, ethnicity, cohabitation with spouse/partner, highest educational/professional qualification, annual gross household income, Townsend deprivation index and fasting time. Asterisks indicate statistical significance after correcting p-values for multiple testing using the Benjamini-Hochberg procedure within each sex. ADHD, attention-deficit/hyperactivity disorder; ICD-10, International Classification of Diseases, 10th revision.

The β coefficients reported below represent the estimated difference in MileAge delta (in years) between individuals with the specified diagnosis and the comparison group without any mental disorder. Across broad diagnostic groups, individuals with substance use, psychotic, affective or neurotic disorders had a metabolite-predicted age (MileAge) exceeding their chronological age (figure 1A). In age- and sex-adjusted models, associations with MileAge delta ranged from β=0.152 (95% CI 0.088 to 0.217, p<0.001) for neurotic disorders to β=0.740 (95% CI 0.436 to 1.045, p<0.001) for psychosis. Conversely, behavioural syndromes were associated with a younger metabolite-predicted age than chronological age (β=−0.210, 95% CI −0.387 to −0.034, p=0.036). All associations remained statistically significant after full adjustment, though most attenuated (table 2).

**Table 2:** Associations between MileAge delta and mental/behavioural disorders

		Model 1 (adjusted age and sex)				Model 2 (full adjustment)			
International Classification of Diseases 10th revision	N	β	95% CI		P value	β	95% CI		P value
None	186 688	Reference				Reference			
F10-99, Any disorder	38 524	**0.247**	**0.206**	**0.288**	**<0.001**	**0.210**	**0.169**	**0.252**	**<0.001**
F10-19, Substance use	11 161	**0.431**	**0.359**	**0.502**	**<0.001**	**0.385**	**0.313**	**0.458**	**<0.001**
F20-29, Psychosis	587	**0.740**	**0.436**	**1.045**	**<0.001**	**0.556**	**0.250**	**0.861**	**<0.001**
F30-39, Affective	20 360	**0.266**	**0.212**	**0.321**	**<0.001**	**0.222**	**0.167**	**0.277**	**<0.001**
F40-48, Neurotic	13 916	**0.152**	**0.088**	**0.217**	**<0.001**	**0.124**	**0.059**	**0.189**	**<0.001**
F50-59, Behavioural syndromes	1763	**−0.210**	**−0.387**	**−0.034**	**0.036**	**−0.211**	**−0.388**	**−0.035**	**0.036**
F60-69, Personality and behaviour	433	0.286	−0.068	0.641	0.178	0.208	−0.146	0.562	0.289
F70-79, Mental retardation	14	1.619	−0.348	3.587	0.178	1.428	−0.537	3.394	0.212
F80-89, Developmental	115	0.444	−0.243	1.131	0.250	0.331	−0.355	1.018	0.378
F90-98, Child and adolescent	120	0.520	−0.152	1.192	0.190	0.469	−0.203	1.140	0.222
F99, Unspecified	86	0.085	−0.708	0.879	0.873	−0.059	−0.852	0.735	0.885

Model 1: adjusted for chronological age and sex; Model 2: adjusted for chronological age, sex, ethnicity, cohabitation with a spouse/partner, highest educational/professional qualification, annual gross household income, neighbourhood deprivation and fasting time. Bold values correspond to statistically significant associations after multiple testing correction. The p values shown are corrected for multiple testing using the Benjamini-Hochberg procedure.

Associations with individual diagnoses are shown in figure 1B. After full adjustment and multiple testing correction, alcohol, hallucinogen and tobacco use disorders, schizophrenia, single-episode depression and stress-related disorders were associated with a metabolite-predicted age exceeding chronological age. Eating disorders were associated with a younger metabolite-predicted age. Full results, including nominal associations, are shown in online supplemental table S4.

### Sex-stratified analyses

Associations between broad diagnostic groups and MileAge delta were generally stronger in males (figure 1C). Behavioural syndromes were associated with a metabolite-predicted age exceeding chronological age in males but with a younger MileAge in females (online supplemental table S5). The association between psychosis and metabolomic ageing was not statistically significant in females (β=0.239, 95% CI −0.235 to 0.712, p=0.431), although it was in males (β=0.765, 95% CI 0.368 to 1.163, p<0.001). Formal diagnosis*sex interaction tests supported effect modification by sex for substance use, affective and behavioural syndromes, while evidence for interaction was weaker or absent for psychotic and neurotic disorders (online supplemental table S6).

Alcohol and tobacco use disorders were associated with a metabolite-predicted age exceeding chronological age in both sexes (figure 1D), though alcohol was more strongly associated in females (β=1.008, 95% CI 0.641 to 1.374, p<0.001) and tobacco in males (β=0.535, 95% CI 0.429 to 0.641, p<0.001). Schizophrenia was associated with a MileAge exceeding chronological age in males (β=0.807, 95% CI 0.345 to 1.269, p=0.006) but not in females; however, the sex*diagnosis interaction was not statistically significant after correction (p=0.233). Single-episode depression showed stronger associations in males (β=0.332, 95% CI 0.238 to 0.426, p<0.001) than females (β=0.197, 95% CI 0.127 to 0.268, p<0.001). Associations between stress-related disorders and MileAge delta were statistically significant in males but not in females, although the interaction test did not reach significance after correction (p=0.233). Obsessive-compulsive and eating disorders were associated with a MileAge younger than chronological age in females but not in males; the corresponding sex*diagnosis interactions were not statistically significant after correction. Full results are reported in online supplemental table S7. At the individual-diagnosis level, most diagnosis*sex interaction estimates did not survive multiple-testing correction; however, single-episode depression showed evidence of sex modification (online supplemental table S8).

### Age-stratified analyses

Associations between broad diagnostic groups and MileAge delta were generally stronger in individuals aged <65 years than in those aged ≥65 years (figure 1E). An association between affective disorders and metabolomic ageing was observed in those aged <65 years (β=0.255, 95% CI 0.194 to 0.316, p<0.001) but not in the older age group (β=−0.051, 95% CI −0.183 to 0.081, p=0.896; online supplemental table S9). Psychosis showed a similar pattern, with a strong association observed in the younger age group (β=0.630, 95% CI 0.296 to 0.965, p<0.001) but not in those aged 65 or older (β=−0.147, 95% CI −0.907 to 0.613, p=0.920), though the latter included only 80 cases. Substance use disorders were associated with a metabolite-predicted age exceeding chronological age in both age groups, with comparable effect sizes (younger: β=0.374, 95% CI 0.293 to 0.455, p<0.001; older: β=0.386, 95% CI 0.229 to 0.543, p<0.001). Behavioural syndromes were associated with a younger MileAge in those below age 65 but not in those aged 65 years and older. Formal interaction tests supported effect modification by age group for the any-disorder phenotype and affective disorders, with nominally significant interactions for psychosis and behavioural syndromes (online supplemental table S10).

At the individual-diagnosis level, associations for alcohol use disorder, schizophrenia, single-episode and recurrent depression, stress-related disorders, eating disorders, puerperal psychosis and personality disorders reached statistical significance only in the younger age group (figure 1F; online supplemental table S11). However, except for single-episode depression, the corresponding diagnosis*age interaction tests did not reach statistical significance after correction. Tobacco use disorder was associated with a metabolite-predicted age exceeding chronological age in both age groups, with very similar effect sizes (younger: β=0.358, 95% CI 0.271 to 0.445, p<0.001; older: β=0.406, 95% CI 0.241 to 0.571, p<0.001), consistent with the absence of a significant interaction (online supplemental table S12). Single-episode depression showed stronger associations in those aged below 65 years (β=0.259, 95% CI 0.197 to 0.322, p<0.001) than in the older individuals (β=−0.028, 95% CI −0.164 to 0.109, p=0.906), supported by a formal interaction test (β_interaction_=−0.264, 95% CI −0.423 to −0.105, p=0.029).

### Polygenic score analysis

**Figure 2:**
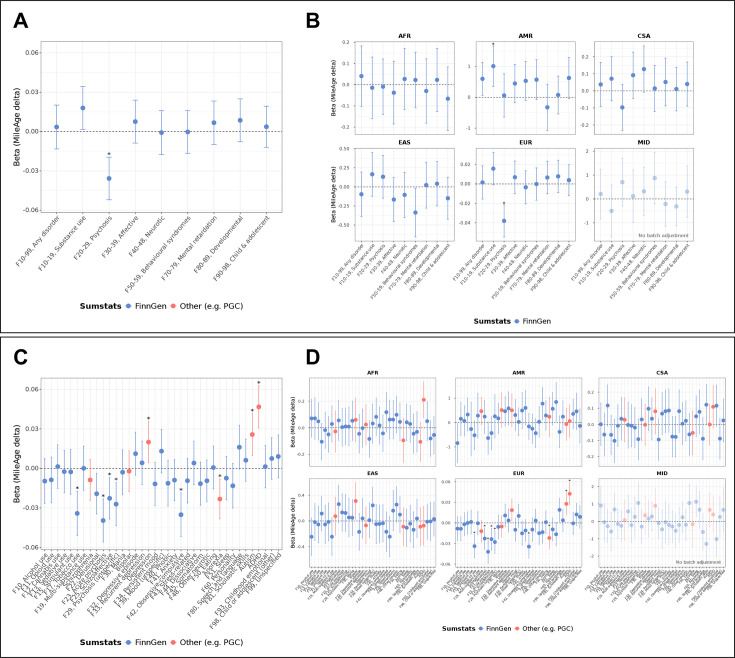
MileAge delta and polygenic scores for mental/behavioural disorders. (A) Associations between polygenic scores for groups of mental/behavioural disorders and MileAge delta across ancestries. (B) Ancestry-specific associations between polygenic scores for groups of mental/behavioural disorders and MileAge delta. (C) Associations between polygenic scores for individual mental/behavioural disorders and MileAge delta across ancestries. (D) Ancestry-specific associations between polygenic scores for individual mental/behavioural disorders and MileAge delta. (A–D) The estimates shown are ordinary least squares regression beta coefficients and 95% CIs. Models were adjusted for chronological age, sex, assessment centre, batch number, the first six genetic principal components and fasting time. Asterisks indicate statistical significance after correcting p-values for multiple testing using the Benjamini-Hochberg procedure (separately for groups of diagnoses and individual diagnoses, and separately within each population). (A and C) N=219 494. (B and D) N=3426 (AFR); N=278 (AMR); N=3916 (CSA); N=1012 (EAS); N=210,755 (EUR); N=94 (MID). ADHD, attention-deficit/hyperactivity disorder; AFR, African; AMR, admixed American; CSA, Central and South Asian; EAS, East Asian; EUR, European; MID, Middle Eastern; PGC, Psychiatric Genomics Consortium.

We next tested whether genetic liability to mental disorders was associated with metabolomic ageing. Polygenic scores for psychosis were associated with a metabolite-predicted age younger than chronological age (figure 2A; online supplemental table S13). Ancestry-stratified analyses suggested that the association between higher genetic loadings for psychosis and a younger metabolite-predicted age was statistically significant only in Europeans (figure 2B; online supplemental table S14). Polygenic scores for substance use disorders were associated with a MileAge exceeding chronological age in admixed Americans (mixed Indigenous American, European and African ancestry).

Polygenic scores for major depression, autism and attention-deficit/hyperactivity disorder (ADHD) were associated with a MileAge exceeding chronological age (β range=0.020–0.047; online supplemental table S15). In contrast, higher genetic liability to tobacco use disorder, acute psychosis, schizoaffective disorder, unspecific psychosis, obsessive-compulsive disorder and anorexia nervosa was associated with a MileAge younger than chronological age (β range=−0.023 to −0.040; figure 2C). After multiple testing corrections, these associations were statistically significant only in Europeans (figure 2D). Nominal associations were observed in other ancestry groups (online supplemental table S16). For example, polygenic scores for bipolar disorder (East Asian), other neurotic disorders (admixed American) and ADHD (African) were associated with a metabolite-predicted age exceeding chronological age. Polygenic scores for alcohol use disorder (admixed American), developmental disorders of scholastic skills and childhood emotional disorders (Middle Eastern) were associated with a younger MileAge.

### Sensitivity analysis

Excluding self-reported physician diagnoses (applied to 12 of 45 ICD-10 codes) yielded comparable results (online supplemental table S17). Sample sizes declined between 15.4% (alcohol use disorder) and 77.2% (bipolar disorder). Some differences emerged in both directions: schizophrenia and eating disorders were no longer statistically significantly associated with MileAge delta, whereas obsessive-compulsive and anxiety disorders reached statistical significance in both models.

In the subset with primary care data, most estimates were attenuated (online supplemental table S18 and S19). Sample sizes were reduced for 38 of 45 ICD-10 codes. Some associations, such as for eating disorders, no longer reached statistical significance after full adjustment.

Adjusting for smoking produced negligible changes relative to Model 2 (online supplemental table S20 and S21). BMI adjustment, with or without smoking, attenuated some associations, including eating disorders, schizophrenia and stress-related disorders, which were no longer statistically significant after BMI adjustment, while strengthening others, including opioid use and puerperal psychosis. The overall pattern of results was otherwise largely unchanged.

### Psychiatric comorbidity analyses

Results from analyses restricted to mutually exclusive diagnoses were broadly consistent with the primary findings, although effect sizes were generally attenuated and CIs were wider, reflecting reduced sample sizes (online supplemental table S22 and S23). Associations for substance use and affective disorders remained statistically significant, whereas estimates for many other disorders were not statistically significant.

In analyses examining overall psychiatric comorbidity, increasing comorbidity burden was associated with progressively higher metabolomic age relative to chronological age (online supplemental table S12). Compared with individuals without any mental disorder, those with diagnoses from one, two, or three or more ICD-10 diagnostic groups showed increasingly higher MileAge delta values, with the strongest associations observed among individuals with three or more diagnostic groups (online supplemental table S24). Similar patterns were observed when comorbidity was defined as the number of individual ICD-10 diagnoses, with the largest differences in metabolomic ageing seen among individuals with four or more diagnoses.

## Discussion

Mental disorders are associated with a greater age-related disease burden and reduced life expectancy. Accelerated biological ageing has been proposed as one mechanism contributing to these disparities, yet evidence linking mental disorders to molecular ageing markers remains limited, particularly at population scale and across diagnostic groups. In this study of over 225 000 individuals, we show that metabolomic ageing in mental disorders is heterogeneous. Substance use, psychotic, affective and neurotic disorders were associated with a metabolite-predicted age (MileAge) exceeding chronological age. In contrast, behavioural syndromes such as eating disorders were associated with a MileAge younger than chronological age, with this pattern most evident in females in stratified analyses. Age-stratified analyses revealed that most associations were pronounced in individuals aged <65 years and attenuated or absent in those aged ≥65 years. In polygenic score analyses, higher genetic liability to major depression, autism and ADHD was associated with a metabolite-predicted age exceeding chronological age, whereas higher genetic liability to tobacco use disorder, psychosis, obsessive-compulsive disorder and anorexia nervosa was associated with a MileAge younger than chronological age. Ancestry-stratified analyses showed that substance use disorder polygenic scores were associated with a metabolite-predicted age exceeding chronological age in individuals of mixed Indigenous American, European and African ancestry.

Taken together, these findings indicate that altered metabolomic ageing in mental disorders does not follow a uniform direction but instead reflects disorder-specific biological profiles. An older metabolomic age likely captures cumulative metabolic strain, inflammation or exposure to adverse health behaviours, whereas a younger metabolomic age does not necessarily indicate a healthier state but may reflect atypical or constrained metabolic processes. From this perspective, opposing directions of metabolomic ageing across disorders may point to fundamentally different underlying pathophysiological mechanisms rather than a single ageing-related pathway shared across mental disorders. It is worth noting that ageing clocks based on biomarkers such as metabolites can be influenced by condition-specific metabolic perturbations, in addition to reflecting biological ageing processes. For example, starvation-related lipid suppression in anorexia nervosa may contribute to a younger MileAge, while smoking-related lipid and inflammatory changes or antipsychotic-induced metabolic effects may contribute to an older MileAge. These considerations add nuance to disorder-specific patterns, particularly for our schizophrenia finding, given the known metabolic side effects of antipsychotic medications.

Most previous studies of biological ageing in mental disorders have focused on mood and stress/anxiety-related disorders, reporting mixed findings across ageing indicators.^13 24 25^ Few have investigated metabolomic ageing. A UK-based study of police officers found that depression, anxiety and PTSD were associated with an older metabolomic age,^13^ which partially aligns with our results. However, a Dutch study reported no association between depression and metabolomic ageing.^14^ Our observation that depression was associated with altered metabolomic profiles aligns with a Mendelian randomisation study showing that depression may causally affect certain metabolites.^26^ Interestingly, metabolomic profiles were similar in lifetime and recurrent depression, aligning with our observation that associations with MileAge delta were similar for single-episode and recurrent depression. That said, a separate Mendelian randomisation study identified metabolites as potential causal contributors to depression rather than the reverse.^27^

Substance use disorders were also associated with a metabolite-predicted age exceeding chronological age, consistent with prior evidence of older epigenetic age in alcohol use disorder^28^ and higher brain-predicted age among those with tobacco and alcohol use.^29^ Smoking is known to alter circulating metabolites,^30^ which may partly explain the observation of MileAge exceeding chronological age in tobacco use disorder. Fewer cases were identified for substances other than alcohol and tobacco, highlighting the need for further research.

Interestingly, both obsessive-compulsive and eating disorders were associated with a metabolite-predicted age younger than chronological age in females, consistent with the high genetic correlation between these disorders.^31^ As noted above, this may reflect a more youthful metabolic profile, but it could also indicate constrained metabolic states rather than a protective phenotype. As such, a younger metabolomic age should not be interpreted as inherently beneficial.

Our finding that schizophrenia was associated with a MileAge exceeding chronological age in males aligns with a recent lipidomic study of prefrontal cortex tissue in schizophrenia and autism.^32^ The observation that puerperal psychosis showed metabolomic ageing patterns distinct from other psychotic disorders supports the notion that shared diagnostic labels may mask biologically heterogeneous processes, particularly in conditions strongly influenced by acute hormonal and physiological changes.

Psychiatric comorbidity represents an important methodological challenge in studies of mental disorders, as overlapping diagnoses may inflate or obscure disorder-specific associations. By conducting sensitivity analyses restricted to mutually exclusive diagnoses, we showed that our key findings were not driven solely by diagnostic overlap, although estimates for less prevalent disorders became less precise. Importantly, analyses of overall psychiatric comorbidity revealed a clear relationship between cumulative psychiatric burden and metabolomic ageing. This pattern suggests that biological ageing differences may reflect shared or additive effects of multiple mental disorders, potentially mediated by chronic stress exposure, health behaviours, medication use or systemic metabolic dysregulation. These findings highlight the importance of considering comorbidity when interpreting disorder-specific associations.

The magnitude of observed associations was generally small to moderate, typically corresponding to differences of several months to approximately 1 year in metabolomic age. For example, substance use and psychotic disorders were associated with metabolomic ages approximately 0.4–0.6 years older than chronological age, while eating disorders were associated with metabolomic ages about half a year younger than expected. Although these differences may appear small, shifts of this magnitude may translate into meaningful differences in mortality risk and age-related health outcomes. At the population level, such differences may therefore contribute to the substantially reduced life expectancy observed in mental disorders.

A novel contribution of this study lies in the analysis of sex differences. Associations were generally stronger in males, although, for instance, females showed nominally stronger associations for alcohol use disorder. Psychosis and stress-related disorders were associated with a metabolite-predicted age exceeding chronological age in males but not in females, while obsessive-compulsive and eating disorders were associated with a MileAge younger than chronological age in females but not in males. While these differences may partly reflect sample size imbalances (eg, 342 females vs 22 males with eating disorders), the consistency of directionally distinct patterns across several disorders suggests that at least some differences are biologically meaningful rather than statistical artefacts. For example, they could indicate sex-specific biological pathways, including hormonal influences or differential health behaviours (eg, higher smoking rates in men with schizophrenia). Our findings underscore the importance of considering sex in studies of biological ageing in mental disorders.

In age-stratified analyses, most associations were attenuated or absent in individuals aged ≥65 years, with formal interaction tests supporting effect modification for affective disorders and single-episode depression; tobacco use disorder was a notable exception, showing comparable effect sizes across age groups. This pattern may partly reflect survivor bias and reduced case numbers in the older age group or could suggest that associations between mental disorders and metabolomic ageing are most pronounced in mid-life.

We also explored associations between metabolomic ageing and genetic liability to mental disorders to help distinguish whether observed differences in biological ageing primarily reflect downstream consequences of illness and associated behaviours or whether inherited risk is also linked to ageing-related processes. Higher polygenic scores for major depression, autism and ADHD were associated with a metabolite-predicted age exceeding chronological age. In contrast, higher polygenic scores for tobacco use disorder, psychosis, obsessive-compulsive disorder and anorexia nervosa were associated with a MileAge younger than chronological age. These associations were modest, corresponding to a maximum difference of ~2.5 weeks per SD increase in polygenic score. Although current polygenic scores explain only a small proportion of the variance in their target traits, these findings suggest that differences in metabolomic ageing are not driven primarily by common genetic variants linked to these disorders. Importantly, concordance between associations for clinical diagnoses and genetic liability was not assumed a priori, as these analyses address distinct but complementary questions. We identified diverging results between diagnosis and polygenic scores: for example, while psychosis diagnoses were associated with a MileAge exceeding chronological age, higher polygenic scores for psychosis were associated with a metabolite-predicted age younger than chronological age. These discrepancies may reflect non-genetic influences on biological ageing. While most associations survived multiple testing correction only in individuals of European ancestry, substance use disorder polygenic scores were associated with a metabolite-predicted age exceeding chronological age in admixed Americans. We also identified nominal associations in other populations: for example, bipolar disorder polygenic scores were associated with a MileAge exceeding chronological age in East Asians. Given that our polygenic scores were based on European GWAS summary statistics, these findings should be interpreted cautiously.

Certain limitations must be acknowledged. First, differences in metabolomic ageing may not reflect a causal effect of mental disorders on biological ageing. Although we restricted analyses to diagnoses occurring prior to biomarker assessment, there is emerging evidence that a biological age exceeding chronological age may also precede and predict the onset of mental disorders.^33^,^35^ While this raises the possibility of a bidirectional relationship, most participants in our study were aged 40–69 years at baseline, with the age of onset for many diagnoses well preceding the assessment of MileAge. Of note, duration of illness may impact biological ageing, particularly for chronic conditions such as tobacco use disorder or for episodic disorders such as recurrent depression. Future work should examine this further. Second, although we adjusted for potential confounders, we cannot exclude the possibility of residual confounding. For example, smoking and BMI were not adjusted for in the primary models because they plausibly lie on the causal pathway. In sensitivity analyses, BMI adjustment altered estimates in both directions, consistent with heterogeneous relationships between adiposity and mental disorders; future studies explicitly modelling smoking and BMI as mediators may help disentangle these pathways. More broadly, somatic comorbidities (eg, type 2 diabetes and cardiovascular disease), psychiatric medication use and residual structure related to sampling conditions (eg, assessment centre, seasonality and time of day) may each contribute to variation in MileAge delta independently of mental disorders, though fasting time was included as a covariate and UKB protocols were standardised across centres. Unmeasured genetic confounding remains a limitation. Third, some diagnostic misclassification is possible. Individuals with mental disorders were identified mostly from linked health records. Health record-based ascertainment may miss milder, untreated or undiagnosed cases, whereas self-reported physician diagnoses may be subject to recall error. Sensitivity analyses largely confirmed our findings, though some associations attenuated and were no longer statistically significant with more restricted case ascertainment. Findings for diagnoses with few cases should be interpreted cautiously. Some categories were particularly under-represented (eg, ADHD and certain substance use disorders), possibly reflecting the cohort’s middle-aged profile and healthy volunteer bias. Moreover, in highly imbalanced case-control designs, statistical power is determined primarily by case numbers rather than controls, leading to imprecise estimates and an elevated risk of false-negative findings. As such, null results should not be interpreted as evidence of absence. We also note that in analyses examining psychiatric comorbidity, comorbidity counts may underestimate lifetime diagnostic complexity (eg, the timing and sequencing of diagnoses). Fourth, given the well-documented healthy volunteer bias in the UKB and the moderate prediction accuracy of MileAge (mean absolute error=5.35 years), observed differences in metabolomic ageing represent conservative estimates, as the most clinically severe cases are not well represented in the data and measurement error in the outcome would attenuate associations towards the null. UKB participants are more socioeconomically advantaged and healthier than the general population, which may limit generalisability. Future studies should consider weighting approaches. Moreover, replication in independent cohorts with comparable metabolomic profiling and psychiatric phenotyping will be an important next step to confirm the robustness of these findings. Finally, most GWAS summary statistics used for polygenic scoring were obtained from the FinnGen study, which comprises over 500 000 Finnish participants.^36^ The portability of these polygenic scores to other ancestry groups is uncertain. Although we conducted ancestry-stratified analyses, reduced power and limited transferability of polygenic scores outside European populations remain important constraints.

## Conclusion

In conclusion, we showed that metabolomic ageing in mental and behavioural disorders is heterogeneous. Substance use, psychotic, affective and neurotic disorders were associated with a metabolite-predicted age exceeding chronological age, whereas obsessive-compulsive disorder and behavioural syndromes such as eating disorders were associated with a metabolite-predicted age younger than chronological age. Several associations differed by sex and age group: psychosis and stress-related disorders were associated with a metabolite-predicted age exceeding chronological age in males but not in females, while obsessive-compulsive and eating disorders were associated with a metabolite-predicted age younger than chronological age in females but not in males. Age-stratified analyses revealed that associations for several disorders, including affective disorders and single-episode depression, were observed primarily in individuals aged <65 years. However, sex and age-stratified findings should be considered exploratory for most disorders, given sample size imbalances and limited support from formal interaction tests. The divergence between genetic liability and clinical manifestations in some disorders suggests that environmental exposures and health behaviours may shape biological ageing in mental disorders.

## Supplementary material

10.1136/bmjment-2025-302181 online supplemental figure 1

## Data Availability

Data may be obtained from a third party and are not publicly available.
