# Supplementary material for: Metabolomic ageing across mental and behavioural disorders
Source: BMJ Ment Health. 2026 Jun 23;29(1):e302181. doi: 10.1136/bmjment-2025-302181 (PMC13288872; doi:10.1136/bmjment-2025-302181)
Supplement: online supplemental figure 1 [file bmjment-29-1-s001.pdf]

# Supplementary material

This material accompanies the article

# Metabolomic ageing across mental and behavioural disorders

**Authors:** Julian Mutz, Lachlan Gilchrist, Andrea G. Allegrini, Sandra Sanchez Roige and Cathryn M Lewis

**Table of contents:**

1. Supplementary methods ........ 2
2. GWAS summary statistics ........ 3
3. Directed acyclic graph ........ 5
4. Study flowchart ........ 6
5. Time between first diagnosis and baseline, groups of two-digit ICD-10 codes ........ 7
6. Time between first diagnosis and baseline, individual two-digit ICD-10 codes ........ 9
7. MileAge delta and individual two-digit ICD-10 codes ........ 18
8. MileAge delta and groups of two-digit ICD-10 codes; sex-stratified ........ 19
9. MileAge delta and groups of two-digit ICD-10 codes; sex-interactions ........ 20
10. MileAge delta and individual two-digit ICD-10 codes; sex-stratified ........ 21
11. MileAge delta and individual two-digit ICD-10 codes; sex-interactions ........ 23
12. MileAge delta and groups of two-digit ICD-10 codes; age-stratified ........ 24
13. MileAge delta and groups of two-digit ICD-10 codes; age-interactions ........ 25
14. MileAge delta and individual two-digit ICD-10 codes; age-stratified ........ 26
15. MileAge delta and individual two-digit ICD-10 codes; age-interactions ........ 28
16. MileAge delta and polygenic scores for groups of disorders ........ 29
17. MileAge delta and polygenic scores for individual disorders ........ 31
18. MileAge delta and individual two-digit ICD-10 codes; excluding self-report ........ 36
19. MileAge delta and groups of two-digit ICD-10 codes; primary care linkage ........ 37
20. MileAge delta and individual two-digit ICD-10 codes; primary care linkage ........ 38
21. MileAge delta and groups of two-digit ICD-10 codes; smoking and BMI adjustment ..... 39
22. MileAge delta and individual two-digit ICD-10 codes; smoking and BMI adjustment .... 40
23. MileAge delta and groups of two-digit ICD-10 codes; mutually exclusive ........ 42
24. MileAge delta and individual two-digit ICD-10 codes; mutually exclusive ........ 43
25. MileAge delta and psychiatric comorbidity scores ........ 44

# 1. Supplementary methods

**Imputed genotype data**

Details on genotype calling, quality control and imputation performed centrally by the UK Biobank have been reported elsewhere (Bycroft et al., 2018). Individuals were genotyped using the UK BiLEVE Axiom ($N = 49{,}950$) and UK Biobank Axiom ($N = 438{,}427$) arrays. The data were imputed using the Haplotype Reference Consortium (HRC) and the combined UK10K and 1000 Genomes Project phase 3 reference panels, with imputed data available for approximately 93,095,623 autosomal SNPs. These were filtered using a minor allele frequency (MAF) threshold of ≥ 1% and INFO score of ≥ 0.4.

**Genotype quality control**

Quality control steps were applied using the GenoPred pipeline (v2.2.11) (Pain, Al-Chalabi, & Lewis, 2024).

GWAS summary statistics: Variants were excluded if they were not identified in the reference dataset, were strand ambiguous, had an INFO score < 0.9 (if available), had out-of-bound *p*-values ($0 < P \leq 1$), had duplicate SNP IDs, had a sample size > 3SD from the median (if available) or had a standard error of zero. MAF thresholds were set at 0.01 for the GWAS summary statistics and the reference dataset, with a MAF difference threshold of 0.2.

Individual-level genotype data: Variants absent from the reference data and duplicate variants were removed. Variants with mismatched RSIDs and variants that were strand-flipped were corrected. Strand-ambiguous variants were excluded. Reference variants absent from the UK Biobank data were inserted as missing to allow reference allele frequency-based imputation during downstream polygenic scoring. Relatedness was estimated using the KING estimator in PLINK v1.9, with unrelated individuals defined using a threshold of $r > 0.044$ (equivalent to removing third-degree relatives and closer).

**Supplementary references**

Bycroft, C., Freeman, C., Petkova, D., Band, G., Elliott, L. T., Sharp, K., . . . O'Connell, J. (2018). The UK Biobank resource with deep phenotyping and genomic data. *Nature, 562*(7726), 203-209. doi:https://doi.org/10.1038/s41586-018-0579-z

Pain, O., Al-Chalabi, A., & Lewis, C. M. (2024). The GenoPred pipeline: a comprehensive and scalable pipeline for polygenic scoring. *Bioinformatics, 40*(10), btae551. doi:10.1093/bioinformatics/btae551

## 2. GWAS summary statistics

**Table S1.** Genome-wide association study (GWAS) summary statistics

| Code | $N_{Cases}$ | $N_{Controls}$ | $N_{Total}$ | $N_{Effective}$ | $Prop._{Cases}$ | Prevalence[1] | PMID |
|---|---|---|---|---|---|---|---|
| Groups of ICD-10 codes | | | | | | | |
| F10–F99 | 138044 | 362304 | 500348 | 399833 | 0.27589598 | 0.27589598 | 36653562 |
| F10–19 | 30806 | 362304 | 393110 | 113568 | 0.07836483 | 0.07836483 | 36653562 |
| F20–29 | 15498 | 362304 | 377802 | 59449 | 0.04102149 | 0.04102149 | 36653562 |
| F30–39 | 65517 | 434831 | 500348 | 227752 | 0.13094286 | 0.13094286 | 36653562 |
| F40–48 | 56552 | 362304 | 418856 | 195666 | 0.13501538 | 0.13501538 | 36653562 |
| F50–59 | 15447 | 484901 | 500348 | 59880 | 0.03087251 | 0.03087251 | 36653562 |
| F60–69 | | | | *N* insufficient | | | |
| F70–79 | 1429 | 362234 | 363663 | 5694 | 0.00392946 | 0.00392946 | 36653562 |
| F80–89 | 5519 | 494829 | 500348 | 21832 | 0.01103032 | 0.01103032 | 36653562 |
| F90–98 | 9640 | 490708 | 500348 | 37817 | 0.01926659 | 0.01926659 | 36653562 |
| F99 | 3449 | 479456 | 482905 | 13697 | 0.00714219 | 0.00714219 | 36653562 |
| Individual ICD-10 codes[2] | | | | | | | |
| F10 | 20597 | 479751 | 500348 | 78996 | 0.04116535 | 0.0283640 | 36653562 |
| F11 | 1444 | 491508 | 492952 | 5759 | 0.00292929 | 0.0038920 | 36653562 |
| F12 | 1168 | 491508 | 492676 | 4661 | 0.00237073 | 0.0052201 | 36653562 |
| F13 | 2668 | 491508 | 494176 | 10614 | 0.00539889 | 0.0019398 | 36653562 |
| F15 | 1077 | 491508 | 492585 | 4299 | 0.00218642 | 0.0008272 | 36653562 |
| F16 | | | | *N* insufficient | | | |
| F17 | 3200 | 495423 | 498623 | 12718 | 0.00641767 | 0.0672100 | 36653562 |
| F19 | 2866 | 491508 | 494374 | 11398 | 0.00579723 | 0.0047489 | 36653562 |
| Schizophrenia | 76755 | 243649 | 320404 | 233471 | 0.23955690 | 0.01 | 35396580 |
| F22 | 2953 | 484776 | 487729 | 11740 | 0.00605459 | 0.0021637 | 36653562 |
| F23 | 5003 | 484776 | 489779 | 19808 | 0.01021481 | 0.0010761 | 36653562 |
| F25 | 3095 | 484776 | 487871 | 12301 | 0.00634389 | 0.0010109 | 36653562 |
| F29 | 8976 | 484776 | 493752 | 35251 | 0.01817917 | 0.01817917 | 36653562 |
| F30 | 1157 | 434831 | 435988 | 4616 | 0.00265374 | 0.0010171 | 36653562 |
| Bipolar disorder | 20352 | 31358 | 51710 | 49367 | 0.39357960 | 0.02 | 31043756 |
| F32 | 59333 | 434831 | 494164 | 208836 | 0.12006743 | 0.1155600 | 36653562 |
| F33 | 26094 | 254393 | 280487 | 94666 | 0.09303105 | 0.0112840 | 36653562 |
| Major depression | 310128 | 1035355 | 1345483 | 829249 | 0.23049570 | 0.12 | 39814019‡ |
| F34 | 9001 | 434831 | 443832 | 35274 | 0.02028020 | 0.0015478 | 36653562 |
| F38 | | | | *N* insufficient | | | |
| F39 | 3063 | 434831 | 437894 | 12166 | 0.00699484 | 0.00699484 | 36653562 |
| F40 | 6248 | 444414 | 450662 | 24646 | 0.01386405 | 0.0081370 | 36653562 |
| F41 | 35875 | 444414 | 480289 | 132781 | 0.07469461 | 0.0900500 | 36653562 |
| F42 | 2813 | 444414 | 447227 | 11181 | 0.00628987 | 0.0043330 | 36653562 |
| F43 | 18806 | 444414 | 463220 | 72170 | 0.04059842 | 0.0592720 | 36653562 |
| F44 | 1525 | 444414 | 445939 | 6079 | 0.00341975 | 0.0011276 | 36653562 |
| F45 | 7148 | 444414 | 451562 | 28139 | 0.01582950 | 0.0033942 | 36653562 |
| F48 | 1914 | 444414 | 446328 | 7623 | 0.00428833 | 0.0031640 | 36653562 |
| F50 | 3923 | 362304 | 366227 | 15524 | 0.01071194 | 0.0059592 | 36653562 |
| Anorexia nervosa | 16224 | 52460 | 68684 | 49566 | 0.23621220 | 0.01 | 31308545‡ |
| F51 | 8336 | 362304 | 370640 | 32594 | 0.02249083 | 0.0028453 | 36653562 |
| F52 | | | | *N* insufficient | | | |
| F53 | | | | *N* insufficient | | | |
| F60 | 13045 | 362300 | 375345 | 50367 | 0.03475469 | 0.0058590 | 36653562 |
| F63 | | | | *N* insufficient | | | |
| F66 | | | | *N* insufficient | | | |
| F69 | | | | *N* insufficient | | | |

| | | | | | | | |
|---|---|---|---|---|---|---|---|
| F80 | 2362 | 494829 | 497191 | 9403 | 0.00475069 | 0.0042170 | 36653562 |
| F81 | 1719 | 494829 | 496548 | 6852 | 0.00346190 | 0.0048760 | 36653562 |
| F84 | | | | *N* insufficient | | | |
| Autism | 22458 | 29386 | 51844 | 50918 | 0.43318420 | 0.01 | 32747698 |
| ADHD | 38691 | 186843 | 225534 | 128213 | 0.17155280 | 0.05 | 36702997 |
| F91 | | | | *N* insufficient | | | |
| F93 | 2230 | 490708 | 492938 | 8880 | 0.00452390 | 0.0008979 | 36653562 |
| F95 | | | | *N* insufficient | | | |
| F98 | 2097 | 362304 | 364401 | 8340 | 0.00575465 | 0.00117680 | 36653562 |
| F99 | 3449 | 479456 | 482905 | 13697 | 0.00714219 | 0.00714219 | 36653562 |

*Note:* ADHD = Attention-deficit/hyperactivity disorder; PMID = PubMed reference number. Summary statistics were limited to those with a minimum of 1000 cases and excluded UK Biobank participants, ensuring no overlap between discovery and target samples. [1] Most prevalence estimates were obtained from https://www.prevalenceuk.com/ and correspond to lifetime prevalence; alternatively, we report estimates reported elsewhere or the proportion of cases (printed in grey). [2] Mapping to two-digits ICD-10 codes may differ for consortia summary statistics (listed without ICD-10 code). [†] Excluding UK Biobank and 23andMe. [‡] Excluding UK Biobank.

## 3. Directed acyclic graph

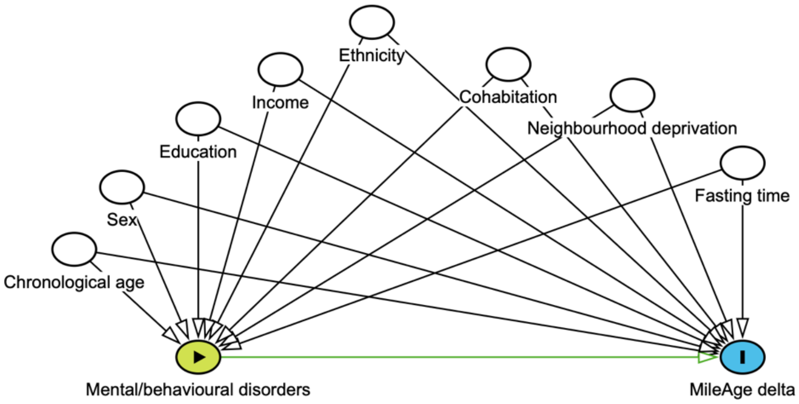


**Figure S1.** Directed acyclic graph (DAG) showing relationships between the exposure (mental/behavioural disorders), outcome (MileAge delta) and select confounders (chronological age, sex, highest educational/professional qualification, gross annual household income, ethnicity, cohabitation with spouse/partner, Townsend deprivation index and fasting time).

## 4. Study flowchart

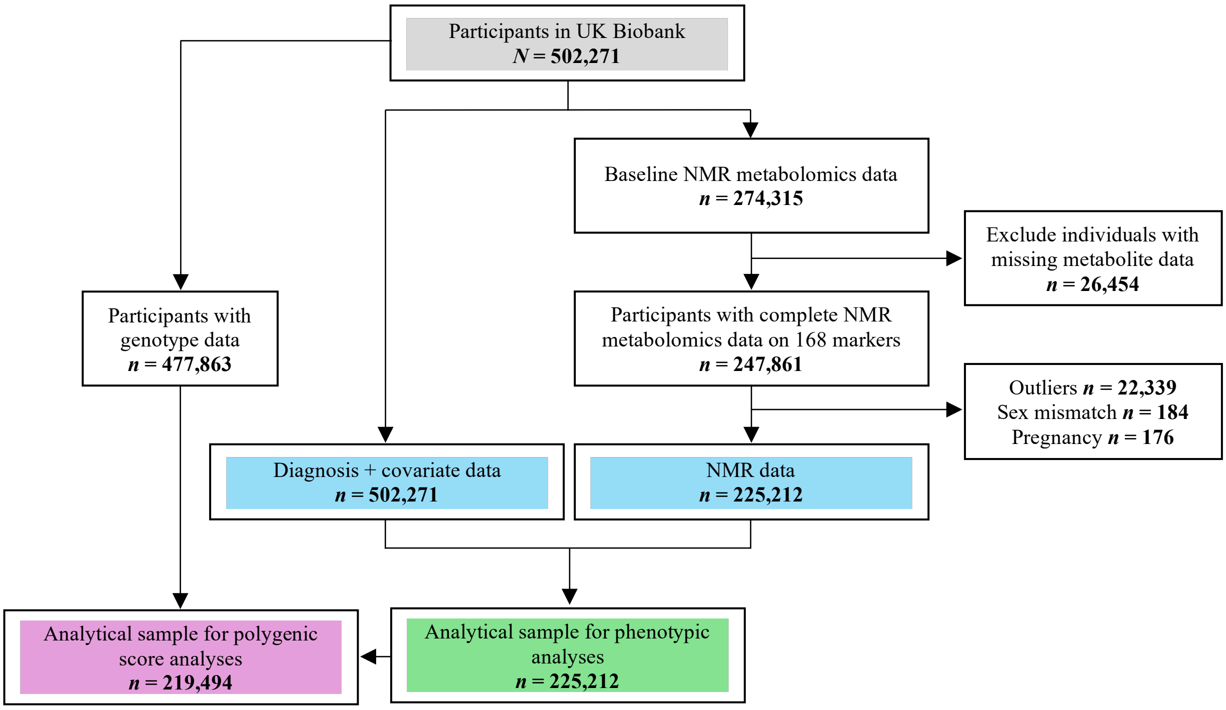


**Figure S2.** Study sample flowchart. Outliers were defined as metabolite values 4×IQR above or below the median. NMR = nuclear magnetic resonance; IQR = interquartile range.

## 5. Time between first diagnosis and baseline, groups of two-digit ICD-10 codes

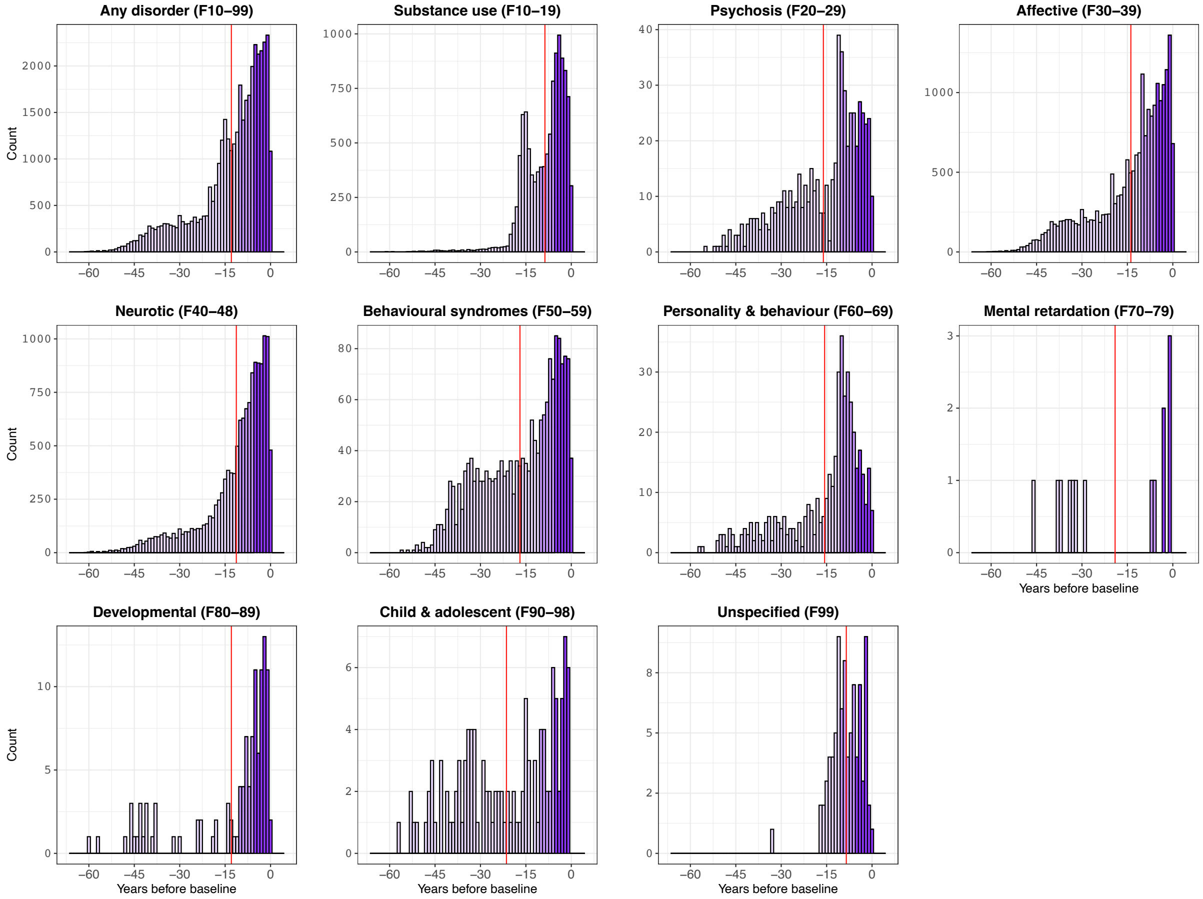


**Figure S3.** Differences in years between the first recorded diagnosis of mental/behavioural disorders and the baseline assessment date, shown for groups of two-digit ICD-10 codes. ICD-10 = International Classification of Diseases, 10th Revision. Vertical red lines show the mean difference. Sample sizes reported in Table 2.

**Table S2.** Time between first diagnosis and baseline, groups of ICD-10 codes

| ICD-10 | Mean | SD | Median | IQR |
|---|---|---|---|---|
| F10-99, Any disorder | -12.94 | 11.41 | -9.71 | 12.97 |
| F10-19, Substance use | -8.71 | 6.78 | -6.77 | 10.28 |
| F20-29, Psychosis | -16.07 | 12.48 | -11.29 | 18.40 |
| F30-39, Affective | -13.85 | 12.18 | -9.96 | 15.67 |
| F40-48, Neurotic | -11.30 | 10.80 | -7.86 | 11.45 |
| F50-59, Behavioural syndromes | -16.95 | 12.87 | -13.59 | 21.79 |
| F60-69, Personality & behaviour | -15.67 | 12.80 | -10.80 | 14.16 |
| F70-79, Mental retardation | -19.06 | 17.39 | -17.71 | 31.13 |
| F80-89, Developmental | -12.94 | 15.32 | -6.18 | 11.55 |
| F90-98, Child & adolescent | -21.41 | 15.71 | -18.95 | 27.90 |
| F99, Unspecified | -8.50 | 5.12 | -8.57 | 6.84 |

*Note:* ICD-10 = International Classification of Diseases; SD = standard deviation; IQR = interquartile range. Sample sizes reported in Table 2.

# 6. Time between first diagnosis and baseline, individual two-digit ICD-10 codes

**Table S3.** Time between first diagnosis and baseline, individual ICD-10 codes

| ICD-10 | Mean | SD | Median | IQR |
|---|---|---|---|---|
| F10, Alcohol use | -7.99 | 8.43 | -5.63 | 7.51 |
| F11, Opioid use | -9.22 | 9.85 | -5.93 | 10.05 |
| F12, Cannabis use | -6.49 | 6.90 | -4.33 | 6.48 |
| F13, Sedative use | -7.94 | 7.62 | -4.90 | 8.34 |
| F14, Cocaine use | -6.61 | 4.88 | -6.54 | 3.32 |
| F15, Stimulant use | -7.78 | 8.33 | -5.52 | 6.06 |
| F16, Hallucinogen use | -8.74 | 11.65 | -3.23 | 7.85 |
| F17, Tobacco use | -8.60 | 6.31 | -6.76 | 10.37 |
| F19, Multi-substance use | -16.76 | 12.26 | -14.73 | 18.80 |
| F20, Schizophrenia | -18.33 | 12.49 | -16.28 | 18.75 |
| F22, Delusional | -8.35 | 6.72 | -6.56 | 6.46 |
| F23, Acute psychosis | -10.46 | 11.81 | -7.14 | 8.05 |
| F25, Schizoaffective | -12.06 | 11.71 | -8.61 | 6.66 |
| F29, Psychosis (unspecific) | -11.57 | 10.46 | -8.34 | 13.43 |
| F30, Mania | -14.43 | 11.32 | -10.05 | 16.91 |
| F31, Bipolar | -17.43 | 12.52 | -15.06 | 19.42 |
| F32, Depressive episode | -13.75 | 12.18 | -9.92 | 15.64 |
| F33, Recurrent depression | -8.90 | 7.91 | -7.17 | 6.64 |
| F34, Persistent mood | -11.89 | 11.27 | -8.36 | 13.24 |
| F38, Mood (other) | -6.79 | 4.83 | -5.74 | 4.66 |
| F39, Mood (unspecific) | -6.11 | 6.47 | -4.36 | 5.54 |
| F40, Phobic | -10.38 | 10.43 | -6.59 | 10.73 |
| F41, Anxiety | -12.31 | 11.69 | -8.33 | 13.34 |
| F42, Obsessive-compulsive | -14.84 | 12.31 | -11.01 | 16.95 |
| F43, Stress-related | -7.87 | 6.95 | -6.02 | 8.21 |
| F44, Dissociative | -13.43 | 12.85 | -8.32 | 12.56 |
| F45, Somatoform | -8.78 | 8.29 | -6.33 | 8.68 |
| F48, Other neurotic | -17.92 | 13.46 | -14.74 | 21.35 |
| F50, Eating | -25.96 | 12.50 | -27.52 | 16.54 |
| F51, Sleep | -9.73 | 7.07 | -8.45 | 10.92 |
| F52, Sexual dysfunction | -7.23 | 6.44 | -5.63 | 6.77 |
| F53, Puerperal psychosis | -23.03 | 11.42 | -22.95 | 18.60 |
| F60, Personality | -14.32 | 11.81 | -10.50 | 11.18 |
| F63, Impulse | -19.65 | 16.77 | -15.02 | 26.21 |
| F66, Sexual development | -11.75 | 6.92 | -9.66 | 7.09 |
| F69, Personality (unspecific) | -28.08 | 16.13 | -31.18 | 27.87 |
| F80, Speech and language | -12.50 | 16.60 | -5.79 | 9.20 |
| F81, Scholastic skills | -14.57 | 15.87 | -6.68 | 20.44 |
| F84, Pervasive developmental | -6.21 | 5.07 | -5.61 | 5.37 |
| F90, ADHD | -17.41 | 18.03 | -7.82 | 21.67 |
| F91, Conduct | -18.78 | 14.37 | -15.19 | 24.66 |
| F93, Childhood emotional | -24.15 | 15.35 | -32.58 | 27.45 |
| F95, Tic | -14.74 | 16.54 | -7.52 | 14.34 |
| F98, Child or adolescent (other) | -26.25 | 16.46 | -27.08 | 28.62 |
| F99, Unspecified | -8.50 | 5.12 | -8.57 | 6.84 |

*Note:* ICD-10 = International Classification of Diseases; SD = standard deviation; IQR = interquartile range; ADHD = attention-deficit/hyperactivity disorder. Sample sizes reported in Table S4.

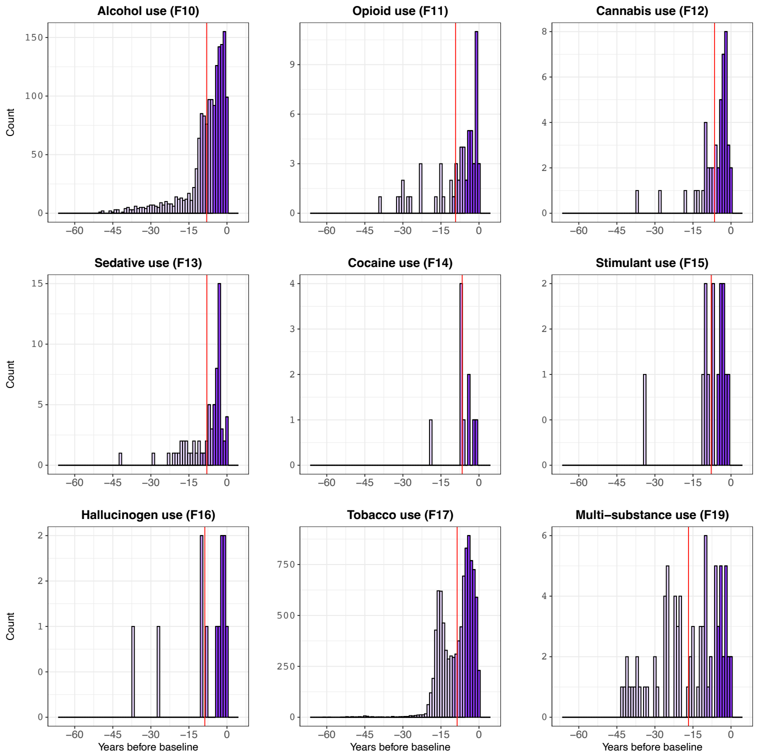


**Figure S4.** Differences in years between the first recorded diagnosis of mental/behavioural disorders and the baseline assessment date, shown for mental and behavioural disorders due to psychoactive substance use (F10–F19). ICD-10 = International Classification of Diseases, 10th Revision. Vertical red lines show the mean difference. Sample sizes reported in Table S4.

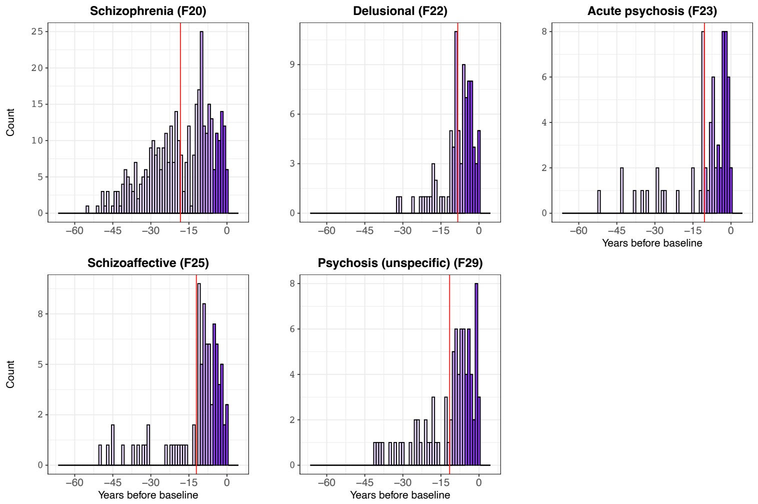


**Figure S5.** Differences in years between the first recorded diagnosis of mental/behavioural disorders and the baseline assessment date, shown for schizophrenia, schizotypal and delusional disorders (F20–F29). ICD-10 = International Classification of Diseases, 10th Revision. Vertical red lines show the mean difference. Sample sizes reported in Table S4.

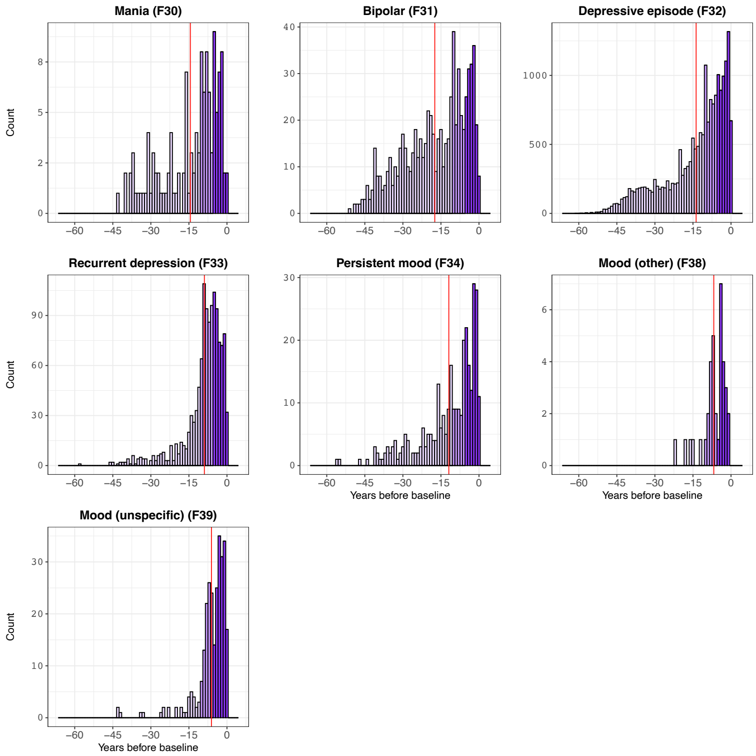


**Figure S6.** Differences in years between the first recorded diagnosis of mental/behavioural disorders and the baseline assessment date, shown for mood [affective] disorders (F30–F39). ICD-10 = International Classification of Diseases, 10th Revision. Vertical red lines show the mean difference. Sample sizes reported in Table S4.

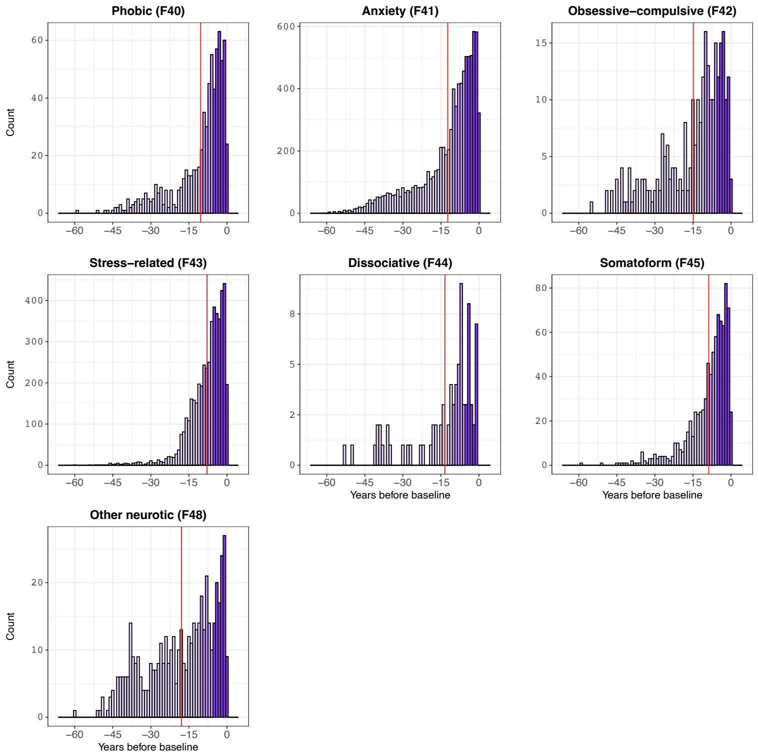


**Figure S7.** Differences in years between the first recorded diagnosis of mental/behavioural disorders and the baseline assessment date, shown for neurotic, stress-related and somatoform disorders (F40–F48). ICD-10 = International Classification of Diseases, 10th Revision. Vertical red lines show the mean difference. Sample sizes reported in Table S4.

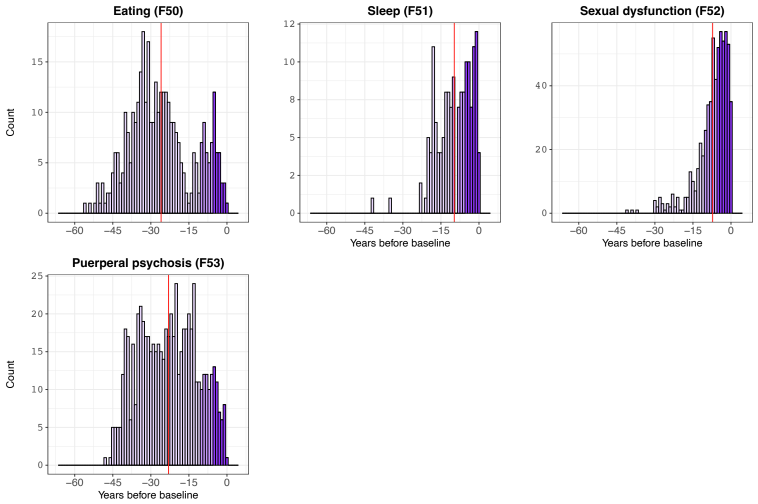


**Figure S8.** Differences in years between the first recorded diagnosis of mental/behavioural disorders and the baseline assessment date, shown for behavioural syndromes associated with physiological disturbances and physical factors (F50–F59). ICD-10 = International Classification of Diseases, 10th Revision. Vertical red lines show the mean difference. Sample sizes reported in Table S4.

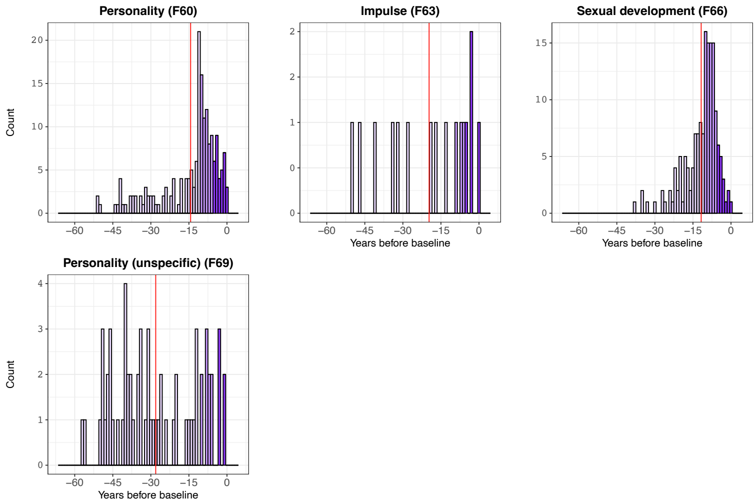


**Figure S9.** Differences in years between the first recorded diagnosis of mental/behavioural disorders and the baseline assessment date, shown for disorders of adult personality and behaviour (F60–F69). ICD-10 = International Classification of Diseases, 10th Revision. Vertical red lines show the mean difference. Sample sizes reported in Table S4.

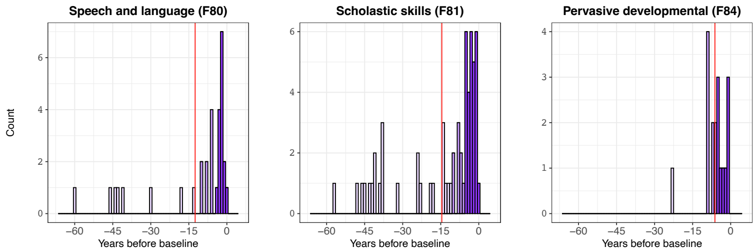


**Figure S10.** Differences in years between the first recorded diagnosis of mental/behavioural disorders and the baseline assessment date, shown for disorders of psychological development (F80–F89). ICD-10 = International Classification of Diseases, 10th Revision. Vertical red lines show the mean difference. Sample sizes reported in Table S4.

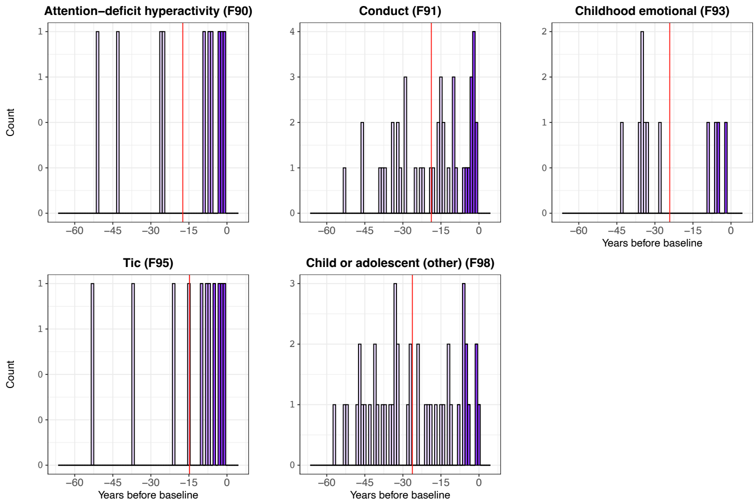


**Figure S11.** Differences in years between the first recorded diagnosis of mental/behavioural disorders and the baseline assessment date, shown for disorders of behavioural and emotional disorders with onset usually occurring in childhood and adolescence (F90–F98). ICD-10 = International Classification of Diseases, 10th Revision. Vertical red lines show the mean difference. Sample sizes reported in Table S4.

# 7. MileAge delta and individual two-digit ICD-10 codes

**Table S4.** MileAge delta and individual mental/behavioural disorders

| | | Model 1 (adj. age and sex) | | | | Model 2 (full adjustment) | | | |
|---|---|---|---|---|---|---|---|---|---|
| ICD-10 | *N* | *β* | 95% CI | | *p* | *β* | 95% CI | | *p* |
| None | 186688 | Reference | | | | Reference | | | |
| F10, Alcohol use | 1541 | 0.606 | 0.418 | 0.795 | <0.001 | 0.501 | 0.311 | 0.690 | <0.001 |
| F11, Opioid use | 60 | 1.268 | 0.318 | 2.219 | 0.045 | 1.054 | 0.103 | 2.004 | 0.091 |
| F12, Cannabis use | 46 | -0.914 | -1.999 | 0.172 | 0.255 | -1.108 | -2.193 | -0.023 | 0.127 |
| F13, Sedative use | 68 | -0.394 | -1.287 | 0.498 | 0.580 | -0.479 | -1.371 | 0.413 | 0.501 |
| F14, Cocaine use | 10 | 0.965 | -1.363 | 3.293 | 0.604 | 0.710 | -1.616 | 3.036 | 0.722 |
| F15, Stimulant use | 14 | 0.942 | -1.026 | 2.909 | 0.570 | 0.739 | -1.227 | 2.705 | 0.633 |
| F16, Hallucinogen use | 12 | 3.758 | 1.633 | 5.883 | 0.005 | 3.630 | 1.507 | 5.753 | 0.007 |
| F17, Tobacco use | 9715 | 0.409 | 0.332 | 0.485 | <0.001 | 0.373 | 0.296 | 0.450 | <0.001 |
| F19, Multi-substance use | 81 | 0.624 | -0.194 | 1.442 | 0.338 | 0.461 | -0.357 | 1.278 | 0.493 |
| F20, Schizophrenia | 386 | 0.859 | 0.484 | 1.235 | <0.001 | 0.638 | 0.261 | 1.014 | 0.007 |
| F22, Delusional | 88 | 0.956 | 0.171 | 1.741 | 0.065 | 0.804 | 0.020 | 1.589 | 0.127 |
| F23, Acute psychosis | 66 | 0.044 | -0.862 | 0.950 | 0.956 | -0.069 | -0.974 | 0.837 | 0.937 |
| F25, Schizoaffective | 85 | 0.905 | 0.106 | 1.703 | 0.085 | 0.729 | -0.069 | 1.527 | 0.200 |
| F29, Psychosis (unspecific) | 83 | 0.541 | -0.267 | 1.349 | 0.426 | 0.366 | -0.442 | 1.174 | 0.574 |
| F30, Mania | 124 | 0.494 | -0.167 | 1.155 | 0.348 | 0.408 | -0.252 | 1.069 | 0.474 |
| F31, Bipolar | 711 | 0.233 | -0.044 | 0.509 | 0.255 | 0.146 | -0.131 | 0.423 | 0.501 |
| F32, Depressive episode | 19176 | 0.274 | 0.218 | 0.331 | <0.001 | 0.230 | 0.173 | 0.286 | <0.001 |
| F33, Recurrent depression | 1211 | 0.327 | 0.115 | 0.539 | 0.015 | 0.268 | 0.056 | 0.480 | 0.055 |
| F34, Persistent mood | 306 | 0.247 | -0.174 | 0.668 | 0.480 | 0.190 | -0.231 | 0.611 | 0.574 |
| F38, Mood (other) | 36 | 0.300 | -0.927 | 1.527 | 0.758 | 0.237 | -0.989 | 1.462 | 0.824 |
| F39, Mood (unspecific) | 282 | 0.201 | -0.238 | 0.640 | 0.574 | 0.171 | -0.268 | 0.609 | 0.633 |
| F40, Phobic | 717 | 0.151 | -0.125 | 0.426 | 0.500 | 0.119 | -0.156 | 0.395 | 0.584 |
| F41, Anxiety | 8433 | 0.131 | 0.049 | 0.213 | 0.013 | 0.098 | 0.016 | 0.180 | 0.070 |
| F42, Obsessive-compulsive | 270 | -0.518 | -0.966 | -0.070 | 0.079 | -0.588 | -1.036 | -0.140 | 0.045 |
| F43, Stress-related | 4749 | 0.193 | 0.084 | 0.301 | 0.005 | 0.169 | 0.061 | 0.277 | 0.014 |
| F44, Dissociative | 79 | 0.374 | -0.454 | 1.203 | 0.574 | 0.317 | -0.510 | 1.145 | 0.633 |
| F45, Somatoform | 843 | 0.021 | -0.233 | 0.276 | 0.937 | 0.017 | -0.237 | 0.271 | 0.937 |
| F48, Other neurotic | 487 | 0.101 | -0.233 | 0.435 | 0.722 | 0.038 | -0.296 | 0.372 | 0.925 |
| F50, Eating | 364 | -0.588 | -0.975 | -0.201 | 0.016 | -0.613 | -1.000 | -0.227 | 0.013 |
| F51, Sleep | 153 | 0.367 | -0.228 | 0.963 | 0.474 | 0.362 | -0.232 | 0.957 | 0.475 |
| F52, Sexual dysfunction | 632 | -0.008 | -0.301 | 0.286 | 0.971 | -0.012 | -0.305 | 0.281 | 0.959 |
| F53, Puerperal psychosis | 629 | -0.389 | -0.683 | -0.094 | 0.045 | -0.371 | -0.666 | -0.077 | 0.055 |
| F60, Personality | 180 | 0.805 | 0.256 | 1.354 | 0.021 | 0.667 | 0.118 | 1.216 | 0.065 |
| F63, Impulse | 16 | 1.142 | -0.698 | 2.982 | 0.474 | 1.083 | -0.755 | 2.922 | 0.480 |
| F66, Sexual development | 155 | -0.333 | -0.924 | 0.259 | 0.493 | -0.351 | -0.942 | 0.240 | 0.480 |
| F69, Personality (unspecific) | 64 | -0.003 | -0.923 | 0.918 | 0.996 | -0.089 | -1.009 | 0.830 | 0.932 |
| F80, Speech and language | 31 | 0.441 | -0.881 | 1.763 | 0.690 | 0.375 | -0.946 | 1.696 | 0.722 |
| F81, Scholastic skills | 60 | 0.355 | -0.596 | 1.305 | 0.633 | 0.236 | -0.714 | 1.186 | 0.758 |
| F84, Pervasive developmental | 18 | 0.497 | -1.238 | 2.232 | 0.722 | 0.357 | -1.376 | 2.091 | 0.813 |
| F90, ADHD | 10 | -0.324 | -2.652 | 2.003 | 0.894 | -0.373 | -2.699 | 1.952 | 0.869 |
| F91, Conduct | 43 | 1.314 | 0.192 | 2.437 | 0.075 | 1.238 | 0.117 | 2.360 | 0.091 |
| F93, Childhood emotional | 11 | 0.650 | -1.570 | 2.869 | 0.722 | 0.594 | -1.624 | 2.811 | 0.739 |
| F95, Tic | 11 | 1.550 | -0.669 | 3.770 | 0.397 | 1.544 | -0.673 | 3.761 | 0.397 |
| F98, Child or adolescent (other) | 45 | -0.580 | -1.678 | 0.517 | 0.501 | -0.612 | -1.709 | 0.484 | 0.493 |
| F99, Unspecified | 86 | 0.085 | -0.708 | 0.879 | 0.925 | -0.059 | -0.852 | 0.735 | 0.937 |

*Note:* ICD-10 = International Classification of Diseases, 10th Revision; CI = confidence interval. Model 1–adjusted for chronological age and sex; Model 2–adjusted for chronological age, sex, ethnicity, cohabitation with spouse/partner, highest educational/professional qualification, annual gross household income, Townsend deprivation index and fasting time. Cells highlighted in grey and blue correspond to statistically significant associations (nominally and after multiple testing corrections, respectively). *P*-values shown are corrected for multiple testing using the Benjamini–Hochberg procedure.

## 8. MileAge delta and groups of two-digit ICD-10 codes; sex-stratified

**Table S5.** MileAge delta and groups of mental/behavioural disorders stratified by sex

| | Males | | | | | | | | | Females | | | | | | | | |
|---|---|---|---|---|---|---|---|---|---|---|---|---|---|---|---|---|---|---|
| | | Model 1 (adj. age) | | | | Model 2 (full adjustment) | | | | | Model 1 | | | | Model 2 | | | |
| ICD-10 | *N* | *β* | 95% CI | | *p* | *β* | 95% CI | | *p* | *N* | *β* | 95% CI | | *p* | *β* | 95% CI | | *p* |
| None | 88232 | Reference | | | | Reference | | | | 98456 | Reference | | | | Reference | | | |
| F10-99, Any disorder | 15449 | 0.362 | 0.298 | 0.426 | <0.001 | 0.313 | 0.248 | 0.377 | <0.001 | 23075 | 0.188 | 0.134 | 0.242 | <0.001 | 0.163 | 0.109 | 0.218 | <0.001 |
| F10-19, Substance use | 6040 | 0.524 | 0.427 | 0.621 | <0.001 | 0.467 | 0.369 | 0.566 | <0.001 | 5121 | 0.350 | 0.244 | 0.456 | <0.001 | 0.324 | 0.218 | 0.430 | <0.001 |
| F20-29, Psychosis | 343 | 0.967 | 0.572 | 1.363 | <0.001 | 0.765 | 0.368 | 1.163 | <0.001 | 244 | 0.350 | -0.123 | 0.823 | 0.215 | 0.239 | -0.235 | 0.712 | 0.431 |
| F30-39, Affective | 6992 | 0.358 | 0.267 | 0.449 | <0.001 | 0.300 | 0.208 | 0.392 | <0.001 | 13368 | 0.238 | 0.170 | 0.306 | <0.001 | 0.204 | 0.135 | 0.272 | <0.001 |
| F40-48, Neurotic | 4938 | 0.209 | 0.102 | 0.316 | <0.001 | 0.175 | 0.068 | 0.282 | 0.003 | 8978 | 0.136 | 0.055 | 0.217 | 0.003 | 0.115 | 0.033 | 0.196 | 0.013 |
| F50-59, Behavioural syndromes | 459 | 0.413 | 0.071 | 0.755 | 0.036 | 0.393 | 0.052 | 0.735 | 0.044 | 1304 | -0.309 | -0.515 | -0.103 | 0.008 | -0.312 | -0.518 | -0.106 | 0.008 |
| F60-69, Personality & behaviour | 250 | 0.121 | -0.342 | 0.584 | 0.788 | 0.042 | -0.421 | 0.504 | 0.911 | 183 | 0.608 | 0.062 | 1.154 | 0.058 | 0.550 | 0.004 | 1.096 | 0.089 |
| F70-79, Mental retardation | | *N* insufficient | | | | | | | | | *N* insufficient | | | | | | | |
| F80-89, Developmental | 68 | 0.408 | -0.479 | 1.294 | 0.539 | 0.290 | -0.597 | 1.176 | 0.717 | 47 | 0.405 | -0.671 | 1.482 | 0.533 | 0.317 | -0.759 | 1.393 | 0.620 |
| F90-98, Child & adolescent | 66 | 0.135 | -0.764 | 1.035 | 0.894 | 0.075 | -0.824 | 0.974 | 0.911 | 54 | 0.883 | -0.121 | 1.887 | 0.144 | 0.846 | -0.157 | 1.849 | 0.155 |
| F99, Unspecified | 35 | 0.183 | -1.053 | 1.418 | 0.894 | 0.001 | -1.233 | 1.236 | 0.998 | 51 | 0.068 | -0.965 | 1.101 | 0.940 | -0.032 | -1.065 | 1.001 | 0.952 |

*Note:* ICD-10 = International Classification of Diseases, 10th Revision; CI = confidence interval. Model 1–adjusted for chronological age; Model 2–adjusted for chronological age, ethnicity, cohabitation with spouse/partner, highest educational/professional qualification, annual gross household income, Townsend deprivation index and fasting time. Cells highlighted in grey and blue correspond to statistically significant associations (nominally and after multiple testing corrections, respectively). *P*-values shown are corrected for multiple testing using the Benjamini–Hochberg procedure within each sex.

## 9. MileAge delta and groups of two-digit ICD-10 codes; sex-interactions

**Table S6.** Associations between MileAge delta and mental/behavioural disorders

| | | | Model 1 (adj. age) | | | | Model 2 (full adjustment) | | | |
|---|---|---|---|---|---|---|---|---|---|---|
| ICD-10 | $N_{female}$ | $N_{male}$ | *β* | 95% CI | | *p* | *β* | 95% CI | | *p* |
| None | 98456 | 88232 | Reference | | | | Reference | | | |
| F10-99, Any disorder | 23075 | 15449 | 0.207 | 0.123 | 0.291 | <0.001 | 0.186 | 0.102 | 0.270 | <0.001 |
| F10-19, Substance use | 5121 | 6040 | 0.195 | 0.051 | 0.339 | 0.020 | 0.173 | 0.029 | 0.317 | 0.047 |
| F20-29, Psychosis | 244 | 343 | 0.749 | 0.132 | 1.367 | 0.035 | 0.669 | 0.052 | 1.286 | 0.067 |
| F30-39, Affective | 13368 | 6992 | 0.178 | 0.064 | 0.292 | 0.008 | 0.155 | 0.041 | 0.269 | 0.026 |
| F40-48, Neurotic | 8978 | 4938 | 0.105 | -0.030 | 0.240 | 0.213 | 0.095 | -0.040 | 0.230 | 0.279 |
| F50-59, Behavioural syndromes | 1304 | 459 | 0.783 | 0.382 | 1.184 | <0.001 | 0.763 | 0.362 | 1.164 | <0.001 |
| F60-69, Personality & behaviour | 183 | 250 | -0.363 | -1.080 | 0.354 | 0.459 | -0.368 | -1.084 | 0.349 | 0.449 |
| F70-79, Mental retardation | | | *N* insufficient | | | | | | | |
| F80-89, Developmental | 47 | 68 | 0.260 | -1.136 | 1.657 | 0.772 | 0.223 | -1.173 | 1.618 | 0.828 |
| F90-98, Child & adolescent | 54 | 66 | -0.565 | -1.916 | 0.786 | 0.515 | -0.599 | -1.949 | 0.751 | 0.480 |
| F99, Unspecified | 51 | 35 | 0.239 | -1.377 | 1.855 | 0.772 | 0.179 | -1.435 | 1.794 | 0.828 |

*Note:* ICD-10 = International Classification of Diseases 10th Revision; CI = confidence interval. Model 1–adjusted for chronological age; Model 2–adjusted for chronological age, ethnicity, cohabitation with spouse/partner, highest educational/professional qualification, annual gross household income, neighbourhood deprivation and fasting time. Cells highlighted in blue correspond to statistically significant associations after multiple testing correction. *P*-values shown are corrected for multiple testing using the Benjamini–Hochberg procedure.

## 10. MileAge delta and individual two-digit ICD-10 codes; sex-stratified

**Table S7.** MileAge delta and individual mental/behavioural disorders stratified by sex

| | | Males | | | | | | | | | Females | | | | | | | |
|---|---|---|---|---|---|---|---|---|---|---|---|---|---|---|---|---|---|---|
| | | Model 1 (adj. age) | | | | Model 2 (full adjustment) | | | | | Model 1 | | | | Model 2 | | | |
| ICD-10 | *N* | *β* | 95% CI | | *p* | *β* | 95% CI | | *p* | *N* | *β* | 95% CI | | *p* | *β* | 95% CI | | *p* |
| None | 88232 | Reference | | | | Reference | | | | 98456 | Reference | | | | Reference | | | |
| F10, Alcohol use | 1134 | 0.468 | 0.249 | 0.687 | <0.001 | 0.367 | 0.146 | 0.587 | 0.009 | 407 | 1.008 | 0.641 | 1.374 | <0.001 | 0.938 | 0.571 | 1.306 | <0.001 |
| F11, Opioid use | 44 | 1.376 | 0.275 | 2.478 | 0.076 | 1.139 | 0.037 | 2.242 | 0.151 | 16 | 0.613 | -1.231 | 2.457 | 0.786 | 0.562 | -1.281 | 2.405 | 0.786 |
| F12, Cannabis use | 34 | -1.523 | -2.776 | -0.270 | 0.085 | -1.688 | -2.941 | -0.434 | 0.056 | 12 | 0.521 | -1.609 | 2.651 | 0.818 | 0.323 | -1.806 | 2.451 | 0.841 |
| F13, Sedative use | 24 | -1.511 | -3.003 | -0.020 | 0.151 | -1.686 | -3.177 | -0.196 | 0.109 | 44 | 0.186 | -0.926 | 1.298 | 0.841 | 0.158 | -0.953 | 1.269 | 0.841 |
| F14, Cocaine use | | *N* insufficient | | | | | | | | | *N* insufficient | | | | | | | |
| F15, Stimulant use | 10 | 1.294 | -1.017 | 3.604 | 0.403 | 1.081 | -1.228 | 3.390 | 0.492 | | *N* insufficient | | | | | | | |
| F16, Hallucinogen use | | *N* insufficient | | | | | | | | | *N* insufficient | | | | | | | |
| F17, Tobacco use | 4997 | 0.535 | 0.429 | 0.641 | <0.001 | 0.488 | 0.381 | 0.595 | <0.001 | 4718 | 0.308 | 0.198 | 0.418 | <0.001 | 0.286 | 0.176 | 0.397 | <0.001 |
| F19, Multi-substance use | 50 | 0.367 | -0.666 | 1.401 | 0.643 | 0.186 | -0.847 | 1.220 | 0.864 | 31 | 0.869 | -0.456 | 2.194 | 0.440 | 0.780 | -0.545 | 2.104 | 0.497 |
| F20, Schizophrenia | 253 | 1.043 | 0.583 | 1.503 | <0.001 | 0.807 | 0.345 | 1.269 | 0.006 | 133 | 0.343 | -0.297 | 0.983 | 0.573 | 0.204 | -0.436 | 0.845 | 0.786 |
| F22, Delusional | 41 | 1.521 | 0.379 | 2.662 | 0.056 | 1.363 | 0.222 | 2.504 | 0.087 | 47 | 0.507 | -0.569 | 1.583 | 0.656 | 0.391 | -0.685 | 1.466 | 0.786 |
| F23, Acute psychosis | 29 | 1.209 | -0.148 | 2.566 | 0.239 | 1.105 | -0.252 | 2.461 | 0.296 | 37 | -0.797 | -2.010 | 0.416 | 0.440 | -0.889 | -2.101 | 0.324 | 0.395 |
| F25, Schizoaffective | 43 | 1.322 | 0.208 | 2.437 | 0.087 | 1.131 | 0.017 | 2.245 | 0.151 | 42 | 0.453 | -0.686 | 1.591 | 0.753 | 0.339 | -0.799 | 1.477 | 0.786 |
| F29, Psychosis (unspecific) | 40 | 0.590 | -0.565 | 1.746 | 0.442 | 0.401 | -0.754 | 1.556 | 0.644 | 43 | 0.467 | -0.658 | 1.592 | 0.735 | 0.356 | -0.768 | 1.481 | 0.786 |
| F30, Mania | 52 | -0.048 | -1.061 | 0.966 | 0.939 | -0.163 | -1.176 | 0.850 | 0.865 | 72 | 0.897 | 0.027 | 1.766 | 0.168 | 0.830 | -0.039 | 1.699 | 0.202 |
| F31, Bipolar | 293 | -0.137 | -0.565 | 0.291 | 0.677 | -0.228 | -0.656 | 0.200 | 0.423 | 418 | 0.537 | 0.175 | 0.898 | 0.040 | 0.467 | 0.105 | 0.829 | 0.062 |
| F32, Depressive episode | 6574 | 0.390 | 0.296 | 0.483 | <0.001 | 0.332 | 0.238 | 0.426 | <0.001 | 12602 | 0.232 | 0.162 | 0.302 | <0.001 | 0.197 | 0.127 | 0.268 | <0.001 |
| F33, Recurrent depression | 412 | 0.355 | -0.006 | 0.715 | 0.167 | 0.290 | -0.071 | 0.651 | 0.296 | 799 | 0.341 | 0.078 | 0.603 | 0.062 | 0.297 | 0.034 | 0.559 | 0.119 |
| F34, Persistent mood | 102 | 0.511 | -0.213 | 1.235 | 0.342 | 0.459 | -0.265 | 1.182 | 0.368 | 204 | 0.183 | -0.334 | 0.700 | 0.786 | 0.121 | -0.396 | 0.638 | 0.818 |
| F38, Mood (other) | | *N* insufficient | | | | | | | | 27 | 0.709 | -0.711 | 2.129 | 0.623 | 0.659 | -0.760 | 2.078 | 0.656 |
| F39, Mood (unspecific) | 60 | 0.762 | -0.182 | 1.705 | 0.296 | 0.694 | -0.248 | 1.637 | 0.342 | 222 | 0.144 | -0.352 | 0.640 | 0.786 | 0.124 | -0.372 | 0.619 | 0.818 |
| F40, Phobic | 164 | 0.458 | -0.113 | 1.029 | 0.296 | 0.405 | -0.165 | 0.976 | 0.342 | 553 | 0.052 | -0.263 | 0.366 | 0.841 | 0.028 | -0.287 | 0.342 | 0.886 |
| F41, Anxiety | 3009 | 0.139 | 0.003 | 0.274 | 0.151 | 0.096 | -0.040 | 0.232 | 0.342 | 5424 | 0.142 | 0.039 | 0.245 | 0.052 | 0.121 | 0.018 | 0.224 | 0.109 |
| F42, Obsessive-compulsive | 120 | -0.186 | -0.853 | 0.482 | 0.735 | -0.264 | -0.931 | 0.404 | 0.590 | 150 | -0.796 | -1.398 | -0.193 | 0.061 | -0.847 | -1.450 | -0.245 | 0.049 |
| F43, Stress-related | 1670 | 0.313 | 0.132 | 0.493 | 0.006 | 0.290 | 0.110 | 0.471 | 0.012 | 3079 | 0.155 | 0.020 | 0.290 | 0.117 | 0.130 | -0.005 | 0.266 | 0.202 |
| F44, Dissociative | 28 | -0.119 | -1.500 | 1.262 | 0.890 | -0.154 | -1.534 | 1.226 | 0.887 | 51 | 0.731 | -0.302 | 1.764 | 0.419 | 0.669 | -0.364 | 1.701 | 0.440 |
| F45, Somatoform | 293 | -0.037 | -0.465 | 0.390 | 0.890 | -0.049 | -0.476 | 0.378 | 0.887 | 550 | 0.046 | -0.269 | 0.361 | 0.841 | 0.047 | -0.268 | 0.362 | 0.841 |
| F48, Other neurotic | 201 | 0.372 | -0.144 | 0.888 | 0.342 | 0.302 | -0.214 | 0.817 | 0.396 | 286 | -0.061 | -0.498 | 0.375 | 0.841 | -0.107 | -0.543 | 0.330 | 0.818 |
| F50, Eating | 22 | 1.079 | -0.479 | 2.637 | 0.349 | 1.016 | -0.540 | 2.573 | 0.368 | 342 | -0.538 | -0.938 | -0.138 | 0.058 | -0.572 | -0.972 | -0.173 | 0.048 |
| F51, Sleep | 63 | 0.597 | -0.324 | 1.517 | 0.368 | 0.604 | -0.316 | 1.523 | 0.368 | 90 | 0.235 | -0.543 | 1.013 | 0.786 | 0.227 | -0.551 | 1.004 | 0.786 |
| F52, Sexual dysfunction | 368 | 0.301 | -0.081 | 0.683 | 0.302 | 0.281 | -0.100 | 0.662 | 0.342 | 264 | -0.292 | -0.746 | 0.163 | 0.440 | -0.286 | -0.740 | 0.168 | 0.447 |
| F53, Puerperal psychosis | | *N* insufficient | | | | | | | | 627 | -0.287 | -0.583 | 0.009 | 0.202 | -0.276 | -0.572 | 0.020 | 0.213 |
| F60, Personality | 78 | 1.070 | 0.242 | 1.898 | 0.064 | 0.906 | 0.078 | 1.734 | 0.124 | 102 | 0.657 | -0.073 | 1.388 | 0.237 | 0.576 | -0.155 | 1.307 | 0.333 |
| F63, Impulse | | *N* insufficient | | | | | | | | 10 | 2.075 | -0.258 | 4.407 | 0.238 | 2.017 | -0.315 | 4.348 | 0.253 |
| F66, Sexual development | 111 | -0.396 | -1.090 | 0.299 | 0.403 | -0.430 | -1.123 | 0.264 | 0.375 | 44 | 0.152 | -0.960 | 1.264 | 0.841 | 0.146 | -0.965 | 1.257 | 0.841 |

| | | | | | | | | | | | | | | | | | | |
|---|---|---|---|---|---|---|---|---|---|---|---|---|---|---|---|---|---|---|
| F69, Personality (unspecific) | 33 | -0.215 | -1.487 | 1.057 | 0.865 | -0.273 | -1.544 | 0.998 | 0.817 | 31 | 0.261 | -1.064 | 1.586 | 0.841 | 0.157 | -1.168 | 1.481 | 0.850 |
| F80, Speech and language | 21 | -0.141 | -1.736 | 1.453 | 0.890 | -0.185 | -1.778 | 1.408 | 0.887 | 10 | 1.598 | -0.734 | 3.931 | 0.440 | 1.516 | -0.815 | 3.847 | 0.440 |
| F81, Scholastic skills | 29 | 0.338 | -1.019 | 1.695 | 0.772 | 0.209 | -1.148 | 1.566 | 0.865 | 31 | 0.408 | -0.917 | 1.733 | 0.786 | 0.319 | -1.006 | 1.645 | 0.818 |
| F84, Pervasive developmental | 15 | 1.203 | -0.684 | 3.089 | 0.368 | 1.003 | -0.883 | 2.888 | 0.423 | | | | | *N* insufficient | | | | |
| F90, ADHD | | | | | *N* insufficient | | | | | | | | | *N* insufficient | | | | |
| F91, Conduct | 29 | 0.824 | -0.533 | 2.181 | 0.376 | 0.765 | -0.591 | 2.121 | 0.403 | 14 | 2.070 | 0.099 | 4.042 | 0.167 | 2.022 | 0.052 | 3.992 | 0.168 |
| F93, Childhood emotional | | | | | *N* insufficient | | | | | | | | | *N* insufficient | | | | |
| F95, Tic | | | | | *N* insufficient | | | | | | | | | *N* insufficient | | | | |
| F98, Child or adolescent (other) | 26 | -0.882 | -2.315 | 0.551 | 0.375 | -0.940 | -2.371 | 0.492 | 0.368 | 19 | -0.298 | -1.991 | 1.394 | 0.841 | -0.322 | -2.013 | 1.369 | 0.841 |
| F99, Unspecified | 35 | 0.183 | -1.053 | 1.418 | 0.865 | 0.001 | -1.233 | 1.236 | 0.998 | 51 | 0.068 | -0.965 | 1.101 | 0.909 | -0.032 | -1.065 | 1.001 | 0.952 |

*Note:* ICD-10 = International Classification of Diseases, 10th Revision; ADHD = attention-deficit/hyperactivity disorder; CI = confidence interval. Model 1–adjusted for chronological age; Model 2–adjusted for chronological age, ethnicity, cohabitation with spouse/partner, highest educational/professional qualification, annual gross household income, Townsend deprivation index and fasting time. Cells highlighted in grey and blue correspond to statistically significant associations (nominally and after multiple testing corrections, respectively). *P*-values shown are corrected for multiple testing using the Benjamini–Hochberg procedure within each sex.

## 11. MileAge delta and individual two-digit ICD-10 codes; sex-interactions

**Table S8.** MileAge delta and individual mental/behavioural disorders

| | | | Model 1 (adj. age) | | | | Model 2 (full adjustment) | | | |
|---|---|---|---|---|---|---|---|---|---|---|
| ICD-10 | *N* female | *N* male | *β* | 95% CI | | *p* | *β* | 95% CI | | *p* |
| None | 98456 | 88232 | Reference | | | | Reference | | | |
| F10, Alcohol use | 407 | 1134 | -0.456 | -0.883 | -0.029 | 0.197 | -0.495 | -0.921 | -0.068 | 0.159 |
| F11, Opioid use | 16 | 44 | 1.113 | -1.036 | 3.262 | 0.493 | 0.902 | -1.245 | 3.049 | 0.581 |
| F12, Cannabis use | 12 | 34 | -1.570 | -4.042 | 0.902 | 0.450 | -1.534 | -4.003 | 0.936 | 0.488 |
| F13, Sedative use | 44 | 24 | -1.896 | -3.764 | -0.028 | 0.197 | -2.044 | -3.911 | -0.178 | 0.159 |
| F14, Cocaine use | | | *N* insufficient | | | | | | | |
| F15, Stimulant use | | | *N* insufficient | | | | | | | |
| F16, Hallucinogen use | | | *N* insufficient | | | | | | | |
| F17, Tobacco use | 4718 | 4997 | 0.240 | 0.087 | 0.394 | 0.037 | 0.228 | 0.075 | 0.381 | 0.061 |
| F19, Multi-substance use | 31 | 50 | -0.354 | -2.037 | 1.329 | 0.794 | -0.442 | -2.124 | 1.239 | 0.757 |
| F20, Schizophrenia | 133 | 253 | 0.817 | 0.028 | 1.606 | 0.197 | 0.738 | -0.051 | 1.526 | 0.233 |
| F22, Delusional | 47 | 41 | 1.112 | -0.461 | 2.685 | 0.415 | 1.074 | -0.498 | 2.646 | 0.451 |
| F23, Acute psychosis | 37 | 29 | 2.227 | 0.401 | 4.052 | 0.156 | 2.216 | 0.392 | 4.040 | 0.159 |
| F25, Schizoaffective | 42 | 43 | 1.037 | -0.560 | 2.634 | 0.450 | 0.995 | -0.601 | 2.591 | 0.488 |
| F29, Psychosis (unspecific) | 43 | 40 | 0.309 | -1.308 | 1.927 | 0.799 | 0.221 | -1.394 | 1.837 | 0.871 |
| F30, Mania | 72 | 52 | -0.980 | -2.320 | 0.360 | 0.408 | -1.018 | -2.356 | 0.321 | 0.397 |
| F31, Bipolar | 418 | 293 | -0.600 | -1.162 | -0.038 | 0.197 | -0.629 | -1.190 | -0.067 | 0.159 |
| F32, Depressive episode | 12602 | 6574 | 0.217 | 0.099 | 0.334 | 0.010 | 0.195 | 0.077 | 0.312 | 0.040 |
| F33, Recurrent depression | 799 | 412 | 0.063 | -0.385 | 0.511 | 0.806 | 0.044 | -0.403 | 0.492 | 0.871 |
| F34, Persistent mood | 204 | 102 | 0.355 | -0.538 | 1.248 | 0.610 | 0.371 | -0.521 | 1.264 | 0.581 |
| F38, Mood (other) | | | *N* insufficient | | | | | | | |
| F39, Mood (unspecific) | 222 | 60 | 0.827 | -0.245 | 1.898 | 0.380 | 0.776 | -0.294 | 1.847 | 0.418 |
| F40, Phobic | 553 | 164 | 0.411 | -0.244 | 1.067 | 0.450 | 0.392 | -0.263 | 1.047 | 0.488 |
| F41, Anxiety | 5424 | 3009 | 0.037 | -0.134 | 0.208 | 0.794 | 0.019 | -0.152 | 0.190 | 0.871 |
| F42, Obsessive-compulsive | 150 | 120 | 0.734 | -0.168 | 1.636 | 0.352 | 0.703 | -0.198 | 1.604 | 0.397 |
| F43, Stress-related | 3079 | 1670 | 0.213 | -0.014 | 0.439 | 0.229 | 0.214 | -0.012 | 0.440 | 0.233 |
| F44, Dissociative | 51 | 28 | -0.938 | -2.669 | 0.794 | 0.481 | -0.892 | -2.622 | 0.838 | 0.546 |
| F45, Somatoform | 550 | 293 | -0.138 | -0.671 | 0.396 | 0.794 | -0.135 | -0.668 | 0.398 | 0.757 |
| F48, Other neurotic | 286 | 201 | 0.367 | -0.311 | 1.046 | 0.481 | 0.371 | -0.307 | 1.048 | 0.522 |
| F50, Eating | 342 | 22 | 1.958 | 0.339 | 3.578 | 0.156 | 1.872 | 0.254 | 3.490 | 0.159 |
| F51, Sleep | 90 | 63 | 0.306 | -0.904 | 1.515 | 0.794 | 0.302 | -0.906 | 1.510 | 0.757 |
| F52, Sexual dysfunction | 264 | 368 | 0.593 | -0.002 | 1.188 | 0.197 | 0.578 | -0.016 | 1.173 | 0.233 |
| F53, Puerperal psychosis | | | *N* insufficient | | | | | | | |
| F60, Personality | 102 | 78 | 0.618 | -0.490 | 1.726 | 0.481 | 0.544 | -0.563 | 1.651 | 0.559 |
| F63, Impulse | | | *N* insufficient | | | | | | | |
| F66, Sexual development | 44 | 111 | -0.597 | -1.909 | 0.714 | 0.566 | -0.612 | -1.923 | 0.698 | 0.572 |
| F69, Personality (unspecific) | 31 | 33 | -0.090 | -1.931 | 1.752 | 0.924 | -0.023 | -1.862 | 1.817 | 0.981 |
| F80, Speech and language | 10 | 21 | -1.726 | -4.554 | 1.102 | 0.450 | -1.655 | -4.481 | 1.171 | 0.488 |
| F81, Scholastic skills | 31 | 29 | 0.277 | -1.625 | 2.179 | 0.806 | 0.196 | -1.704 | 2.096 | 0.871 |
| F84, Pervasive developmental | | | *N* insufficient | | | | | | | |
| F90, ADHD | | | *N* insufficient | | | | | | | |
| F91, Conduct | 14 | 29 | -1.023 | -3.418 | 1.373 | 0.587 | -1.060 | -3.453 | 1.334 | 0.581 |
| F93, Childhood emotional | | | *N* insufficient | | | | | | | |
| F95, Tic | | | *N* insufficient | | | | | | | |
| F98, Child or adolescent (other) | 19 | 26 | -0.531 | -2.753 | 1.691 | 0.794 | -0.550 | -2.770 | 1.669 | 0.757 |
| F99, Unspecified | 51 | 35 | 0.239 | -1.377 | 1.855 | 0.806 | 0.179 | -1.435 | 1.794 | 0.871 |

*Note:* ICD-10 = International Classification of Diseases, 10th Revision; CI = confidence interval. Model 1–adjusted for chronological age; Model 2–adjusted for chronological age, ethnicity, cohabitation with spouse/partner, highest educational/professional qualification, annual gross household income, Townsend deprivation index and fasting time. Cells highlighted in grey and blue correspond to statistically significant associations (nominally and after multiple testing corrections, respectively). *P*-values shown are corrected for multiple testing using the Benjamini–Hochberg procedure.

## 12. MileAge delta and groups of two-digit ICD-10 codes; age-stratified

**Table S9.** MileAge delta and groups of mental/behavioural disorders stratified by age group

| | | < 65 years | | | | | | | | ≥ 65 years | | | | | | | | |
|---|---|---|---|---|---|---|---|---|---|---|---|---|---|---|---|---|---|---|
| | | Model 1 (adj. age and sex) | | | | Model 2 (full adjustment) | | | | | Model 1 | | | | Model 2 | | | |
| ICD-10 | *N* | *β* | 95% CI | | *p* | *β* | 95% CI | | *p* | *N* | *β* | 95% CI | | *p* | *β* | 95% CI | | *p* |
| None | 150342 | Reference | | | | Reference | | | | 36346 | Reference | | | | Reference | | | |
| F10-99, Any disorder | 32320 | 0.274 | 0.228 | 0.320 | <0.001 | 0.227 | 0.181 | 0.274 | <0.001 | 6204 | 0.090 | -0.004 | 0.183 | 0.405 | 0.078 | -0.016 | 0.172 | 0.523 |
| F10-19, Substance use | 9165 | 0.435 | 0.354 | 0.515 | <0.001 | 0.374 | 0.293 | 0.455 | <0.001 | 1996 | 0.400 | 0.243 | 0.556 | <0.001 | 0.386 | 0.229 | 0.543 | <0.001 |
| F20-29, Psychosis | 507 | 0.876 | 0.543 | 1.209 | <0.001 | 0.630 | 0.296 | 0.965 | <0.001 | 80 | -0.148 | -0.908 | 0.611 | 0.920 | -0.147 | -0.907 | 0.613 | 0.920 |
| F30-39, Affective | 17467 | 0.313 | 0.252 | 0.373 | <0.001 | 0.255 | 0.194 | 0.316 | <0.001 | 2893 | -0.038 | -0.170 | 0.093 | 0.920 | -0.051 | -0.183 | 0.081 | 0.896 |
| F40-48, Neurotic | 11729 | 0.174 | 0.102 | 0.246 | <0.001 | 0.138 | 0.066 | 0.210 | <0.001 | 2187 | 0.000 | -0.150 | 0.150 | >0.999 | -0.016 | -0.166 | 0.134 | 0.962 |
| F50-59, Behavioural syndromes | 1546 | -0.265 | -0.457 | -0.074 | 0.012 | -0.269 | -0.460 | -0.077 | 0.012 | 217 | 0.292 | -0.170 | 0.754 | 0.720 | 0.301 | -0.161 | 0.763 | 0.720 |
| F60-69, Personality & behaviour | 371 | 0.404 | 0.014 | 0.793 | 0.071 | 0.290 | -0.099 | 0.679 | 0.199 | 62 | -0.409 | -1.272 | 0.454 | 0.883 | -0.430 | -1.293 | 0.433 | 0.883 |
| F70-79, Mental retardation | 14 | 1.656 | -0.345 | 3.657 | 0.165 | 1.423 | -0.576 | 3.422 | 0.199 | | *N* insufficient | | | | | | | |
| F80-89, Developmental | 103 | 0.535 | -0.203 | 1.273 | 0.199 | 0.385 | -0.353 | 1.123 | 0.337 | 12 | -0.337 | -2.296 | 1.623 | 0.920 | -0.374 | -2.333 | 1.584 | 0.920 |
| F90-98, Child & adolescent | 106 | 0.573 | -0.154 | 1.300 | 0.180 | 0.495 | -0.231 | 1.222 | 0.210 | 14 | 0.100 | -1.714 | 1.915 | 0.962 | 0.132 | -1.681 | 1.946 | 0.962 |
| F99, Unspecified | 73 | 0.003 | -0.873 | 0.880 | 0.994 | -0.182 | -1.058 | 0.693 | 0.716 | 13 | 0.638 | -1.245 | 2.521 | 0.920 | 0.767 | -1.117 | 2.651 | 0.896 |

*Note:* ICD-10 = International Classification of Diseases, 10th Revision; CI = confidence interval. Model 1–adjusted for chronological age and sex; Model 2–adjusted for chronological age, sex, ethnicity, cohabitation with spouse/partner, highest educational/professional qualification, annual gross household income, Townsend deprivation index and fasting time. Cells highlighted in grey and blue correspond to statistically significant associations (nominally and after multiple testing corrections, respectively). *P*-values shown are corrected for multiple testing using the Benjamini–Hochberg procedure within each age group.

## 13. MileAge delta and groups of two-digit ICD-10 codes; age-interactions

**Table S10.** Associations between MileAge delta and mental/behavioural disorders

| | | | Model 1 (adj. age and sex) | | | | Model 2 (full adjustment) | | | |
|---|---|---|---|---|---|---|---|---|---|---|
| ICD-10 | $N_{<65}$ | $N_{\geq 65}$ | *β* | 95% CI | | *p* | *β* | 95% CI | | *p* |
| None | 150342 | 36346 | Reference | | | | Reference | | | |
| F10-99, Any disorder | 32320 | 6204 | -0.160 | -0.271 | -0.049 | 0.024 | -0.148 | -0.259 | -0.037 | 0.044 |
| F10-19, Substance use | 9165 | 1996 | -0.058 | -0.245 | 0.129 | 0.636 | -0.038 | -0.224 | 0.149 | 0.741 |
| F20-29, Psychosis | 507 | 80 | -1.028 | -1.914 | -0.142 | 0.077 | -0.932 | -1.818 | -0.047 | 0.098 |
| F30-39, Affective | 17467 | 2893 | -0.301 | -0.455 | -0.147 | 0.001 | -0.287 | -0.441 | -0.133 | 0.003 |
| F40-48, Neurotic | 11729 | 2187 | -0.123 | -0.300 | 0.054 | 0.287 | -0.115 | -0.292 | 0.061 | 0.334 |
| F50-59, Behavioural syndromes | 1546 | 217 | 0.589 | 0.053 | 1.124 | 0.078 | 0.598 | 0.063 | 1.133 | 0.095 |
| F60-69, Personality & behaviour | 371 | 62 | -0.850 | -1.860 | 0.160 | 0.198 | -0.752 | -1.761 | 0.258 | 0.289 |
| F70-79, Mental retardation | | | *N* insufficient | | | | | | | |
| F80-89, Developmental | 103 | 12 | -0.816 | -3.061 | 1.429 | 0.636 | -0.763 | -3.005 | 1.480 | 0.696 |
| F90-98, Child & adolescent | 106 | 14 | -0.455 | -2.548 | 1.637 | 0.670 | -0.353 | -2.443 | 1.738 | 0.741 |
| F99, Unspecified | 73 | 13 | 0.638 | -1.578 | 2.853 | 0.636 | 0.664 | -1.550 | 2.877 | 0.696 |

*Note:* ICD-10 = International Classification of Diseases 10th Revision; CI = confidence interval. Model 1–adjusted for chronological age; Model 2–adjusted for chronological age, sex, ethnicity, cohabitation with spouse/partner, highest educational/professional qualification, annual gross household income, neighbourhood deprivation and fasting time. Cells highlighted in blue correspond to statistically significant associations after multiple testing correction. *P*-values shown are corrected for multiple testing using the Benjamini–Hochberg procedure.

## 14. MileAge delta and individual two-digit ICD-10 codes; age-stratified

**Table S11.** MileAge delta and individual mental/behavioural disorders stratified by age group

| | < 65 years | | | | | | | | | | ≥ 65 years | | | | | | | | |
|---|---|---|---|---|---|---|---|---|---|---|---|---|---|---|---|---|---|---|
| | | Model 1 (adj. age and sex) | | | | Model 2 (full adjustment) | | | | | Model 1 | | | | Model 2 | | | |
| ICD-10 | *N* | *β* | 95% CI | | *p* | *β* | 95% CI | | *p* | *N* | *β* | 95% CI | | *p* | *β* | 95% CI | | *p* |
| None | 150342 | Reference | | | | Reference | | | | 36346 | Reference | | | | Reference | | | |
| F10, Alcohol use | 1326 | 0.640 | 0.433 | 0.847 | <0.001 | 0.499 | 0.291 | 0.707 | <0.001 | 215 | 0.288 | -0.177 | 0.753 | 0.818 | 0.281 | -0.184 | 0.747 | 0.818 |
| F11, Opioid use | 58 | 1.207 | 0.224 | 2.191 | 0.060 | 0.939 | -0.043 | 1.922 | 0.175 | | *N* insufficient | | | | | | | |
| F12, Cannabis use | 44 | -0.851 | -1.979 | 0.278 | 0.325 | -1.094 | -2.222 | 0.034 | 0.170 | | *N* insufficient | | | | | | | |
| F13, Sedative use | 50 | -0.645 | -1.704 | 0.414 | 0.412 | -0.765 | -1.823 | 0.293 | 0.336 | 18 | 0.253 | -1.347 | 1.853 | 0.906 | 0.305 | -1.295 | 1.904 | 0.906 |
| F14, Cocaine use | 10 | 0.925 | -1.442 | 3.293 | 0.594 | 0.607 | -1.758 | 2.972 | 0.745 | | *N* insufficient | | | | | | | |
| F15, Stimulant use | 13 | 1.386 | -0.690 | 3.462 | 0.373 | 1.144 | -0.930 | 3.218 | 0.437 | | *N* insufficient | | | | | | | |
| F16, Hallucinogen use | 10 | 5.036 | 2.668 | 7.403 | <0.001 | 4.827 | 2.462 | 7.192 | <0.001 | | *N* insufficient | | | | | | | |
| F17, Tobacco use | 7925 | 0.405 | 0.319 | 0.491 | <0.001 | 0.358 | 0.271 | 0.445 | <0.001 | 1790 | 0.423 | 0.258 | 0.587 | <0.001 | 0.406 | 0.241 | 0.571 | <0.001 |
| F19, Multi-substance use | 67 | 0.636 | -0.279 | 1.551 | 0.363 | 0.408 | -0.506 | 1.323 | 0.544 | 14 | 0.589 | -1.225 | 2.403 | 0.818 | 0.580 | -1.233 | 2.394 | 0.818 |
| F20, Schizophrenia | 332 | 1.041 | 0.629 | 1.452 | <0.001 | 0.750 | 0.337 | 1.162 | 0.003 | 54 | -0.314 | -1.239 | 0.610 | 0.818 | -0.293 | -1.218 | 0.632 | 0.818 |
| F22, Delusional | 74 | 0.944 | 0.073 | 1.814 | 0.111 | 0.744 | -0.126 | 1.613 | 0.252 | 14 | 1.055 | -0.759 | 2.870 | 0.818 | 0.994 | -0.821 | 2.808 | 0.818 |
| F23, Acute psychosis | 58 | 0.094 | -0.889 | 1.077 | 0.915 | -0.072 | -1.055 | 0.910 | 0.931 | | *N* insufficient | | | | | | | |
| F25, Schizoaffective | 76 | 0.876 | 0.017 | 1.735 | 0.140 | 0.654 | -0.204 | 1.513 | 0.323 | | *N* insufficient | | | | | | | |
| F29, Psychosis (unspecific) | 76 | 0.641 | -0.218 | 1.500 | 0.325 | 0.403 | -0.456 | 1.261 | 0.522 | | *N* insufficient | | | | | | | |
| F30, Mania | 97 | 0.582 | -0.179 | 1.342 | 0.323 | 0.451 | -0.309 | 1.211 | 0.412 | 27 | 0.197 | -1.110 | 1.504 | 0.906 | 0.209 | -1.098 | 1.515 | 0.906 |
| F31, Bipolar | 606 | 0.369 | 0.065 | 0.674 | 0.063 | 0.252 | -0.053 | 0.557 | 0.265 | 105 | -0.539 | -1.203 | 0.124 | 0.739 | -0.535 | -1.199 | 0.128 | 0.739 |
| F32, Depressive episode | 16474 | 0.317 | 0.255 | 0.378 | <0.001 | 0.259 | 0.197 | 0.322 | <0.001 | 2702 | -0.014 | -0.150 | 0.122 | 0.906 | -0.028 | -0.164 | 0.109 | 0.906 |
| F33, Recurrent depression | 1034 | 0.412 | 0.178 | 0.645 | 0.004 | 0.334 | 0.101 | 0.568 | 0.024 | 177 | -0.183 | -0.695 | 0.328 | 0.818 | -0.204 | -0.715 | 0.308 | 0.818 |
| F34, Persistent mood | 257 | 0.247 | -0.221 | 0.714 | 0.463 | 0.181 | -0.286 | 0.647 | 0.594 | 49 | 0.259 | -0.711 | 1.230 | 0.885 | 0.25 | -0.720 | 1.221 | 0.885 |
| F38, Mood (other) | 32 | 0.381 | -0.942 | 1.705 | 0.714 | 0.324 | -0.998 | 1.645 | 0.754 | | *N* insufficient | | | | | | | |
| F39, Mood (unspecific) | 262 | 0.285 | -0.178 | 0.748 | 0.412 | 0.257 | -0.206 | 0.720 | 0.437 | 20 | -0.65 | -2.169 | 0.868 | 0.818 | -0.668 | -2.186 | 0.850 | 0.818 |
| F40, Phobic | 582 | -0.005 | -0.316 | 0.306 | 0.986 | -0.041 | -0.352 | 0.269 | 0.864 | 135 | 0.831 | 0.246 | 1.416 | 0.085 | 0.812 | 0.227 | 1.397 | 0.085 |
| F41, Anxiety | 7116 | 0.150 | 0.059 | 0.241 | 0.007 | 0.109 | 0.018 | 0.200 | 0.067 | 1317 | -0.013 | -0.204 | 0.178 | 0.906 | -0.030 | -0.221 | 0.161 | 0.906 |
| F42, Obsessive-compulsive | 240 | -0.510 | -0.993 | -0.026 | 0.124 | -0.597 | -1.080 | -0.114 | 0.060 | 30 | -0.632 | -1.872 | 0.608 | 0.818 | -0.685 | -1.924 | 0.554 | 0.818 |
| F43, Stress-related | 4115 | 0.216 | 0.098 | 0.335 | 0.003 | 0.187 | 0.069 | 0.306 | 0.011 | 634 | -0.021 | -0.293 | 0.252 | 0.906 | -0.041 | -0.313 | 0.231 | 0.906 |
| F44, Dissociative | 64 | 0.679 | -0.257 | 1.615 | 0.336 | 0.595 | -0.340 | 1.530 | 0.405 | 15 | -0.928 | -2.681 | 0.825 | 0.818 | -0.884 | -2.637 | 0.868 | 0.818 |
| F45, Somatoform | 668 | 0.175 | -0.115 | 0.465 | 0.412 | 0.168 | -0.122 | 0.458 | 0.424 | 175 | -0.592 | -1.107 | -0.077 | 0.241 | -0.578 | -1.092 | -0.063 | 0.241 |
| F48, Other neurotic | 396 | 0.021 | -0.356 | 0.397 | 0.947 | -0.063 | -0.439 | 0.314 | 0.831 | 91 | 0.378 | -0.334 | 1.090 | 0.818 | 0.347 | -0.366 | 1.059 | 0.818 |
| F50, Eating | 354 | -0.579 | -0.978 | -0.180 | 0.023 | -0.605 | -1.003 | -0.206 | 0.016 | 10 | 0.174 | -1.973 | 2.321 | 0.906 | 0.130 | -2.016 | 2.275 | 0.906 |
| F51, Sleep | 119 | 0.349 | -0.338 | 1.035 | 0.482 | 0.343 | -0.342 | 1.029 | 0.484 | 34 | 0.457 | -0.707 | 1.622 | 0.818 | 0.471 | -0.693 | 1.635 | 0.818 |
| F52, Sexual dysfunction | 515 | -0.077 | -0.407 | 0.254 | 0.764 | -0.092 | -0.422 | 0.238 | 0.719 | 117 | 0.302 | -0.327 | 0.931 | 0.818 | 0.321 | -0.308 | 0.950 | 0.818 |
| F53, Puerperal psychosis | 572 | -0.412 | -0.727 | -0.098 | 0.044 | -0.389 | -0.703 | -0.075 | 0.060 | 57 | 0.098 | -0.802 | 0.998 | 0.906 | 0.089 | -0.811 | 0.989 | 0.906 |
| F60, Personality | 163 | 1.004 | 0.417 | 1.591 | 0.005 | 0.808 | 0.221 | 1.395 | 0.032 | 17 | -1.105 | -2.751 | 0.542 | 0.818 | -1.065 | -2.712 | 0.583 | 0.818 |
| F63, Impulse | 15 | 0.855 | -1.078 | 2.788 | 0.544 | 0.768 | -1.162 | 2.699 | 0.594 | | *N* insufficient | | | | | | | |
| F66, Sexual development | 116 | -0.390 | -1.085 | 0.305 | 0.437 | -0.415 | -1.109 | 0.280 | 0.412 | 39 | -0.142 | -1.230 | 0.946 | 0.906 | -0.183 | -1.271 | 0.904 | 0.906 |

| | | | | | | | | | | | | | | | | | | |
|---|---|---|---|---|---|---|---|---|---|---|---|---|---|---|---|---|---|---|
| F69, Personality (unspecific) | 63 | 0.033 | -0.911 | 0.976 | 0.968 | -0.068 | -1.010 | 0.874 | 0.931 | *N* insufficient | | | | | | | | |
| F80, Speech and language | 26 | 0.302 | -1.167 | 1.770 | 0.778 | 0.212 | -1.254 | 1.679 | 0.856 | *N* insufficient | | | | | | | | |
| F81, Scholastic skills | 56 | 0.595 | -0.406 | 1.596 | 0.412 | 0.435 | -0.565 | 1.435 | 0.546 | *N* insufficient | | | | | | | | |
| F84, Pervasive developmental | 16 | 0.568 | -1.304 | 2.440 | 0.702 | 0.387 | -1.483 | 2.256 | 0.778 | *N* insufficient | | | | | | | | |
| F90, ADHD | *N* insufficient | | | | | | | | | *N* insufficient | | | | | | | | |
| F91, Conduct | 40 | 1.099 | -0.084 | 2.283 | 0.191 | 0.996 | -0.186 | 2.179 | 0.257 | *N* insufficient | | | | | | | | |
| F93, Childhood emotional | *N* insufficient | | | | | | | | | *N* insufficient | | | | | | | | |
| F95, Tic | 11 | 1.538 | -0.719 | 3.795 | 0.365 | 1.533 | -0.721 | 3.787 | 0.365 | *N* insufficient | | | | | | | | |
| F98, Child or adolescent (other) | 37 | -0.371 | -1.601 | 0.860 | 0.702 | -0.430 | -1.659 | 0.799 | 0.642 | *N* insufficient | | | | | | | | |
| F99, Unspecified | 73 | 0.003 | -0.873 | 0.880 | 0.994 | -0.182 | -1.058 | 0.693 | 0.778 | 13 | 0.638 | -1.245 | 2.521 | 0.818 | 0.767 | -1.117 | 2.651 | 0.818 |

*Note:* ICD-10 = International Classification of Diseases, 10th Revision; ADHD = attention-deficit/hyperactivity disorder; CI = confidence interval. Model 1–adjusted for chronological age and sex; Model 2–adjusted for chronological age, sex, ethnicity, cohabitation with spouse/partner, highest educational/professional qualification, annual gross household income, Townsend deprivation index and fasting time. Cells highlighted in grey and blue correspond to statistically significant associations (nominally and after multiple testing corrections, respectively). *P*-values shown are corrected for multiple testing using the Benjamini–Hochberg procedure within each age group.

## 15. MileAge delta and individual two-digit ICD-10 codes; age-interactions

**Table S12.** MileAge delta and individual mental/behavioural disorders

| | | | Model 1 (adj. age and sex) | | | | Model 2 (full adjustment) | | | |
|---|---|---|---|---|---|---|---|---|---|---|
| ICD-10 | $N_{<65}$ | $N_{\geq 65}$ | *β* | 95% CI | | *p* | *β* | 95% CI | | *p* |
| None | 150342 | 36346 | Reference | | | | Reference | | | |
| F10, Alcohol use | 1326 | 215 | -0.437 | -0.979 | 0.106 | 0.374 | -0.373 | -0.915 | 0.170 | 0.498 |
| F11, Opioid use | | | *N* insufficient | | | | | | | |
| F12, Cannabis use | | | *N* insufficient | | | | | | | |
| F13, Sedative use | 50 | 18 | 0.937 | -1.086 | 2.960 | 0.661 | 0.976 | -1.045 | 2.997 | 0.634 |
| F14, Cocaine use | | | *N* insufficient | | | | | | | |
| F15, Stimulant use | | | *N* insufficient | | | | | | | |
| F16, Hallucinogen use | | | *N* insufficient | | | | | | | |
| F17, Tobacco use | 7925 | 1790 | 0.001 | -0.196 | 0.198 | 0.993 | 0.012 | -0.185 | 0.209 | 0.917 |
| F19, Multi-substance use | 67 | 14 | -0.015 | -2.178 | 2.147 | 0.993 | 0.115 | -2.045 | 2.276 | 0.917 |
| F20, Schizophrenia | 332 | 54 | -1.360 | -2.440 | -0.279 | 0.118 | -1.267 | -2.347 | -0.187 | 0.163 |
| F22, Delusional | 74 | 14 | 0.125 | -2.019 | 2.270 | 0.990 | 0.183 | -1.960 | 2.325 | 0.917 |
| F23, Acute psychosis | | | *N* insufficient | | | | | | | |
| F25, Schizoaffective | | | *N* insufficient | | | | | | | |
| F29, Psychosis (unspecific) | | | *N* insufficient | | | | | | | |
| F30, Mania | 97 | 27 | -0.365 | -1.967 | 1.236 | 0.896 | -0.284 | -1.884 | 1.316 | 0.917 |
| F31, Bipolar | 606 | 105 | -0.899 | -1.679 | -0.120 | 0.126 | -0.853 | -1.631 | -0.074 | 0.165 |
| F32, Depressive episode | 16474 | 2702 | -0.279 | -0.438 | -0.120 | 0.015 | -0.264 | -0.423 | -0.105 | 0.029 |
| F33, Recurrent depression | 1034 | 177 | -0.558 | -1.158 | 0.042 | 0.253 | -0.538 | -1.138 | 0.061 | 0.291 |
| F34, Persistent mood | 257 | 49 | 0.064 | -1.084 | 1.211 | 0.990 | 0.070 | -1.077 | 1.217 | 0.917 |
| F38, Mood (other) | | | *N* insufficient | | | | | | | |
| F39, Mood (unspecific) | 262 | 20 | -0.926 | -2.634 | 0.781 | 0.623 | -0.989 | -2.695 | 0.717 | 0.554 |
| F40, Phobic | 582 | 135 | 0.905 | 0.201 | 1.609 | 0.118 | 0.903 | 0.199 | 1.606 | 0.155 |
| F41, Anxiety | 7116 | 1317 | -0.108 | -0.333 | 0.117 | 0.661 | -0.095 | -0.320 | 0.130 | 0.663 |
| F42, Obsessive-compulsive | 240 | 30 | -0.131 | -1.557 | 1.294 | 0.990 | -0.105 | -1.529 | 1.319 | 0.917 |
| F43, Stress-related | 4115 | 634 | -0.185 | -0.502 | 0.132 | 0.595 | -0.188 | -0.505 | 0.129 | 0.554 |
| F44, Dissociative | 64 | 15 | -1.605 | -3.716 | 0.506 | 0.393 | -1.592 | -3.701 | 0.517 | 0.452 |
| F45, Somatoform | 668 | 175 | -0.720 | -1.346 | -0.094 | 0.126 | -0.715 | -1.341 | -0.089 | 0.163 |
| F48, Other neurotic | 396 | 91 | 0.383 | -0.474 | 1.239 | 0.661 | 0.402 | -0.453 | 1.258 | 0.634 |
| F50, Eating | 354 | 10 | 0.888 | -1.472 | 3.248 | 0.705 | 0.772 | -1.586 | 3.130 | 0.797 |
| F51, Sleep | 119 | 34 | 0.119 | -1.312 | 1.550 | 0.990 | 0.126 | -1.304 | 1.556 | 0.917 |
| F52, Sexual dysfunction | 515 | 117 | 0.312 | -0.443 | 1.067 | 0.679 | 0.348 | -0.406 | 1.102 | 0.634 |
| F53, Puerperal psychosis | 572 | 57 | 0.701 | -0.322 | 1.724 | 0.466 | 0.681 | -0.341 | 1.703 | 0.498 |
| F60, Personality | 163 | 17 | -2.052 | -3.928 | -0.176 | 0.139 | -1.850 | -3.724 | 0.025 | 0.230 |
| F63, Impulse | | | *N* insufficient | | | | | | | |
| F66, Sexual development | 116 | 39 | 0.161 | -1.202 | 1.523 | 0.990 | 0.179 | -1.182 | 1.540 | 0.917 |
| F69, Personality (unspecific) | | | *N* insufficient | | | | | | | |
| F80, Speech and language | | | *N* insufficient | | | | | | | |
| F81, Scholastic skills | | | *N* insufficient | | | | | | | |
| F84, Pervasive developmental | | | *N* insufficient | | | | | | | |
| F90, ADHD | | | *N* insufficient | | | | | | | |
| F91, Conduct | | | *N* insufficient | | | | | | | |
| F93, Childhood emotional | | | *N* insufficient | | | | | | | |
| F95, Tic | | | *N* insufficient | | | | | | | |
| F98, Child or adolescent (other) | | | *N* insufficient | | | | | | | |
| F99, Unspecified | 73 | 13 | 0.638 | -1.578 | 2.853 | 0.827 | 0.664 | -1.550 | 2.877 | 0.804 |

*Note:* ICD-10 = International Classification of Diseases, 10th Revision; CI = confidence interval. Model 1–adjusted for chronological age and sex; Model 2–adjusted for chronological age, sex ethnicity, cohabitation with spouse/partner, highest educational/professional qualification, annual gross household income, Townsend deprivation index and fasting time. Cells highlighted in grey and blue correspond to statistically significant associations (nominally and after multiple testing corrections, respectively). *P*-values shown are corrected for multiple testing using the Benjamini–Hochberg procedure.

## 16. MileAge delta and polygenic scores for groups of disorders

**Table S13.** MileAge delta and polygenic scores for groups of disorders across ancestries

| Polygenic score | *β* | 95% CI | | *p* |
|---|---|---|---|---|
| F10-99, Any disorder | 0.003 | -0.013 | 0.020 | 0.878 |
| F10-19, Substance use | 0.018 | 0.002 | 0.034 | 0.141 |
| F20-29, Psychosis | -0.036 | -0.052 | -0.020 | <0.001 |
| F30-39, Affective | 0.008 | -0.009 | 0.024 | 0.762 |
| F40-48, Neurotic | -0.001 | -0.018 | 0.016 | 0.974 |
| F50-59, Behavioural syndromes | 0.000 | -0.017 | 0.016 | 0.974 |
| F70-79, Mental retardation | 0.007 | -0.010 | 0.023 | 0.762 |
| F80-89, Developmental | 0.009 | -0.008 | 0.025 | 0.762 |
| F90-98, Child & adolescent | 0.004 | -0.012 | 0.019 | 0.878 |

*Note:* CI = confidence interval. Models were adjusted for chronological age, sex, assessment centre, batch number, the first six genetic principal components and fasting time. Cells highlighted in grey and blue correspond to statistically significant associations (nominally and after multiple testing corrections, respectively). *P*-values shown are corrected for multiple testing using the Benjamini–Hochberg procedure. *N* = 219,494.

**Table S14.** Ancestry-specific associations between MileAge delta and polygenic scores for groups of disorders

| Polygenic score | Population | *β* | 95% CI | | *p* |
|---|---|---|---|---|---|
| F10-99, Any disorder | AFR | 0.041 | -0.102 | 0.183 | 0.891 |
| | AMR | 0.601 | 0.065 | 1.136 | 0.127 |
| | CSA | 0.038 | -0.092 | 0.167 | 0.733 |
| | EAS | -0.097 | -0.388 | 0.195 | 0.662 |
| | EUR | 0.002 | -0.016 | 0.019 | 0.967 |
| | MID | 0.205 | -0.838 | 1.248 | 0.783 |
| F10-19, Substance use | AFR | -0.014 | -0.159 | 0.131 | 0.891 |
| | AMR | 1.011 | 0.354 | 1.669 | 0.025 |
| | CSA | 0.071 | -0.058 | 0.201 | 0.631 |
| | EAS | 0.165 | -0.121 | 0.452 | 0.637 |
| | EUR | 0.016 | -0.001 | 0.032 | 0.290 |
| | MID | -0.504 | -1.596 | 0.587 | 0.783 |
| F20-29, Psychosis | AFR | -0.009 | -0.140 | 0.122 | 0.891 |
| | AMR | 0.060 | -0.641 | 0.760 | 0.867 |
| | CSA | -0.097 | -0.232 | 0.038 | 0.553 |
| | EAS | 0.133 | -0.148 | 0.414 | 0.637 |
| | EUR | -0.038 | -0.055 | -0.021 | <0.001 |
| | MID | 0.705 | -0.290 | 1.700 | 0.728 |
| F30-39, Affective | AFR | -0.038 | -0.185 | 0.109 | 0.891 |
| | AMR | 0.448 | -0.175 | 1.071 | 0.237 |
| | CSA | 0.092 | -0.044 | 0.228 | 0.553 |
| | EAS | -0.166 | -0.453 | 0.121 | 0.637 |
| | EUR | 0.007 | -0.010 | 0.024 | 0.794 |
| | MID | 0.115 | -0.986 | 1.215 | 0.836 |
| F40-48, Neurotic | AFR | 0.027 | -0.118 | 0.172 | 0.891 |
| | AMR | 0.533 | -0.098 | 1.165 | 0.175 |
| | CSA | 0.128 | -0.008 | 0.264 | 0.553 |
| | EAS | -0.105 | -0.400 | 0.191 | 0.662 |
| | EUR | -0.003 | -0.020 | 0.014 | 0.891 |
| | MID | 0.319 | -0.690 | 1.328 | 0.783 |
| F50-59, Behavioural syndromes | AFR | 0.022 | -0.109 | 0.154 | 0.891 |
| | AMR | 0.573 | -0.074 | 1.220 | 0.175 |
| | CSA | 0.014 | -0.121 | 0.149 | 0.870 |
| | EAS | -0.336 | -0.656 | -0.016 | 0.357 |
| | EUR | 0.000 | -0.017 | 0.017 | 0.992 |
| | MID | 0.875 | -0.202 | 1.953 | 0.728 |
| F70-79, Mental retardation | AFR | -0.030 | -0.181 | 0.122 | 0.891 |
| | AMR | -0.325 | -1.077 | 0.427 | 0.507 |
| | CSA | 0.052 | -0.088 | 0.192 | 0.733 |
| | EAS | 0.024 | -0.277 | 0.325 | 0.878 |
| | EUR | 0.007 | -0.010 | 0.023 | 0.794 |
| | MID | -0.210 | -1.140 | 0.720 | 0.783 |
| F80-89, Developmental | AFR | 0.023 | -0.126 | 0.172 | 0.891 |
| | AMR | 0.075 | -0.539 | 0.690 | 0.867 |
| | CSA | 0.011 | -0.117 | 0.139 | 0.870 |
| | EAS | 0.042 | -0.249 | 0.333 | 0.874 |
| | EUR | 0.008 | -0.009 | 0.024 | 0.794 |
| | MID | -0.313 | -1.125 | 0.498 | 0.783 |
| F90-98, Child & adolescent | AFR | -0.066 | -0.216 | 0.084 | 0.891 |
| | AMR | 0.628 | -0.041 | 1.296 | 0.175 |
| | CSA | 0.040 | -0.089 | 0.169 | 0.733 |
| | EAS | -0.149 | -0.423 | 0.125 | 0.637 |
| | EUR | 0.004 | -0.012 | 0.020 | 0.891 |
| | MID | 0.308 | -0.771 | 1.388 | 0.783 |

*Note:* AFR = African; AMR = Admixed American; EAS = East Asian; EUR = European; CSA = Central and South Asian; MID = Middle Eastern. Models were adjusted for chronological age, sex, assessment centre, batch number (except for MID), the first six genetic principal components and fasting time. Cells highlighted in grey and blue correspond to statistically significant associations (nominally and after multiple testing corrections, respectively). *P*-values shown are corrected for multiple testing using the Benjamini–Hochberg procedure (separately within each population). *N* = 3426 (AFR); *N* = 278 (AMR); *N* = 3916 (CSA); *N* = 1012 (EAS); *N* = 210,755 (EUR); *N* = 94 (MID).

## 17. MileAge delta and polygenic scores for individual disorders

**Table S15.** MileAge delta and polygenic scores for individual disorders across ancestries

| Polygenic score | *β* | 95% CI | | *p* |
|---|---|---|---|---|
| F10, Alcohol use | -0.010 | -0.027 | 0.007 | 0.448 |
| F11, Opioid use | -0.009 | -0.026 | 0.009 | 0.499 |
| F12, Cannabis use | 0.001 | -0.015 | 0.018 | 0.922 |
| F13, Sedative use | -0.002 | -0.019 | 0.014 | 0.886 |
| F15, Stimulant use | -0.003 | -0.020 | 0.014 | 0.886 |
| F17, Tobacco use | -0.034 | -0.051 | -0.017 | <0.001 |
| F19, Multi-substance use | 0.000 | -0.017 | 0.017 | 0.991 |
| Schizophrenia | -0.009 | -0.024 | 0.007 | 0.448 |
| F22, Delusional | -0.019 | -0.035 | -0.004 | 0.053 |
| F23, Acute psychosis | -0.040 | -0.056 | -0.023 | <0.001 |
| F25, Schizoaffective | -0.023 | -0.038 | -0.007 | 0.022 |
| F29, Psychosis (unspecific) | -0.027 | -0.043 | -0.011 | 0.008 |
| F30, Mania | -0.003 | -0.020 | 0.014 | 0.886 |
| Bipolar | -0.002 | -0.017 | 0.013 | 0.886 |
| F32, Depressive episode | 0.011 | -0.005 | 0.028 | 0.417 |
| F33, Recurrent depression | 0.004 | -0.012 | 0.021 | 0.799 |
| Depression | 0.020 | 0.004 | 0.035 | 0.048 |
| F34, Persistent mood | -0.012 | -0.028 | 0.005 | 0.403 |
| F39, Mood (unspecific) | 0.013 | -0.003 | 0.030 | 0.357 |
| F40, Phobic | -0.011 | -0.029 | 0.006 | 0.435 |
| F41, Anxiety | -0.009 | -0.024 | 0.007 | 0.448 |
| F42, Obsessive-compulsive | -0.035 | -0.052 | -0.019 | <0.001 |
| F43, Stress-related | -0.009 | -0.026 | 0.008 | 0.448 |
| F44, Dissociative | 0.004 | -0.012 | 0.021 | 0.799 |
| F45, Somatoform | -0.012 | -0.028 | 0.005 | 0.403 |
| F48, Other neurotic | -0.009 | -0.025 | 0.006 | 0.448 |
| F50, Eating | 0.001 | -0.016 | 0.017 | 0.964 |
| Anorexia | -0.023 | -0.038 | -0.008 | 0.013 |
| F51, Sleep | -0.007 | -0.024 | 0.009 | 0.538 |
| F60, Personality | -0.013 | -0.030 | 0.004 | 0.357 |
| F80, Speech and language | 0.016 | -0.001 | 0.033 | 0.194 |
| F81, Scholastic skills | 0.006 | -0.010 | 0.022 | 0.618 |
| Autism | 0.026 | 0.010 | 0.042 | 0.009 |
| ADHD | 0.047 | 0.031 | 0.063 | <0.001 |
| F93, Childhood emotional | 0.001 | -0.015 | 0.017 | 0.922 |
| F98, Child or adolescent (other) | 0.008 | -0.008 | 0.023 | 0.518 |
| F99, Unspecified | 0.009 | -0.007 | 0.025 | 0.448 |

*Note:* ADHD = attention-deficit/hyperactivity disorder; CI = confidence interval. Models were adjusted for chronological age, sex, assessment centre, batch number, the first six genetic principal components and fasting time. Cells highlighted in grey and blue correspond to statistically significant associations (nominally and after multiple testing corrections, respectively). *P*-values shown are corrected for multiple testing using the Benjamini–Hochberg procedure. *N* = 219,494.

**Table S16.** Ancestry-specific associations between MileAge delta and polygenic scores for individual disorders

| Polygenic score | Population | *β* | 95% CI | | *p* |
|---|---|---|---|---|---|
| F10, Alcohol use | AFR | 0.071 | -0.071 | 0.213 | 0.817 |
| | AMR | -0.842 | -1.635 | -0.049 | 0.422 |
| | CSA | 0.000 | -0.132 | 0.133 | 0.995 |
| | EAS | -0.246 | -0.537 | 0.046 | 0.637 |
| | EUR | -0.008 | -0.026 | 0.009 | 0.488 |
| | MID | 0.906 | -0.173 | 1.985 | 0.520 |
| F11, Opioid use | AFR | 0.071 | -0.086 | 0.229 | 0.817 |
| | AMR | 0.157 | -0.526 | 0.840 | 0.777 |
| | CSA | -0.064 | -0.196 | 0.068 | 0.608 |
| | EAS | -0.017 | -0.306 | 0.271 | 0.963 |
| | EUR | -0.009 | -0.026 | 0.009 | 0.488 |
| | MID | -0.047 | -1.112 | 1.017 | 0.935 |
| F12, Cannabis use | AFR | 0.049 | -0.092 | 0.189 | 0.817 |
| | AMR | 0.068 | -0.556 | 0.692 | 0.907 |
| | CSA | 0.117 | -0.011 | 0.245 | 0.557 |
| | EAS | -0.054 | -0.338 | 0.230 | 0.957 |
| | EUR | -0.002 | -0.019 | 0.015 | 0.943 |
| | MID | -0.282 | -1.236 | 0.672 | 0.854 |
| F13, Sedative use | AFR | -0.105 | -0.242 | 0.033 | 0.817 |
| | AMR | 0.327 | -0.350 | 1.003 | 0.610 |
| | CSA | -0.065 | -0.188 | 0.058 | 0.608 |
| | EAS | 0.115 | -0.155 | 0.385 | 0.957 |
| | EUR | 0.000 | -0.016 | 0.016 | 0.992 |
| | MID | -0.588 | -1.693 | 0.517 | 0.753 |
| F15, Stimulant use | AFR | -0.018 | -0.154 | 0.118 | 0.900 |
| | AMR | -0.266 | -1.366 | 0.834 | 0.777 |
| | CSA | -0.102 | -0.236 | 0.032 | 0.557 |
| | EAS | -0.054 | -0.353 | 0.245 | 0.957 |
| | EUR | 0.001 | -0.016 | 0.019 | 0.978 |
| | MID | 0.681 | -0.237 | 1.600 | 0.664 |
| F17, Tobacco use | AFR | -0.050 | -0.184 | 0.084 | 0.817 |
| | AMR | -0.522 | -1.112 | 0.068 | 0.422 |
| | CSA | -0.009 | -0.138 | 0.121 | 0.995 |
| | EAS | -0.014 | -0.309 | 0.281 | 0.963 |
| | EUR | -0.034 | -0.051 | -0.017 | <0.001 |
| | MID | -0.147 | -0.996 | 0.702 | 0.872 |
| F19, Multi-substance use | AFR | 0.029 | -0.107 | 0.164 | 0.887 |
| | AMR | 0.284 | -0.635 | 1.203 | 0.746 |
| | CSA | 0.035 | -0.098 | 0.167 | 0.898 |
| | EAS | -0.245 | -0.539 | 0.050 | 0.637 |
| | EUR | -0.001 | -0.018 | 0.017 | 0.980 |
| | MID | -0.261 | -1.145 | 0.623 | 0.854 |
| Schizophrenia | AFR | -0.027 | -0.182 | 0.127 | 0.894 |
| | AMR | 0.466 | -0.095 | 1.027 | 0.422 |
| | CSA | 0.029 | -0.108 | 0.166 | 0.928 |
| | EAS | 0.074 | -0.221 | 0.369 | 0.957 |
| | EUR | -0.012 | -0.028 | 0.004 | 0.292 |
| | MID | 0.059 | -1.103 | 1.220 | 0.935 |
| F22, Delusional | AFR | 0.056 | -0.095 | 0.206 | 0.817 |
| | AMR | 0.242 | -0.433 | 0.918 | 0.739 |
| | CSA | -0.004 | -0.146 | 0.139 | 0.995 |
| | EAS | 0.261 | -0.030 | 0.553 | 0.637 |
| | EUR | -0.023 | -0.038 | -0.007 | 0.020 |
| | MID | 0.571 | -0.362 | 1.505 | 0.743 |
| F23, Acute psychosis | AFR | 0.005 | -0.138 | 0.147 | 0.974 |
| | AMR | -0.635 | -1.300 | 0.030 | 0.422 |
| | CSA | -0.009 | -0.140 | 0.123 | 0.995 |
| | EAS | 0.133 | -0.160 | 0.426 | 0.957 |
| | EUR | -0.042 | -0.059 | -0.025 | <0.001 |
| | MID | -0.222 | -1.200 | 0.755 | 0.854 |
| F25, Schizoaffective | AFR | 0.010 | -0.129 | 0.149 | 0.956 |
| | AMR | -0.431 | -1.110 | 0.249 | 0.514 |
| | CSA | -0.020 | -0.145 | 0.105 | 0.928 |
| | EAS | 0.124 | -0.164 | 0.412 | 0.957 |

| | | | | | |
|---|---|---|---|---|---|
| | EUR | -0.024 | -0.040 | -0.008 | 0.020 |
| | MID | 0.258 | -0.875 | 1.391 | 0.854 |
| F29, Psychosis (unspecific) | AFR | 0.009 | -0.137 | 0.155 | 0.956 |
| | AMR | 0.254 | -0.632 | 1.141 | 0.746 |
| | CSA | -0.092 | -0.218 | 0.034 | 0.557 |
| | EAS | 0.055 | -0.246 | 0.356 | 0.957 |
| | EUR | -0.028 | -0.045 | -0.011 | 0.006 |
| | MID | 1.058 | -0.111 | 2.228 | 0.465 |
| F30, Mania | AFR | 0.053 | -0.095 | 0.201 | 0.817 |
| | AMR | 0.172 | -0.448 | 0.792 | 0.746 |
| | CSA | 0.030 | -0.099 | 0.160 | 0.920 |
| | EAS | 0.050 | -0.231 | 0.331 | 0.957 |
| | EUR | -0.006 | -0.023 | 0.012 | 0.682 |
| | MID | -0.305 | -1.280 | 0.670 | 0.854 |
| Bipolar | AFR | 0.060 | -0.102 | 0.223 | 0.817 |
| | AMR | 0.510 | -0.198 | 1.218 | 0.446 |
| | CSA | -0.003 | -0.130 | 0.124 | 0.995 |
| | EAS | 0.310 | 0.024 | 0.597 | 0.637 |
| | EUR | -0.005 | -0.021 | 0.010 | 0.682 |
| | MID | 0.343 | -0.764 | 1.450 | 0.854 |
| F32, Depressive episode | AFR | -0.045 | -0.190 | 0.099 | 0.817 |
| | AMR | 0.464 | -0.144 | 1.073 | 0.446 |
| | CSA | 0.097 | -0.039 | 0.232 | 0.557 |
| | EAS | -0.169 | -0.457 | 0.120 | 0.957 |
| | EUR | 0.011 | -0.006 | 0.027 | 0.392 |
| | MID | 0.150 | -0.891 | 1.192 | 0.879 |
| F33, Recurrent depression | AFR | -0.002 | -0.146 | 0.141 | 0.975 |
| | AMR | 0.581 | -0.099 | 1.260 | 0.422 |
| | CSA | 0.035 | -0.096 | 0.166 | 0.898 |
| | EAS | 0.040 | -0.267 | 0.348 | 0.957 |
| | EUR | 0.004 | -0.013 | 0.020 | 0.829 |
| | MID | 0.279 | -0.542 | 1.100 | 0.854 |
| Depression | AFR | 0.025 | -0.127 | 0.176 | 0.894 |
| | AMR | 0.503 | -0.176 | 1.183 | 0.446 |
| | CSA | 0.081 | -0.058 | 0.220 | 0.557 |
| | EAS | -0.069 | -0.355 | 0.216 | 0.957 |
| | EUR | 0.019 | 0.003 | 0.034 | 0.078 |
| | MID | 0.891 | -0.073 | 1.854 | 0.465 |
| F34, Persistent mood | AFR | -0.083 | -0.222 | 0.055 | 0.817 |
| | AMR | 0.308 | -0.328 | 0.944 | 0.610 |
| | CSA | -0.094 | -0.223 | 0.035 | 0.557 |
| | EAS | 0.012 | -0.259 | 0.283 | 0.963 |
| | EUR | -0.010 | -0.027 | 0.007 | 0.417 |
| | MID | -0.108 | -0.988 | 0.772 | 0.879 |
| F39, Mood (unspecific) | AFR | 0.032 | -0.116 | 0.181 | 0.887 |
| | AMR | 0.027 | -0.548 | 0.603 | 0.925 |
| | CSA | 0.066 | -0.064 | 0.197 | 0.608 |
| | EAS | 0.243 | -0.038 | 0.523 | 0.637 |
| | EUR | 0.010 | -0.007 | 0.027 | 0.417 |
| | MID | -0.463 | -1.587 | 0.661 | 0.854 |
| F40, Phobic | AFR | 0.082 | -0.059 | 0.224 | 0.817 |
| | AMR | 0.493 | -0.249 | 1.235 | 0.506 |
| | CSA | 0.082 | -0.050 | 0.214 | 0.557 |
| | EAS | -0.049 | -0.355 | 0.257 | 0.957 |
| | EUR | -0.015 | -0.033 | 0.002 | 0.239 |
| | MID | -0.048 | -1.235 | 1.138 | 0.935 |
| F41, Anxiety | AFR | 0.018 | -0.123 | 0.159 | 0.900 |
| | AMR | 0.601 | -0.102 | 1.303 | 0.422 |
| | CSA | 0.086 | -0.049 | 0.220 | 0.557 |
| | EAS | -0.046 | -0.337 | 0.246 | 0.957 |
| | EUR | -0.011 | -0.027 | 0.005 | 0.346 |
| | MID | -0.203 | -1.349 | 0.943 | 0.872 |
| F42, Obsessive-compulsive | AFR | -0.042 | -0.179 | 0.095 | 0.817 |
| | AMR | -0.158 | -0.696 | 0.381 | 0.746 |
| | CSA | -0.076 | -0.202 | 0.050 | 0.557 |
| | EAS | -0.035 | -0.324 | 0.254 | 0.957 |

| | | | | | |
|---|---|---|---|---|---|
| | EUR | -0.035 | -0.051 | -0.018 | <0.001 |
| | MID | -0.299 | -1.207 | 0.609 | 0.854 |
| F43, Stress-related | AFR | 0.118 | -0.032 | 0.267 | 0.817 |
| | AMR | -0.252 | -0.851 | 0.348 | 0.687 |
| | CSA | -0.078 | -0.204 | 0.048 | 0.557 |
| | EAS | -0.168 | -0.477 | 0.140 | 0.957 |
| | EUR | -0.010 | -0.027 | 0.007 | 0.435 |
| | MID | -0.547 | -1.512 | 0.417 | 0.743 |
| F44, Dissociative | AFR | 0.062 | -0.085 | 0.209 | 0.817 |
| | AMR | -0.435 | -1.027 | 0.158 | 0.446 |
| | CSA | 0.082 | -0.050 | 0.214 | 0.557 |
| | EAS | -0.244 | -0.558 | 0.071 | 0.683 |
| | EUR | 0.003 | -0.013 | 0.020 | 0.829 |
| | MID | -0.215 | -1.213 | 0.784 | 0.854 |
| F45, Somatoform | AFR | 0.062 | -0.082 | 0.206 | 0.817 |
| | AMR | 0.033 | -0.595 | 0.660 | 0.925 |
| | CSA | 0.004 | -0.127 | 0.135 | 0.995 |
| | EAS | 0.161 | -0.153 | 0.474 | 0.957 |
| | EUR | -0.015 | -0.032 | 0.002 | 0.217 |
| | MID | 0.237 | -0.650 | 1.125 | 0.854 |
| F48, Other neurotic | AFR | 0.098 | -0.039 | 0.235 | 0.817 |
| | AMR | 0.777 | 0.122 | 1.432 | 0.422 |
| | CSA | 0.022 | -0.101 | 0.145 | 0.928 |
| | EAS | 0.254 | -0.018 | 0.527 | 0.637 |
| | EUR | -0.014 | -0.030 | 0.003 | 0.242 |
| | MID | -0.199 | -1.101 | 0.703 | 0.854 |
| F50, Eating | AFR | 0.045 | -0.097 | 0.187 | 0.817 |
| | AMR | 0.291 | -0.317 | 0.900 | 0.610 |
| | CSA | -0.062 | -0.192 | 0.067 | 0.608 |
| | EAS | 0.099 | -0.183 | 0.380 | 0.957 |
| | EUR | 0.001 | -0.015 | 0.018 | 0.978 |
| | MID | 1.052 | -0.027 | 2.131 | 0.465 |
| Anorexia | AFR | -0.095 | -0.261 | 0.071 | 0.817 |
| | AMR | 0.239 | -0.565 | 1.043 | 0.746 |
| | CSA | -0.053 | -0.190 | 0.084 | 0.719 |
| | EAS | -0.089 | -0.380 | 0.203 | 0.957 |
| | EUR | -0.022 | -0.037 | -0.007 | 0.020 |
| | MID | -0.165 | -1.483 | 1.152 | 0.879 |
| F51, Sleep | AFR | 0.042 | -0.096 | 0.180 | 0.817 |
| | AMR | 0.566 | -0.103 | 1.235 | 0.422 |
| | CSA | 0.050 | -0.077 | 0.176 | 0.719 |
| | EAS | -0.106 | -0.394 | 0.183 | 0.957 |
| | EUR | -0.009 | -0.026 | 0.007 | 0.435 |
| | MID | 1.126 | 0.262 | 1.989 | 0.409 |
| F60, Personality | AFR | 0.029 | -0.110 | 0.167 | 0.887 |
| | AMR | 0.843 | 0.054 | 1.632 | 0.422 |
| | CSA | 0.078 | -0.056 | 0.212 | 0.557 |
| | EAS | -0.042 | -0.329 | 0.245 | 0.957 |
| | EUR | -0.017 | -0.034 | 0.001 | 0.182 |
| | MID | 0.663 | -0.396 | 1.721 | 0.743 |
| F80, Speech and language | AFR | -0.045 | -0.191 | 0.102 | 0.817 |
| | AMR | -0.382 | -1.035 | 0.272 | 0.514 |
| | CSA | -0.089 | -0.220 | 0.041 | 0.557 |
| | EAS | -0.136 | -0.415 | 0.142 | 0.957 |
| | EUR | 0.019 | 0.002 | 0.036 | 0.093 |
| | MID | -0.614 | -1.664 | 0.437 | 0.743 |
| F81, Scholastic skills | AFR | -0.030 | -0.182 | 0.121 | 0.887 |
| | AMR | 0.241 | -0.405 | 0.887 | 0.739 |
| | CSA | 0.122 | -0.003 | 0.247 | 0.557 |
| | EAS | 0.038 | -0.259 | 0.336 | 0.957 |
| | EUR | 0.004 | -0.013 | 0.020 | 0.829 |
| | MID | -1.301 | -2.409 | -0.193 | 0.409 |
| Autism | AFR | -0.106 | -0.251 | 0.040 | 0.817 |
| | AMR | -0.064 | -0.724 | 0.595 | 0.907 |
| | CSA | -0.001 | -0.129 | 0.127 | 0.995 |
| | EAS | -0.089 | -0.379 | 0.201 | 0.957 |

| | | | | | |
|---|---|---|---|---|---|
| | EUR | 0.028 | 0.012 | 0.044 | 0.006 |
| | MID | 0.658 | -0.342 | 1.658 | 0.743 |
| ADHD | AFR | 0.211 | 0.076 | 0.346 | 0.079 |
| | AMR | 0.060 | -0.605 | 0.725 | 0.907 |
| | CSA | 0.109 | -0.022 | 0.240 | 0.557 |
| | EAS | -0.073 | -0.378 | 0.232 | 0.957 |
| | EUR | 0.042 | 0.026 | 0.059 | <0.001 |
| | MID | 0.389 | -0.635 | 1.413 | 0.854 |
| F93, Childhood emotional | AFR | 0.050 | -0.098 | 0.197 | 0.817 |
| | AMR | 0.355 | -0.249 | 0.959 | 0.514 |
| | CSA | 0.116 | -0.012 | 0.243 | 0.557 |
| | EAS | -0.011 | -0.285 | 0.263 | 0.963 |
| | EUR | -0.001 | -0.017 | 0.016 | 0.980 |
| | MID | -1.025 | -2.007 | -0.043 | 0.465 |
| F98, Child or adolescent (other) | AFR | -0.080 | -0.214 | 0.053 | 0.817 |
| | AMR | 0.461 | -0.282 | 1.203 | 0.514 |
| | CSA | -0.088 | -0.214 | 0.037 | 0.557 |
| | EAS | -0.007 | -0.305 | 0.291 | 0.965 |
| | EUR | 0.012 | -0.004 | 0.028 | 0.292 |
| | MID | 0.251 | -0.891 | 1.393 | 0.854 |
| F99, Unspecified | AFR | -0.054 | -0.195 | 0.086 | 0.817 |
| | AMR | -0.137 | -0.834 | 0.561 | 0.809 |
| | CSA | 0.024 | -0.108 | 0.157 | 0.928 |
| | EAS | 0.029 | -0.236 | 0.295 | 0.957 |
| | EUR | 0.009 | -0.008 | 0.025 | 0.456 |
| | MID | 0.658 | -0.614 | 1.930 | 0.753 |

*Note:* AFR = African; AMR = Admixed American; EAS = East Asian; EUR = European; CSA = Central and South Asian; MID = Middle Eastern. Models were adjusted for chronological age, sex, assessment centre, batch number (except for MID), the first six genetic principal components and fasting time. Cells highlighted in grey and blue correspond to statistically significant associations (nominally and after multiple testing corrections, respectively). *P*-values shown are corrected for multiple testing using the Benjamini–Hochberg procedure (separately within each population). *N* = 3426 (AFR); *N* = 278 (AMR); *N* = 3916 (CSA); *N* = 1012 (EAS); *N* = 210,755 (EUR); *N* = 94 (MID).

## 18. MileAge delta and individual two-digit ICD-10 codes; excluding self-report

**Table S17.** MileAge delta and mental/behavioural disorders; excluding self-report diagnoses

| | | Model 1 (adj. age and sex) | | | | Model 2 (full adjustment) | | | |
|---|---|---|---|---|---|---|---|---|---|
| ICD-10 | *N* | *β* | 95% CI | | *p* | *β* | 95% CI | | *p* |
| None | 186688 | Reference | | | | Reference | | | |
| F10, Alcohol use | 1303 | 0.705 | 0.500 | 0.910 | <0.001 | 0.603 | 0.397 | 0.808 | <0.001 |
| F11, Opioid use | 46 | 1.113 | 0.028 | 2.199 | 0.157 | 0.907 | -0.178 | 1.992 | 0.285 |
| F12, Cannabis use | 46 | -0.914 | -1.999 | 0.172 | 0.285 | -1.108 | -2.193 | -0.023 | 0.157 |
| F13, Sedative use | 68 | -0.394 | -1.287 | 0.498 | 0.571 | -0.479 | -1.371 | 0.413 | 0.537 |
| F14, Cocaine use | 10 | 0.965 | -1.363 | 3.293 | 0.595 | 0.710 | -1.616 | 3.036 | 0.707 |
| F15, Stimulant use | 14 | 0.942 | -1.026 | 2.909 | 0.569 | 0.739 | -1.227 | 2.705 | 0.615 |
| F16, Hallucinogen use | 12 | 3.758 | 1.633 | 5.883 | 0.006 | 3.630 | 1.507 | 5.753 | 0.008 |
| F17, Tobacco use | 9715 | 0.409 | 0.332 | 0.485 | <0.001 | 0.373 | 0.296 | 0.450 | <0.001 |
| F19, Multi-substance use | 61 | 0.598 | -0.344 | 1.541 | 0.497 | 0.440 | -0.502 | 1.382 | 0.569 |
| F20, Schizophrenia | 150 | 0.453 | -0.148 | 1.055 | 0.379 | 0.236 | -0.366 | 0.837 | 0.615 |
| F22, Delusional | 88 | 0.956 | 0.171 | 1.741 | 0.082 | 0.804 | 0.020 | 1.589 | 0.157 |
| F23, Acute psychosis | 66 | 0.044 | -0.862 | 0.950 | 0.956 | -0.069 | -0.974 | 0.837 | 0.937 |
| F25, Schizoaffective | 85 | 0.905 | 0.106 | 1.703 | 0.113 | 0.729 | -0.069 | 1.527 | 0.236 |
| F29, Psychosis (unspecific) | 83 | 0.541 | -0.267 | 1.349 | 0.461 | 0.366 | -0.442 | 1.174 | 0.569 |
| F30, Mania | 124 | 0.494 | -0.167 | 1.155 | 0.379 | 0.408 | -0.252 | 1.069 | 0.497 |
| F31, Bipolar | 162 | 0.635 | 0.057 | 1.214 | 0.123 | 0.535 | -0.044 | 1.113 | 0.233 |
| F32, Depressive episode | 5425 | 0.288 | 0.186 | 0.389 | <0.001 | 0.243 | 0.141 | 0.345 | <0.001 |
| F33, Recurrent depression | 1211 | 0.327 | 0.115 | 0.539 | 0.017 | 0.268 | 0.056 | 0.480 | 0.070 |
| F34, Persistent mood | 306 | 0.247 | -0.174 | 0.668 | 0.501 | 0.190 | -0.231 | 0.611 | 0.569 |
| F38, Mood (other) | 36 | 0.300 | -0.927 | 1.527 | 0.748 | 0.237 | -0.989 | 1.462 | 0.814 |
| F39, Mood (unspecific) | 282 | 0.201 | -0.238 | 0.640 | 0.569 | 0.171 | -0.268 | 0.609 | 0.615 |
| F40, Phobic | 717 | 0.151 | -0.125 | 0.426 | 0.531 | 0.119 | -0.156 | 0.395 | 0.575 |
| F41, Anxiety | 4772 | 0.206 | 0.098 | 0.314 | 0.002 | 0.170 | 0.062 | 0.278 | 0.017 |
| F42, Obsessive-compulsive | 214 | -0.698 | -1.201 | -0.194 | 0.037 | -0.776 | -1.279 | -0.273 | 0.017 |
| F43, Stress-related | 4094 | 0.193 | 0.077 | 0.310 | 0.010 | 0.167 | 0.051 | 0.283 | 0.030 |
| F44, Dissociative | 79 | 0.374 | -0.454 | 1.203 | 0.569 | 0.317 | -0.510 | 1.145 | 0.615 |
| F45, Somatoform | 843 | 0.021 | -0.233 | 0.276 | 0.937 | 0.017 | -0.237 | 0.271 | 0.937 |
| F48, Other neurotic | 108 | -0.318 | -1.026 | 0.391 | 0.569 | -0.348 | -1.056 | 0.360 | 0.569 |
| F50, Eating | 182 | -0.245 | -0.792 | 0.301 | 0.569 | -0.257 | -0.803 | 0.289 | 0.569 |
| F51, Sleep | 153 | 0.367 | -0.228 | 0.963 | 0.497 | 0.362 | -0.232 | 0.957 | 0.498 |
| F52, Sexual dysfunction | 632 | -0.008 | -0.301 | 0.286 | 0.971 | -0.012 | -0.305 | 0.281 | 0.959 |
| F53, Puerperal psychosis | 268 | -0.393 | -0.843 | 0.058 | 0.272 | -0.383 | -0.833 | 0.067 | 0.285 |
| F60, Personality | 180 | 0.805 | 0.256 | 1.354 | 0.026 | 0.667 | 0.118 | 1.216 | 0.082 |
| F63, Impulse | 16 | 1.142 | -0.698 | 2.982 | 0.497 | 1.083 | -0.755 | 2.922 | 0.501 |
| F66, Sexual development | 155 | -0.333 | -0.924 | 0.259 | 0.524 | -0.351 | -0.942 | 0.240 | 0.501 |
| F69, Personality (unspecific) | 64 | -0.003 | -0.923 | 0.918 | 0.996 | -0.089 | -1.009 | 0.830 | 0.932 |
| F80, Speech and language | 31 | 0.441 | -0.881 | 1.763 | 0.670 | 0.375 | -0.946 | 1.696 | 0.712 |
| F81, Scholastic skills | 60 | 0.355 | -0.596 | 1.305 | 0.615 | 0.236 | -0.714 | 1.186 | 0.748 |
| F84, Pervasive developmental | 18 | 0.497 | -1.238 | 2.232 | 0.712 | 0.357 | -1.376 | 2.091 | 0.802 |
| F90, ADHD | 10 | -0.324 | -2.652 | 2.003 | 0.883 | -0.373 | -2.699 | 1.952 | 0.858 |
| F91, Conduct | 43 | 1.314 | 0.192 | 2.437 | 0.098 | 1.238 | 0.117 | 2.360 | 0.123 |
| F93, Childhood emotional | 11 | 0.650 | -1.570 | 2.869 | 0.712 | 0.594 | -1.624 | 2.811 | 0.729 |
| F95, Tic | 11 | 1.550 | -0.669 | 3.770 | 0.431 | 1.544 | -0.673 | 3.761 | 0.431 |
| F98, Child or adolescent (other) | 45 | -0.580 | -1.678 | 0.517 | 0.540 | -0.612 | -1.709 | 0.484 | 0.524 |
| F99, Unspecified | 86 | 0.085 | -0.708 | 0.879 | 0.925 | -0.059 | -0.852 | 0.735 | 0.937 |

*Note:* ICD-10 = International Classification of Diseases, 10th Revision; CI = confidence interval. Model 1–adjusted for chronological age and sex; Model 2–adjusted for chronological age, sex, ethnicity, cohabitation with spouse/partner, highest educational/professional qualification, annual gross household income, Townsend deprivation index and fasting time. For diagnoses printed in grey, there were no differences in sample size compared to the primary analysis. Cells highlighted in grey and blue correspond to statistically significant associations (nominally and after multiple testing corrections, respectively). *P*-values shown are corrected for multiple testing using the Benjamini–Hochberg procedure.

## 19. MileAge delta and groups of two-digit ICD-10 codes; primary care linkage

**Table S18.** MileAge delta and mental/behavioural disorders; primary care linkage

| | | Model 1 (adj. age and sex) | | | | Model 2 (full adjustment) | | | |
|---|---|---|---|---|---|---|---|---|---|
| ICD-10 | | *β* | 95% CI | | *p* | *β* | 95% CI | | *p* |
| None | 75576 | Reference | | | | Reference | | | |
| F10-99, Any disorder | 27147 | 0.226 | 0.174 | 0.279 | <0.001 | 0.197 | 0.144 | 0.250 | <0.001 |
| F10-19, Substance use | 10265 | 0.379 | 0.302 | 0.457 | <0.001 | 0.352 | 0.274 | 0.431 | <0.001 |
| F20-29, Psychosis | 319 | 0.716 | 0.304 | 1.128 | 0.002 | 0.597 | 0.183 | 1.010 | 0.010 |
| F30-39, Affective | 11834 | 0.241 | 0.168 | 0.314 | <0.001 | 0.205 | 0.131 | 0.279 | <0.001 |
| F40-48, Neurotic | 10900 | 0.136 | 0.060 | 0.211 | 0.001 | 0.111 | 0.035 | 0.187 | 0.010 |
| F50-59, Behavioural syndromes | 1399 | -0.243 | -0.442 | -0.045 | 0.030 | -0.245 | -0.443 | -0.046 | 0.030 |
| F60-69, Personality & behaviour | 368 | 0.154 | -0.230 | 0.538 | 0.500 | 0.109 | -0.275 | 0.493 | 0.636 |
| F70-79, Mental retardation | 11 | 1.559 | -0.656 | 3.773 | 0.231 | 1.392 | -0.822 | 3.607 | 0.266 |
| F80-89, Developmental | 90 | 0.593 | -0.182 | 1.368 | 0.210 | 0.506 | -0.269 | 1.282 | 0.260 |
| F90-98, Child & adolescent | 118 | 0.542 | -0.135 | 1.218 | 0.197 | 0.498 | -0.178 | 1.174 | 0.218 |
| F99, Unspecified | 56 | 0.180 | -0.802 | 1.161 | 0.754 | 0.085 | -0.897 | 1.066 | 0.865 |

*Note:* ICD-10 = International Classification of Diseases, 10th Revision; CI = confidence interval. Model 1–adjusted for chronological age and sex; Model 2–adjusted for chronological age, sex, ethnicity, cohabitation with spouse/partner, highest educational/professional qualification, annual gross household income, Townsend deprivation index and fasting time. Cells highlighted in grey and blue correspond to statistically significant associations (nominally and after multiple testing corrections, respectively). *P*-values shown are corrected for multiple testing using the Benjamini–Hochberg procedure.

## 20. MileAge delta and individual two-digit ICD-10 codes; primary care linkage

**Table S19.** MileAge delta and mental/behavioural disorders; primary care linkage

| | | Model 1 (adj. age and sex) | | | | Model 2 (full adjustment) | | | |
|---|---|---|---|---|---|---|---|---|---|
| ICD-10 | *N* | *β* | 95% CI | | *p* | *β* | 95% CI | | *p* |
| None | 75576 | Reference | | | | Reference | | | |
| F10, Alcohol use | 839 | 0.612 | 0.357 | 0.868 | <0.001 | 0.543 | 0.286 | 0.800 | <0.001 |
| F11, Opioid use | 29 | 1.025 | -0.339 | 2.389 | 0.417 | 0.885 | -0.479 | 2.250 | 0.461 |
| F12, Cannabis use | 30 | -0.826 | -2.168 | 0.515 | 0.489 | -0.913 | -2.256 | 0.429 | 0.436 |
| F13, Sedative use | 66 | -0.438 | -1.342 | 0.467 | 0.602 | -0.499 | -1.403 | 0.406 | 0.519 |
| F14, Cocaine use | | *N* insufficient | | | | | | | |
| F15, Stimulant use | 11 | 0.982 | -1.232 | 3.197 | 0.642 | 0.795 | -1.419 | 3.009 | 0.690 |
| F16, Hallucinogen use | | *N* insufficient | | | | | | | |
| F17, Tobacco use | 9556 | 0.365 | 0.285 | 0.445 | <0.001 | 0.342 | 0.261 | 0.423 | <0.001 |
| F19, Multi-substance use | 59 | 0.676 | -0.281 | 1.633 | 0.436 | 0.561 | -0.396 | 1.518 | 0.513 |
| F20, Schizophrenia | 182 | 1.020 | 0.474 | 1.565 | 0.003 | 0.866 | 0.319 | 1.413 | 0.021 |
| F22, Delusional | 66 | 0.909 | 0.005 | 1.814 | 0.168 | 0.797 | -0.108 | 1.702 | 0.278 |
| F23, Acute psychosis | 43 | -0.307 | -1.428 | 0.813 | 0.758 | -0.376 | -1.496 | 0.744 | 0.708 |
| F25, Schizoaffective | 52 | 0.806 | -0.213 | 1.825 | 0.385 | 0.700 | -0.319 | 1.719 | 0.436 |
| F29, Psychosis (unspecific) | 66 | 0.293 | -0.612 | 1.198 | 0.717 | 0.157 | -0.748 | 1.062 | 0.833 |
| F30, Mania | 99 | 0.374 | -0.364 | 1.113 | 0.574 | 0.321 | -0.418 | 1.059 | 0.642 |
| F31, Bipolar | 362 | 0.168 | -0.219 | 0.555 | 0.642 | 0.099 | -0.288 | 0.487 | 0.768 |
| F32, Depressive episode | 10933 | 0.253 | 0.178 | 0.329 | <0.001 | 0.217 | 0.140 | 0.293 | <0.001 |
| F33, Recurrent depression | 1072 | 0.286 | 0.060 | 0.512 | 0.075 | 0.240 | 0.014 | 0.466 | 0.148 |
| F34, Persistent mood | 292 | 0.175 | -0.256 | 0.605 | 0.676 | 0.124 | -0.307 | 0.555 | 0.756 |
| F38, Mood (other) | 36 | 0.273 | -0.952 | 1.497 | 0.802 | 0.214 | -1.010 | 1.438 | 0.833 |
| F39, Mood (unspecific) | 273 | 0.179 | -0.267 | 0.624 | 0.676 | 0.152 | -0.293 | 0.598 | 0.708 |
| F40, Phobic | 666 | 0.034 | -0.253 | 0.320 | 0.880 | 0.009 | -0.277 | 0.295 | 0.971 |
| F41, Anxiety | 6076 | 0.135 | 0.037 | 0.234 | 0.049 | 0.106 | 0.007 | 0.204 | 0.148 |
| F42, Obsessive-compulsive | 218 | -0.600 | -1.098 | -0.102 | 0.098 | -0.666 | -1.164 | -0.167 | 0.058 |
| F43, Stress-related | 4336 | 0.168 | 0.053 | 0.283 | 0.034 | 0.150 | 0.035 | 0.265 | 0.067 |
| F44, Dissociative | 57 | 0.236 | -0.737 | 1.209 | 0.780 | 0.175 | -0.798 | 1.148 | 0.833 |
| F45, Somatoform | 770 | -0.040 | -0.306 | 0.226 | 0.842 | -0.046 | -0.312 | 0.220 | 0.833 |
| F48, Other neurotic | 266 | -0.175 | -0.626 | 0.276 | 0.688 | -0.214 | -0.665 | 0.237 | 0.607 |
| F50, Eating | 266 | -0.464 | -0.916 | -0.012 | 0.159 | -0.479 | -0.931 | -0.027 | 0.148 |
| F51, Sleep | 153 | 0.331 | -0.263 | 0.926 | 0.519 | 0.325 | -0.269 | 0.919 | 0.519 |
| F52, Sexual dysfunction | 569 | -0.112 | -0.421 | 0.197 | 0.690 | -0.113 | -0.422 | 0.195 | 0.690 |
| F53, Puerperal psychosis | 430 | -0.530 | -0.886 | -0.174 | 0.034 | -0.519 | -0.875 | -0.163 | 0.034 |
| F60, Personality | 126 | 0.766 | 0.111 | 1.421 | 0.104 | 0.683 | 0.027 | 1.339 | 0.154 |
| F63, Impulse | 16 | 1.137 | -0.699 | 2.974 | 0.489 | 1.083 | -0.752 | 2.918 | 0.513 |
| F66, Sexual development | 153 | -0.396 | -0.990 | 0.199 | 0.446 | -0.413 | -1.007 | 0.181 | 0.436 |
| F69, Personality (unspecific) | 59 | -0.050 | -1.007 | 0.906 | 0.951 | -0.100 | -1.056 | 0.856 | 0.890 |
| F80, Speech and language | 25 | 0.016 | -1.453 | 1.485 | 0.983 | -0.027 | -1.496 | 1.441 | 0.982 |
| F81, Scholastic skills | 43 | 0.857 | -0.264 | 1.977 | 0.412 | 0.781 | -0.340 | 1.903 | 0.436 |
| F84, Pervasive developmental | 17 | 0.661 | -1.120 | 2.443 | 0.690 | 0.521 | -1.260 | 2.302 | 0.756 |
| F90, ADHD | 10 | -0.341 | -2.664 | 1.981 | 0.842 | -0.382 | -2.703 | 1.939 | 0.834 |
| F91, Conduct | 42 | 1.351 | 0.217 | 2.484 | 0.099 | 1.291 | 0.158 | 2.424 | 0.116 |
| F93, Childhood emotional | 11 | 0.625 | -1.590 | 2.839 | 0.756 | 0.577 | -1.636 | 2.790 | 0.768 |
| F95, Tic | 11 | 1.548 | -0.666 | 3.763 | 0.436 | 1.536 | -0.677 | 3.749 | 0.436 |
| F98, Child or adolescent (other) | 45 | -0.602 | -1.697 | 0.493 | 0.519 | -0.630 | -1.725 | 0.464 | 0.518 |
| F99, Unspecified | 56 | 0.180 | -0.802 | 1.161 | 0.833 | 0.085 | -0.897 | 1.066 | 0.908 |

*Note:* ICD-10 = International Classification of Diseases, 10th Revision; CI = confidence interval. Model 1–adjusted for chronological age and sex; Model 2–adjusted for chronological age, sex, ethnicity, cohabitation with spouse/partner, highest educational/professional qualification, annual gross household income, Townsend deprivation index and fasting time. For diagnoses printed in grey, there were no differences in sample size compared to the primary analysis. Cells highlighted in grey and blue correspond to statistically significant associations (nominally and after multiple testing corrections, respectively). *P*-values shown are corrected for multiple testing using the Benjamini–Hochberg procedure.

## 21. MileAge delta and groups of two-digit ICD-10 codes; smoking and BMI adjustment

**Table S20.** MileAge delta and mental/behavioural disorders; smoking and BMI adjustment

| | | Model 2 + smoking | | | | Model 2 + BMI | | | | Model 2 + smoking + BMI | | | |
|---|---|---|---|---|---|---|---|---|---|---|---|---|---|
| ICD-10 | *N* | *β* | 95% CI | | *p* | *β* | 95% CI | | *p* | *β* | 95% CI | | *p* |
| None | 186688 | Reference | | | | Reference | | | | Reference | | | |
| F10-99, Any disorder | 38524 | 0.199 | 0.156 | 0.241 | <0.001 | 0.138 | 0.097 | 0.179 | <0.001 | 0.117 | 0.075 | 0.158 | <0.001 |
| F10-19, Substance use | 11161 | 0.364 | 0.288 | 0.440 | <0.001 | 0.365 | 0.294 | 0.437 | <0.001 | 0.312 | 0.237 | 0.387 | <0.001 |
| F20-29, Psychosis | 587 | 0.550 | 0.244 | 0.855 | 0.002 | 0.442 | 0.140 | 0.744 | 0.011 | 0.419 | 0.117 | 0.722 | 0.017 |
| F30-39, Affective | 20360 | 0.214 | 0.159 | 0.269 | <0.001 | 0.094 | 0.039 | 0.148 | 0.002 | 0.082 | 0.027 | 0.136 | 0.010 |
| F40-48, Neurotic | 13916 | 0.119 | 0.054 | 0.184 | 0.001 | 0.080 | 0.016 | 0.144 | 0.033 | 0.072 | 0.008 | 0.136 | 0.056 |
| F50-59, Behavioural syndromes | 1763 | -0.222 | -0.399 | -0.046 | 0.032 | -0.185 | -0.359 | -0.011 | 0.069 | -0.194 | -0.368 | -0.020 | 0.056 |
| F60-69, Personality & behaviour | 433 | 0.198 | -0.156 | 0.553 | 0.360 | 0.051 | -0.298 | 0.400 | 0.825 | 0.033 | -0.316 | 0.382 | 0.879 |
| F70-79, Mental retardation | 14 | 1.457 | -0.509 | 3.423 | 0.216 | 1.419 | -0.516 | 3.354 | 0.216 | 1.437 | -0.498 | 3.372 | 0.216 |
| F80-89, Developmental | 115 | 0.335 | -0.351 | 1.022 | 0.429 | 0.275 | -0.409 | 0.960 | 0.507 | 0.279 | -0.405 | 0.964 | 0.507 |
| F90-98, Child & adolescent | 120 | 0.460 | -0.211 | 1.132 | 0.246 | 0.510 | -0.160 | 1.179 | 0.216 | 0.500 | -0.169 | 1.170 | 0.216 |
| F99, Unspecified | 86 | -0.061 | -0.855 | 0.732 | 0.879 | -0.207 | -0.988 | 0.574 | 0.664 | -0.224 | -1.005 | 0.557 | 0.654 |

*Note:* BMI = body mass index; ICD-10 = International Classification of Diseases, 10th Revision; CI = confidence interval. Model 2–adjusted for chronological age, sex, ethnicity, cohabitation with spouse/partner, highest educational/professional qualification, annual gross household income, Townsend deprivation index and fasting time. Cells highlighted in grey and blue correspond to statistically significant associations (nominally and after multiple testing corrections, respectively). *P*-values shown are corrected for multiple testing using the Benjamini–Hochberg procedure.

## 22. MileAge delta and individual two-digit ICD-10 codes; smoking and BMI adjustment

**Table S21.** MileAge delta and individual mental/behavioural disorders; smoking and BMI adjustment

| | | Model 2 + smoking | | | | Model 2 + BMI | | | | Model 2 + smoking + BMI | | | |
|---|---|---|---|---|---|---|---|---|---|---|---|---|---|
| ICD-10 | *N* | *β* | 95% CI | | *p* | *β* | 95% CI | | *p* | *β* | 95% CI | | *p* |
| None | 186688 | Reference | | | | Reference | | | | Reference | | | |
| F10, Alcohol use | 1541 | 0.484 | 0.294 | 0.675 | <0.001 | 0.474 | 0.287 | 0.661 | <0.001 | 0.436 | 0.248 | 0.624 | <0.001 |
| F11, Opioid use | 60 | 1.037 | 0.086 | 1.987 | 0.152 | 1.467 | 0.516 | 2.418 | 0.020 | 1.404 | 0.452 | 2.356 | 0.027 |
| F12, Cannabis use | 46 | -1.127 | -2.212 | -0.041 | 0.172 | -0.635 | -1.715 | 0.445 | 0.564 | -0.706 | -1.787 | 0.374 | 0.519 |
| F13, Sedative use | 68 | -0.492 | -1.384 | 0.400 | 0.564 | -0.334 | -1.212 | 0.544 | 0.720 | -0.360 | -1.238 | 0.518 | 0.710 |
| F14, Cocaine use | 10 | 0.700 | -1.626 | 3.026 | 0.838 | 1.158 | -1.132 | 3.447 | 0.612 | 1.085 | -1.205 | 3.374 | 0.636 |
| F15, Stimulant use | 14 | 0.759 | -1.207 | 2.724 | 0.720 | 0.959 | -0.975 | 2.894 | 0.612 | 0.941 | -0.993 | 2.876 | 0.621 |
| F16, Hallucinogen use | 12 | 3.630 | 1.507 | 5.753 | 0.010 | 3.393 | 1.303 | 5.483 | 0.015 | 3.344 | 1.254 | 5.434 | 0.015 |
| F17, Tobacco use | 9715 | 0.350 | 0.269 | 0.431 | <0.001 | 0.352 | 0.276 | 0.428 | <0.001 | 0.297 | 0.217 | 0.377 | <0.001 |
| F19, Multi-substance use | 81 | 0.430 | -0.388 | 1.248 | 0.601 | 0.633 | -0.182 | 1.448 | 0.411 | 0.580 | -0.235 | 1.395 | 0.478 |
| F20, Schizophrenia | 386 | 0.631 | 0.255 | 1.007 | 0.011 | 0.495 | 0.122 | 0.868 | 0.057 | 0.470 | 0.097 | 0.843 | 0.073 |
| F22, Delusional | 88 | 0.802 | 0.018 | 1.586 | 0.179 | 0.721 | -0.055 | 1.498 | 0.264 | 0.698 | -0.078 | 1.475 | 0.277 |
| F23, Acute psychosis | 66 | -0.058 | -0.963 | 0.848 | 0.954 | -0.136 | -1.027 | 0.755 | 0.905 | -0.143 | -1.034 | 0.749 | 0.905 |
| F25, Schizoaffective | 85 | 0.725 | -0.073 | 1.524 | 0.273 | 0.401 | -0.394 | 1.196 | 0.612 | 0.373 | -0.423 | 1.168 | 0.637 |
| F29, Psychosis (unspecific) | 83 | 0.349 | -0.458 | 1.157 | 0.692 | 0.339 | -0.456 | 1.134 | 0.692 | 0.319 | -0.476 | 1.115 | 0.710 |
| F30, Mania | 124 | 0.410 | -0.251 | 1.071 | 0.559 | 0.171 | -0.480 | 0.821 | 0.841 | 0.163 | -0.487 | 0.813 | 0.841 |
| F31, Bipolar | 711 | 0.138 | -0.139 | 0.415 | 0.612 | -0.006 | -0.279 | 0.268 | 0.968 | -0.024 | -0.298 | 0.249 | 0.954 |
| F32, Depressive episode | 19176 | 0.222 | 0.165 | 0.279 | <0.001 | 0.100 | 0.044 | 0.156 | 0.008 | 0.088 | 0.032 | 0.144 | 0.017 |
| F33, Recurrent depression | 1211 | 0.262 | 0.050 | 0.474 | 0.081 | 0.118 | -0.092 | 0.328 | 0.564 | 0.104 | -0.105 | 0.314 | 0.612 |
| F34, Persistent mood | 306 | 0.179 | -0.242 | 0.600 | 0.692 | 0.077 | -0.337 | 0.491 | 0.893 | 0.061 | -0.353 | 0.476 | 0.905 |
| F38, Mood (other) | 36 | 0.222 | -1.004 | 1.448 | 0.893 | 0.254 | -0.952 | 1.461 | 0.882 | 0.248 | -0.959 | 1.454 | 0.884 |
| F39, Mood (unspecific) | 282 | 0.162 | -0.277 | 0.600 | 0.729 | 0.026 | -0.407 | 0.460 | 0.954 | 0.013 | -0.420 | 0.447 | 0.964 |
| F40, Phobic | 717 | 0.106 | -0.169 | 0.381 | 0.720 | 0.052 | -0.219 | 0.323 | 0.893 | 0.038 | -0.233 | 0.309 | 0.909 |
| F41, Anxiety | 8433 | 0.093 | 0.010 | 0.175 | 0.131 | 0.072 | -0.009 | 0.153 | 0.278 | 0.064 | -0.017 | 0.146 | 0.395 |
| F42, Obsessive-compulsive | 270 | -0.587 | -1.035 | -0.139 | 0.060 | -0.601 | -1.042 | -0.160 | 0.048 | -0.603 | -1.044 | -0.162 | 0.048 |
| F43, Stress-related | 4749 | 0.165 | 0.057 | 0.273 | 0.021 | 0.099 | -0.008 | 0.205 | 0.264 | 0.092 | -0.014 | 0.199 | 0.304 |
| F44, Dissociative | 79 | 0.333 | -0.495 | 1.160 | 0.710 | -0.041 | -0.872 | 0.789 | 0.958 | -0.036 | -0.867 | 0.794 | 0.960 |
| F45, Somatoform | 843 | 0.016 | -0.238 | 0.269 | 0.954 | -0.009 | -0.260 | 0.241 | 0.964 | -0.013 | -0.263 | 0.238 | 0.958 |
| F48, Other neurotic | 487 | 0.035 | -0.299 | 0.368 | 0.951 | -0.039 | -0.369 | 0.290 | 0.940 | -0.050 | -0.380 | 0.279 | 0.905 |
| F50, Eating | 364 | -0.625 | -1.012 | -0.239 | 0.015 | -0.247 | -0.628 | 0.135 | 0.523 | -0.254 | -0.635 | 0.128 | 0.512 |
| F51, Sleep | 153 | 0.354 | -0.240 | 0.949 | 0.564 | 0.222 | -0.365 | 0.809 | 0.720 | 0.211 | -0.377 | 0.798 | 0.739 |
| F52, Sexual dysfunction | 632 | -0.024 | -0.318 | 0.269 | 0.954 | 0.020 | -0.270 | 0.309 | 0.954 | 0.008 | -0.281 | 0.297 | 0.964 |
| F53, Puerperal psychosis | 629 | -0.380 | -0.674 | -0.086 | 0.064 | -0.500 | -0.790 | -0.210 | 0.010 | -0.507 | -0.797 | -0.218 | 0.009 |

| | | | | | | | | | | | | | |
|---|---|---|---|---|---|---|---|---|---|---|---|---|---|
| F60, Personality | 180 | 0.658 | 0.109 | 1.207 | 0.094 | 0.395 | -0.147 | 0.937 | 0.459 | 0.373 | -0.169 | 0.915 | 0.500 |
| F63, Impulse | 16 | 1.072 | -0.766 | 2.910 | 0.564 | 1.042 | -0.767 | 2.852 | 0.564 | 1.014 | -0.796 | 2.823 | 0.564 |
| F66, Sexual development | 155 | -0.360 | -0.951 | 0.231 | 0.564 | -0.386 | -0.968 | 0.196 | 0.512 | -0.390 | -0.971 | 0.192 | 0.512 |
| F69, Personality (unspecific) | 64 | -0.101 | -1.021 | 0.818 | 0.948 | -0.238 | -1.143 | 0.667 | 0.841 | -0.270 | -1.175 | 0.635 | 0.838 |
| F80, Speech and language | 31 | 0.336 | -0.984 | 1.657 | 0.841 | 0.253 | -1.092 | 1.597 | 0.893 | 0.238 | -1.106 | 1.583 | 0.893 |
| F81, Scholastic skills | 60 | 0.256 | -0.695 | 1.206 | 0.841 | 0.223 | -0.720 | 1.166 | 0.851 | 0.231 | -0.712 | 1.174 | 0.843 |
| F84, Pervasive developmental | 18 | 0.373 | -1.360 | 2.107 | 0.882 | 0.468 | -1.238 | 2.175 | 0.841 | 0.479 | -1.227 | 2.186 | 0.841 |
| F90, ADHD | 10 | -0.377 | -2.703 | 1.948 | 0.905 | -0.156 | -2.445 | 2.133 | 0.954 | -0.190 | -2.479 | 2.099 | 0.954 |
| F91, Conduct | 43 | 1.215 | 0.094 | 2.337 | 0.152 | 1.174 | 0.070 | 2.278 | 0.162 | 1.151 | 0.047 | 2.255 | 0.172 |
| F93, Childhood emotional | 11 | 0.576 | -1.641 | 2.793 | 0.841 | 1.262 | -1.027 | 3.550 | 0.564 | 1.265 | -1.024 | 3.554 | 0.564 |
| F95, Tic | 11 | 1.561 | -0.656 | 3.778 | 0.482 | 1.627 | -0.555 | 3.809 | 0.452 | 1.606 | -0.576 | 3.789 | 0.457 |
| F98, Child or adolescent (other) | 45 | -0.615 | -1.712 | 0.481 | 0.564 | -0.638 | -1.742 | 0.466 | 0.564 | -0.636 | -1.740 | 0.468 | 0.564 |
| F99, Unspecified | 86 | -0.061 | -0.855 | 0.732 | 0.954 | -0.207 | -0.988 | 0.574 | 0.841 | -0.224 | -1.005 | 0.557 | 0.841 |

*Note:* BMI = body mass index; ICD-10 = International Classification of Diseases, 10th Revision; CI = confidence interval. Model 2–adjusted for chronological age, sex, ethnicity, cohabitation with spouse/partner, highest educational/professional qualification, annual gross household income, Townsend deprivation index and fasting time. Cells highlighted in grey and blue correspond to statistically significant associations (nominally and after multiple testing corrections, respectively). *P*-values shown are corrected for multiple testing using the Benjamini–Hochberg procedure.

## 23. MileAge delta and groups of two-digit ICD-10 codes; mutually exclusive

**Table S22.** MileAge delta and mental/behavioural disorders; mutually exclusive

| | | Model 1 (adj. age and sex) | | | | Model 2 (full adjustment) | | | |
|---|---|---|---|---|---|---|---|---|---|
| ICD-10 | | *β* | 95% CI | | *p* | *β* | 95% CI | | *p* |
| None | 186688 | Reference | | | | Reference | | | |
| F10-99, Any disorder | 30088 | 0.234 | 0.189 | 0.280 | <0.001 | 0.205 | 0.159 | 0.251 | <0.001 |
| F10-19, Substance use | 7636 | 0.429 | 0.343 | 0.515 | <0.001 | 0.395 | 0.309 | 0.482 | <0.001 |
| F20-29, Psychosis | 168 | 0.578 | 0.009 | 1.146 | 0.146 | 0.380 | -0.188 | 0.948 | 0.343 |
| F30-39, Affective | 13538 | 0.237 | 0.172 | 0.303 | <0.001 | 0.203 | 0.137 | 0.269 | <0.001 |
| F40-48, Neurotic | 7638 | 0.056 | -0.030 | 0.142 | 0.343 | 0.043 | -0.043 | 0.129 | 0.410 |
| F50-59, Behavioural syndromes | 841 | -0.137 | -0.392 | 0.117 | 0.410 | -0.125 | -0.379 | 0.129 | 0.410 |
| F60-69, Personality & behaviour | 131 | 0.431 | -0.212 | 1.074 | 0.343 | 0.426 | -0.217 | 1.069 | 0.343 |
| F70-79, Mental retardation | | *N* insufficient | | | | | | | |
| F80-89, Developmental | 57 | 0.180 | -0.795 | 1.155 | 0.752 | 0.117 | -0.857 | 1.092 | 0.813 |
| F90-98, Child & adolescent | 60 | 0.513 | -0.437 | 1.463 | 0.410 | 0.490 | -0.460 | 1.439 | 0.410 |
| F99, Unspecified | 14 | 0.473 | -1.494 | 2.441 | 0.738 | 0.401 | -1.564 | 2.367 | 0.752 |

*Note:* ICD-10 = International Classification of Diseases, 10th Revision; CI = confidence interval. Model 1–adjusted for chronological age and sex; Model 2–adjusted for chronological age, sex, ethnicity, cohabitation with spouse/partner, highest educational/professional qualification, annual gross household income, Townsend deprivation index and fasting time. Cells highlighted in grey and blue correspond to statistically significant associations (nominally and after multiple testing corrections, respectively). *P*-values shown are corrected for multiple testing using the Benjamini–Hochberg procedure.

## 24. MileAge delta and individual two-digit ICD-10 codes; mutually exclusive

**Table S23.** MileAge delta and mental/behavioural disorders; mutually exclusive

| | | Model 1 (adj. age and sex) | | | | Model 2 (full adjustment) | | | |
|---|---|---|---|---|---|---|---|---|---|
| ICD-10 | *N* | *β* | 95% CI | | *p* | *β* | 95% CI | | *p* |
| None | 186688 | Reference | | | | Reference | | | |
| F10, Alcohol use | 758 | 0.687 | 0.419 | 0.955 | <0.001 | 0.607 | 0.339 | 0.876 | <0.001 |
| F11, Opioid use | 17 | -0.141 | -1.927 | 1.644 | 0.948 | -0.348 | -2.132 | 1.436 | 0.884 |
| F12, Cannabis use | 11 | -0.861 | -3.080 | 1.358 | 0.732 | -1.069 | -3.287 | 1.149 | 0.650 |
| F13, Sedative use | 15 | -1.221 | -3.121 | 0.680 | 0.508 | -1.243 | -3.141 | 0.656 | 0.507 |
| F14, Cocaine use | | *N* insufficient | | | | | | | |
| F15, Stimulant use | | *N* insufficient | | | | | | | |
| F16, Hallucinogen use | | *N* insufficient | | | | | | | |
| F17, Tobacco use | 6662 | 0.396 | 0.304 | 0.488 | <0.001 | 0.370 | 0.278 | 0.462 | <0.001 |
| F19, Multi-substance use | 24 | 1.464 | -0.039 | 2.966 | 0.285 | 1.356 | -0.145 | 2.858 | 0.361 |
| F20, Schizophrenia | 121 | 0.508 | -0.161 | 1.177 | 0.460 | 0.291 | -0.379 | 0.960 | 0.686 |
| F22, Delusional | | *N* insufficient | | | | | | | |
| F23, Acute psychosis | 10 | 0.455 | -1.872 | 2.783 | 0.884 | 0.417 | -1.909 | 2.742 | 0.884 |
| F25, Schizoaffective | | *N* insufficient | | | | | | | |
| F29, Psychosis (unspecific) | | *N* insufficient | | | | | | | |
| F30, Mania | 13 | -0.116 | -2.158 | 1.925 | 0.954 | -0.139 | -2.179 | 1.901 | 0.951 |
| F31, Bipolar | 229 | 0.063 | -0.424 | 0.550 | 0.894 | 0.014 | -0.473 | 0.500 | 0.970 |
| F32, Depressive episode | 12357 | 0.237 | 0.168 | 0.305 | <0.001 | 0.202 | 0.134 | 0.271 | <0.001 |
| F33, Recurrent depression | 208 | 0.228 | -0.282 | 0.739 | 0.686 | 0.199 | -0.312 | 0.709 | 0.732 |
| F34, Persistent mood | 91 | 0.570 | -0.201 | 1.342 | 0.460 | 0.562 | -0.209 | 1.333 | 0.460 |
| F38, Mood (other) | | *N* insufficient | | | | | | | |
| F39, Mood (unspecific) | 90 | 0.518 | -0.258 | 1.294 | 0.507 | 0.512 | -0.263 | 1.288 | 0.507 |
| F40, Phobic | 279 | 0.076 | -0.365 | 0.517 | 0.884 | 0.064 | -0.377 | 0.505 | 0.890 |
| F41, Anxiety | 3747 | 0.046 | -0.075 | 0.168 | 0.732 | 0.033 | -0.088 | 0.155 | 0.884 |
| F42, Obsessive-compulsive | 61 | -0.525 | -1.468 | 0.417 | 0.585 | -0.554 | -1.495 | 0.388 | 0.548 |
| F43, Stress-related | 2281 | 0.126 | -0.029 | 0.281 | 0.433 | 0.114 | -0.041 | 0.269 | 0.460 |
| F44, Dissociative | 33 | 1.095 | -0.186 | 2.377 | 0.413 | 1.052 | -0.228 | 2.333 | 0.433 |
| F45, Somatoform | 486 | -0.070 | -0.404 | 0.264 | 0.884 | -0.063 | -0.397 | 0.271 | 0.884 |
| F48, Other neurotic | 174 | 0.129 | -0.430 | 0.687 | 0.884 | 0.080 | -0.478 | 0.638 | 0.890 |
| F50, Eating | 144 | -0.825 | -1.439 | -0.211 | 0.073 | -0.820 | -1.433 | -0.207 | 0.073 |
| F51, Sleep | 66 | 0.106 | -0.801 | 1.012 | 0.901 | 0.128 | -0.778 | 1.033 | 0.890 |
| F52, Sexual dysfunction | 351 | 0.136 | -0.257 | 0.529 | 0.765 | 0.144 | -0.249 | 0.537 | 0.743 |
| F53, Puerperal psychosis | 274 | -0.215 | -0.661 | 0.230 | 0.650 | -0.195 | -0.640 | 0.250 | 0.686 |
| F60, Personality | 25 | 1.734 | 0.262 | 3.206 | 0.126 | 1.706 | 0.235 | 3.177 | 0.126 |
| F63, Impulse | | *N* insufficient | | | | | | | |
| F66, Sexual development | 71 | -0.444 | -1.318 | 0.430 | 0.650 | -0.432 | -1.305 | 0.441 | 0.650 |
| F69, Personality (unspecific) | 17 | 1.090 | -0.695 | 2.875 | 0.544 | 1.071 | -0.713 | 2.855 | 0.544 |
| F80, Speech and language | 21 | 0.422 | -1.184 | 2.028 | 0.884 | 0.385 | -1.220 | 1.989 | 0.884 |
| F81, Scholastic skills | 26 | 0.046 | -1.397 | 1.490 | 0.970 | -0.015 | -1.458 | 1.428 | 0.984 |
| F84, Pervasive developmental | | *N* insufficient | | | | | | | |
| F90, ADHD | | *N* insufficient | | | | | | | |
| F91, Conduct | 16 | 2.238 | 0.397 | 4.078 | 0.124 | 2.203 | 0.365 | 4.042 | 0.124 |
| F93, Childhood emotional | | *N* insufficient | | | | | | | |
| F95, Tic | | *N* insufficient | | | | | | | |
| F98, Child or adolescent (other) | 26 | -1.027 | -2.470 | 0.417 | 0.468 | -1.057 | -2.499 | 0.385 | 0.460 |
| F99, Unspecified | 14 | 0.473 | -1.494 | 2.441 | 0.884 | 0.401 | -1.564 | 2.367 | 0.884 |

*Note:* ICD-10 = International Classification of Diseases, 10th Revision; CI = confidence interval. Model 1–adjusted for chronological age and sex; Model 2–adjusted for chronological age, sex, ethnicity, cohabitation with spouse/partner, highest educational/professional qualification, annual gross household income, Townsend deprivation index and fasting time. For diagnoses printed in grey, there were no differences in sample size compared to the primary analysis. Cells highlighted in grey and blue correspond to statistically significant associations (nominally and after multiple testing corrections, respectively). *P*-values shown are corrected for multiple testing using the Benjamini–Hochberg procedure.

25. MileAge delta and psychiatric comorbidity scores

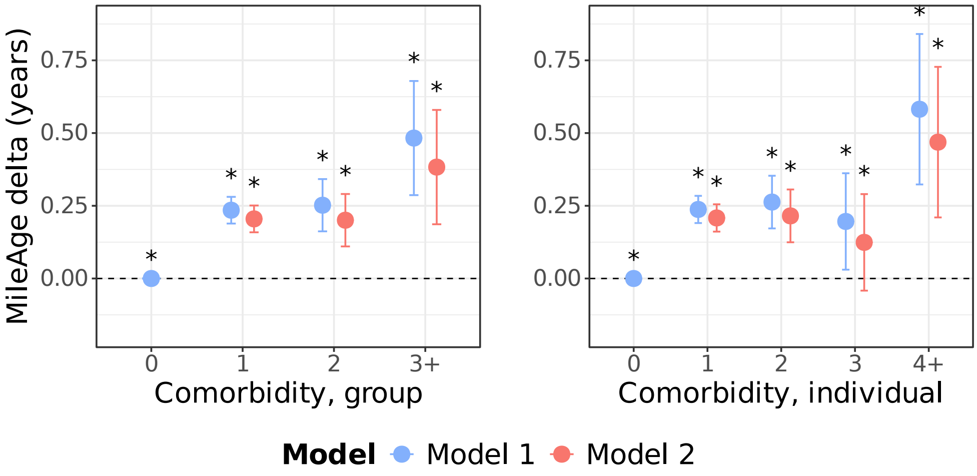


**Figure S12.** Associations between MileAge delta and mental/behavioural disorder comorbidity scores, based on groups of two-digit ICD-10 codes and individual two-digit ICD-10 codes, respectively. Betas and 95% confidence intervals were estimated using linear regression models. Reference group: individuals without mental/behavioural disorders. Model 1–adjusted for chronological age and sex; Model 2–adjusted for chronological age, sex, ethnicity, cohabitation with spouse/partner, highest educational/professional qualification, annual gross household income, Townsend deprivation index and fasting time. Asterisks indicate statistical significance after correcting *p*-values for multiple testing using the Benjamini–Hochberg procedure.

**Table S24.** MileAge delta and psychiatric comorbidity scores

| | | Model 1 | | | | Model 2 | | | |
|---|---|---|---|---|---|---|---|---|---|
| Comorbidity score | *N* | *β* | 95% CI | | *p* | *β* | 95% CI | | *p* |
| *Groups ICD-10 codes* | | | | | | | | | |
| None | 186688 | | | | | | | | |
| 1 | 30088 | 0.235 | 0.189 | 0.281 | <0.001 | 0.205 | 0.159 | 0.251 | <0.001 |
| 2 | 7010 | 0.252 | 0.162 | 0.342 | <0.001 | 0.200 | 0.110 | 0.291 | <0.001 |
| 3+ | 1426 | 0.483 | 0.287 | 0.679 | <0.001 | 0.383 | 0.186 | 0.580 | <0.001 |
| *Individual ICD-10 codes* | | | | | | | | | |
| None | 186688 | | | | | | | | |
| 1 | 28806 | 0.237 | 0.191 | 0.284 | <0.001 | 0.208 | 0.161 | 0.255 | <0.001 |
| 2 | 6896 | 0.263 | 0.172 | 0.353 | <0.001 | 0.215 | 0.125 | 0.306 | <0.001 |
| 3 | 2004 | 0.196 | 0.030 | 0.362 | <0.001 | 0.124 | -0.042 | 0.290 | <0.001 |
| 4+ | 818 | 0.582 | 0.323 | 0.841 | <0.001 | 0.469 | 0.210 | 0.728 | <0.001 |

*Note:* ICD-10 = International Classification of Diseases, 10th Revision; CI = confidence interval. Betas and 95% confidence intervals were estimated using linear regression models. Reference group: individuals without mental/behavioural disorders. Model 1–adjusted for chronological age and sex; Model 2–adjusted for chronological age, sex, ethnicity, cohabitation with spouse/partner, highest educational/professional qualification, annual gross household income, Townsend deprivation index and fasting time. Asterisks indicate statistical significance after correcting *p*-values for multiple testing using the Benjamini–Hochberg procedure.
